# Psychological Characteristics of Fathers of People With Bulimia Nervosa: A Systematic Review

**DOI:** 10.1002/eat.24333

**Published:** 2024-11-26

**Authors:** M. N. Akkese, J. L. Keeler, J. Y. Teh, J. Treasure, H. Himmerich

**Affiliations:** ^1^ Centre for Research in Eating and Weight Disorders (CREW), Department of Psychological Medicine, Institute of Psychiatry, Psychology and Neuroscience King's College London UK; ^2^ Department of Psychology, School of Mental Health & Psychological Sciences, Institute of Psychiatry, Psychology and Neuroscience King's College London UK; ^3^ South London and Maudsley NHS Foundation Trust Bethlem Royal Hospital Beckenham UK

**Keywords:** bulimia nervosa, fathers, paternal factors, personality, psychopathology, systematic review

## Abstract

**Objective:**

Bulimia nervosa (BN) is a highly prevalent psychiatric disorder that has negative effects on the physical and psychological health of sufferers. Father‐specific factors have been understudied in the context of BN. This systematic review aims to understand the psychosocial and psychopathological features of fathers of people with BN and their associations with the offspring's outcomes.

**Method:**

A literature search on APA PsycINFO, ISI Web of Science, PubMed, Google Scholar, ResearchGate, and Open Science Framework yielded 2421 studies. These papers were independently evaluated based on the eligibility criteria. 29 studies were included in this review. The Joanna Briggs Institute Critical Appraisal Tools were used for the assessment of the methodological quality of the eligible studies.

**Results:**

Across studies, no significant differences were found in perceptual body‐size distortion, self‐ideal discrepancy, eating‐/weight‐/body‐related attitudes, several personality and ED traits, and general psychological functioning between fathers of the BN group and those of the comparison groups. However, significant differences were found in certain psychological aspects (e.g., impulse regulation) and ED‐associated features (e.g., body dissatisfaction). Finally, significant relationships were found between the fathers' food attitudes, muscularity ratings, personality traits, and substance abuse and their offspring's risk of developing BN, greater body dissatisfaction, ED symptoms, and poor end‐of‐treatment outcome.

**Discussion:**

Although the existing literature does not seem to strongly suggest a particular paternal factor pertaining to BN, several father‐specific variables may be associated with the offspring's BN symptomatology and related characteristics. Further research is necessary to clearly understand paternal features in the context of BN.

**Summary**

- This systematic review established no remarkable differences between fathers of the bulimia nervosa (BN) and comparison groups in several eating/weight/body‐related attitudes and psychological variables. However, fathers of the BN group showed certain psychological and ED features.
- Fathers' food/body‐related factors, personality traits, and substance misuse were associated with their offspring's BN‐related outcomes.
- The obtained findings highlight the necessity of the in‐depth consideration of paternal psychological characteristics in the BN context.

## 1 Introduction

Bulimia Nervosa (BN) is characterized mainly by repetitive binge‐eating episodes, compensatory behaviors, and distorted body image perception (American Psychiatric Association 2013). BN is associated with a number of medical, psychological, and social problems (Castillo and Weiselberg 2017; Lin and Stone 2015). People with BN frequently have concurrent comorbid psychiatric disorders (e.g., anxiety and mood disorders, attention deficit hyperactivity disorder, substance and alcohol misuse) (Godart et al. 2015; Hudson et al. 2007; Nazar et al. 2016), and/or other psychological problems (e.g., self‐injurious behaviors, suicidal ideations, and suicide attempts) (Yao et al. 2016; Zerwas et al. 2015).

The etiology of BN is multifactorial, and existing models take a biopsychosocial approach (Treasure, Duarte, and Schmidt 2020). A genetic susceptibility, individual characteristics (e.g., low self‐esteem, impulsivity, and perfectionism), and sociocultural factors (e.g., peer bullying, judgmental comments about body‐shape, social media, and internalization of an unrealistic thinness ideal) are considered to be relevant risk factors (Bulik et al. 2022; Cheng et al. 2023; Fosse and Holen 2006; Forney, Holland, and Keel 2012; Hinney and Volckmar 2013; Krauss, Dapp, and Orth 2023; Lie et al. 2021; Lozano‐Madrid et al. 2023; Tafà et al. 2017; Thompson et al. 2023; Treasure, Duarte, and Schmidt 2020). Family characteristics (e.g., family dynamics and/or relationships, food environment, attitudes toward the offspring's eating, weight, and appearance) may also contribute to the development and maintenance of BN by interacting with other biological and environmental factors (Cerniglia et al. 2017; Hazzard et al. 2020; Latzer, Lavee, and Gal 2009; Rorty et al. 2000). The salient influences of parents include several aspects of the family life, such as individual traits and parenting relationships (Fassino, Amianto, and Abbate‐Daga 2009; Rienecke et al. 2016).

The available literature has explored maternal and paternal psychosocial variables (e.g., personality and psychopathological characteristics, caregiving styles, and eating‐ and weight‐related attitudes) pertaining to BN in the offspring. While some findings of these studies suggested similar maternal and paternal patterns among the comparison groups, other findings diverged for mothers and fathers. For example, neither mothers nor fathers of the participants with BN differed from the comparison groups with respect to their self‐ and ideal body perceptions, dieting, or weight history (Kanakis and Thelen 1995; Moreno and Thelen 1993). On the other hand, mothers in the BN group reported lower self‐directedness (as a personality characteristic), but higher levels of psychological problems (e.g., suicide attempts) and ED‐related traits (e.g., maturity fears) than mothers in the control or other ED groups (Fassino et al. 2003; Gómez‐Castillo et al. 2018; Pisetsky et al. 2017). Rather, lower persistence (i.e., perseverance in spite of fatigue and frustration as a personality trait) and more difficulties in impulse regulation (as an ED‐related trait) have been reported in fathers of people with BN compared to fathers of controls or individuals with other EDs (Fassino et al. 2003; Fassino, Amianto, and Abbate‐Daga 2009; Gómez‐Castillo et al. 2018).

Overall, the evidence presented indicates that individual paternal variables may have different relationships with the offspring's BN symptomatology from maternal ones. However, no systematic review has specifically examined the psychological characteristics of fathers. Therefore, the objective of this ‑systematic review was to understand (i) the psychosocial and psychopathological features of fathers of people with BN and (ii) their possible associations with the offspring's BN and related outcomes, in terms of the father's own psychopathology, personality, eating‐ and weight‐related attitudes and behaviors.

## 2 Method

This systematic review was conducted according to the preferred reporting items for systematic reviews and meta‐analysis (PRISMA) guidelines (Page et al. 2021). The study protocol was registered with the Open Science Framework (OSF; available at https://osf.io/je6nq).

### 2.1 Literature Search and Selection Process

Three electronic databases (APA PsycINFO, ISI Web of Science, and PubMed) and the first 20 pages of three additional databases (Google Scholar, ResearchGate, and OSF) were searched to obtain peer‐reviewed articles and gray literature materials (i.e., theses, conference proceedings, and pre‐print manuscripts) from inception until September 6, 2024. Utilizing Boolean operators (AND/OR) and truncation, the search terms included “father” or “dad” or “paternal” or “stepfather” in combination with “bulimia” or “bulimia nervosa.” Individual search strategies per database can be found in Table S1.

Search records in the electronic databases were imported into EndNote 20 and duplicates were removed. The remaining search records were exported to Rayyan for titles/abstracts screening and full‐text appraisal based on the eligibility criteria. Two authors (M.N.A. and J.Y.T.) independently screened the manuscripts, and disagreements were resolved with another author (J.L.K.).

### 2.2 Eligibility Criteria

The selection criteria for this systematic review were as follows:

#### 2.2.1 Inclusion Criteria

- Studies with participants with a primary diagnosis of BN based on a clinical diagnosis, clinical interview, or self‐report questionnaires applied in a clinical setting.
- Specific information about participants with BN and their fathers is provided.
- The study reports findings about a father‐specific variable (i.e., a psychopathological, personality, body‐image, weight or eating‐behavior‐related variables).
- This variable is rated by the father themselves, offspring with BN, or a clinician.
- Original peer‐reviewed articles, unpublished theses, conference proceedings, or pre‐print manuscripts.

#### 2.2.2 Exclusion Criteria

- Studies that do not contain any information on the method by which the diagnosis of BN in offspring is established (i.e., a clinical diagnosis, a clinical interview, or a self‐report scale).
- Studies with broadly‐defined BN samples.
- Studies that may have reported sample data in a previous publication.
- Statistical or clinical significance of the findings are not reported.
- Review articles (e.g., narrative literature, scoping, umbrella reviews), meta‐analyses, perspective papers, editorials, correction papers, letters without original data, book chapters without original data, qualitative studies, and case reports/series.
- Studies published in languages other than English.
- Studies with non‐human participants.

### 2.3 Data Extraction

The data relating to the research question were extracted from all the included studies into a spreadsheet on Microsoft Excel by the first author (M.N.A.), which were then checked by another author (J.L.K.). The extracted variables were: authors and publication year, the primary aim(s) of the study, demographic and clinical information of the relevant participants (i.e., sample size, age [mean ± standard deviation; SD], sex, race, ethnicity, socioeconomic status [SES], and diagnostic procedure of patients with BN, sample size and age [mean ± SD], race, ethnicity, and SES of fathers), the total number of participants in the study, the types of family members studied, the relevant data collection tools and who rated them, research methodology, study design, comparison groups, relevant findings regarding paternal psychosocial and psychopathological variables and their associations with the offspring's outcomes, and their statistical significance (i.e., *p*‐value).

### 2.4 Quality Assessment

The quality of the included studies was assessed using the Joanna Briggs Institute (JBI) (The Joanna Briggs Institute, n.d.) Critical Appraisal Tools (available at https://jbi.global/critical‐appraisal‐tools). This process was primarily completed by the first author (M.N.A.), and the quality assessment of each study was discussed with the second author (J.L.K.) to reconcile potential assessment discrepancies. See Tables S2.1–S2.3 for a full appraisal of the quality of the studies.

### 2.5 Data Synthesis

**FIGURE 1:**
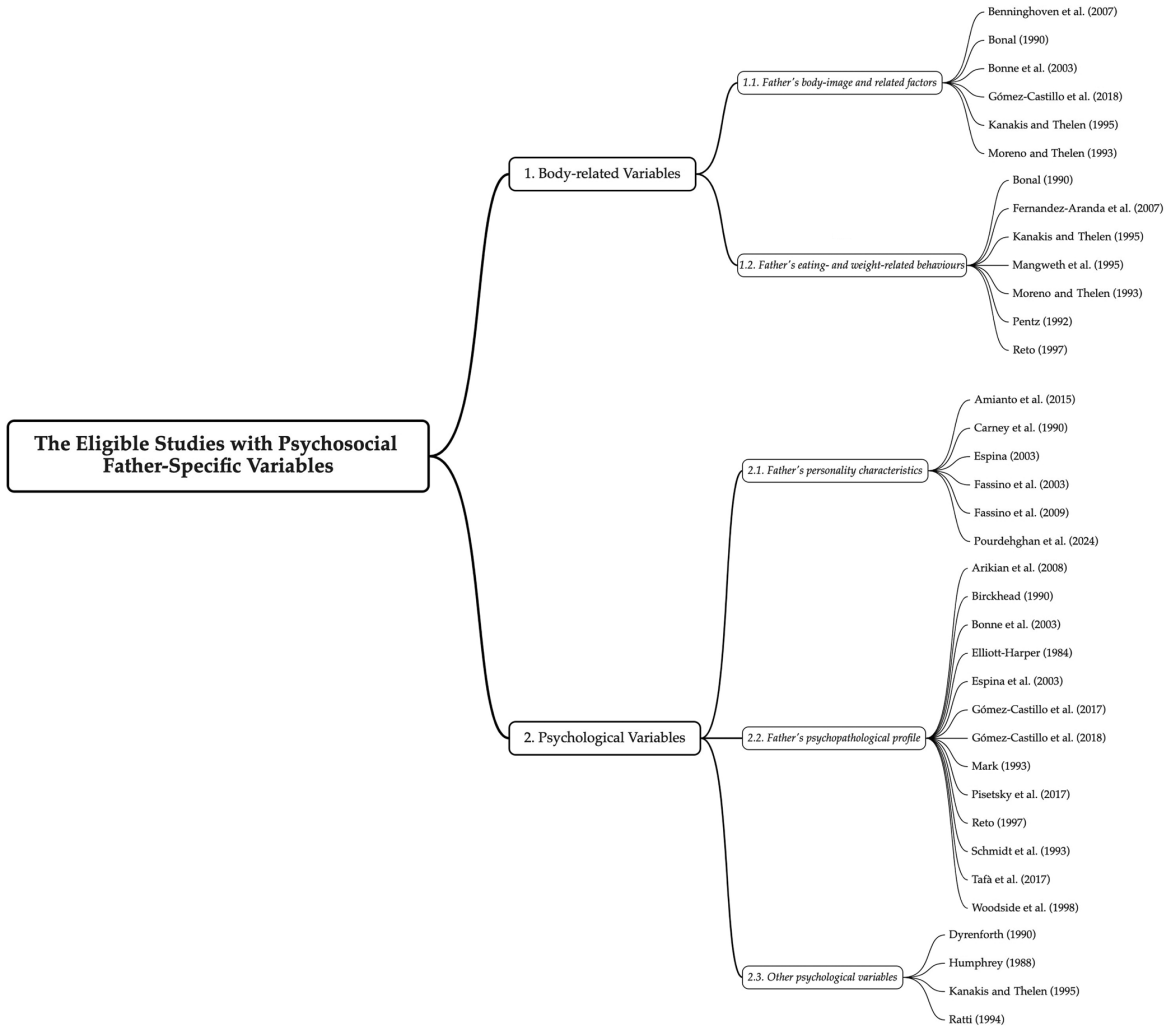
Data synthesis process.

The included studies were grouped into two main themes (body‐related variables; psychological variables) based on the content of paternal variables, which were subdivided into five subthemes, and the relevant findings were narratively synthesized. This process is visualized in Figure 1.

## 3 Results

### 3.1 Study Selection

**FIGURE 2:**
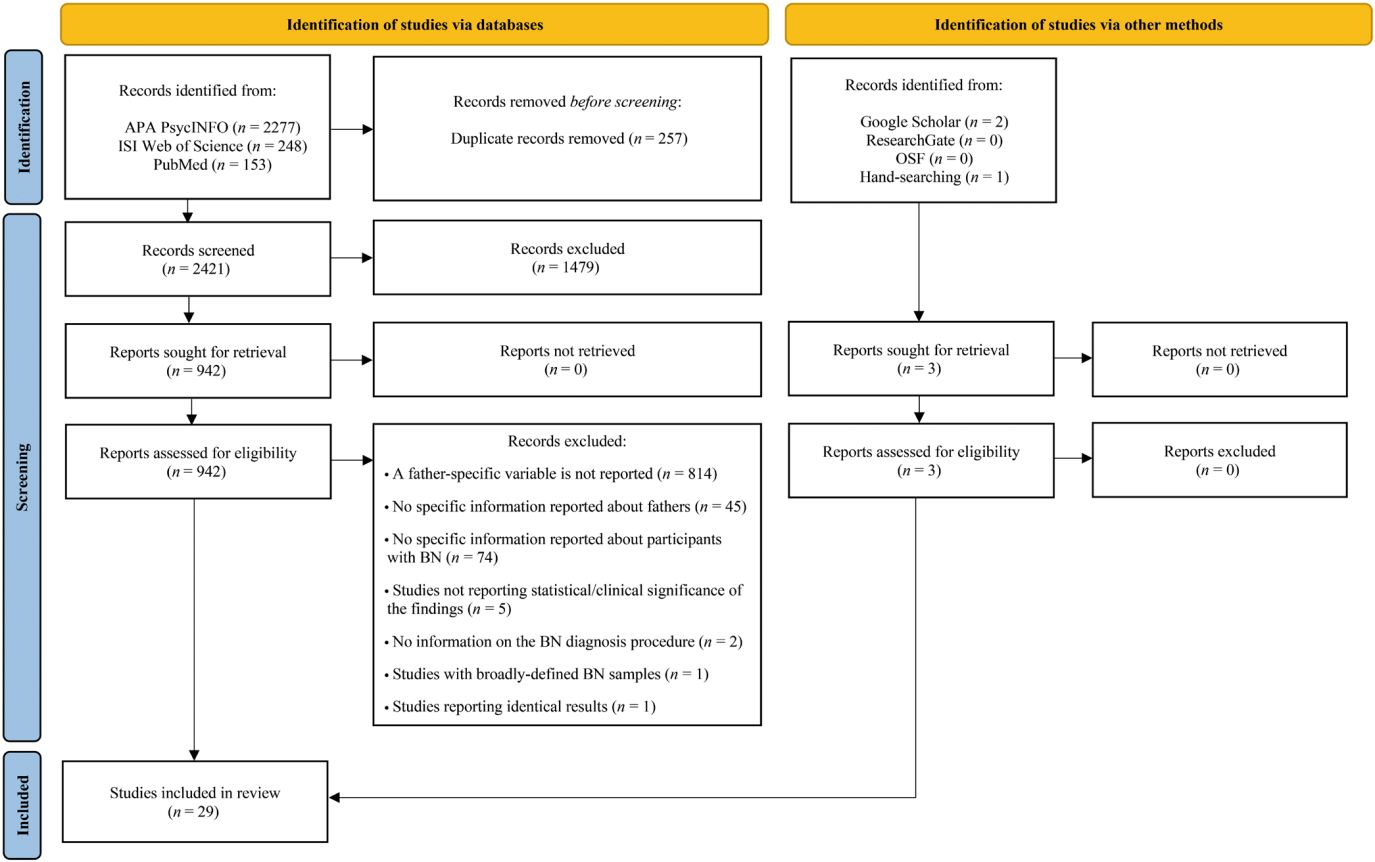
PRISMA flow‐chart of the study selection process (adapted from Page et al. 2021).

The database literature search on APA PsycINFO, ISI Web of Science, and PubMed identified 2678 records. Two studies were determined through the additional database search on Google Scholar, ResearchGate, and OSF, and one paper was identified through manual searching of the reference lists. 257 duplications were removed from the data on EndNote 20 and Rayyan. After the titles/abstracts were reviewed, full‐texts of the remaining studies were screened, and 2395 studies were excluded based on the eligibility criteria. A total of 29 studies were eligible for inclusion (Figure 2).

### 3.2 Sample Characteristics

Regarding the sociodemographic characteristics of participants with BN, the majority of the studies (*n* = 16, 55%) were performed with the BN samples in late adolescence or early adulthood. The pooled mean age (SD) of the offspring with BN was 22.53 ± 5.59 (reported in *n* = 16, 55%). 24 (~83%) out of 29 studies that reported participants' sex for the BN groups were conducted with only female participants with BN, whereas one study (~4%) recruited both females and males with BN (Gómez‐Castillo et al. 2018). Four studies (~14%) did not report participants' sex.

Only eight (~28%) out of 29 eligible studies gave information about the race of participants with BN; in seven of them, the race of the study sample was reported as (mostly) Caucasian/White (Arikian et al. 2008; Birckhead 1990; Elliott‐Harper 1984; Pentz 1992; Ratti 1994; Reto 1997; Tafà et al. 2017), and in one study, the race of participants was reported as White and Black (Mangweth, Pope, and Hudson 1995). Similarly, only two studies (~7%) provided information about the ethnicity of participants with BN; in one of them, participants were native‐born American and Austrian (Mangweth, Pope, and Hudson 1995), and in another study, participants were Iranian (Pourdehghan et al. 2024). Two studies (~7%) reported the SES of offspring with BN; in one of them, most participants had at least middle SES (Pentz 1992), and in another study, most participants were not in employment (Schmidt, Tiller, and Treasure 1993). Four studies (~14%) reported the family SES of the BN group as mostly middle class (Gómez‐Castillo et al. 2018; Moreno and Thelen 1993; Ratti 1994; Tafà et al. 2017). One study (3.5%) did not provide detailed information about the family SES of the BN sample (Elliott‐Harper 1984). The remaining 22 studies (~76%) did not give information about the SES of the BN groups or their families.

Concerning the sociodemographic characteristics of fathers, most eligible studies (*n* = 17, ~59%) recruited fathers as well as the participants with BN. In the seven (24%) studies that reported the mean age (SD) and the number of the fathers of the BN sample, the pooled mean age ± SD was 54.71 ± 8.53, meaning that fathers were mostly in middle adulthood. Only one paper (~6%) out of 17 studies reported the fathers' own SES as middle (Moreno and Thelen 1993), but another study (~6%) did not report detailed information about the fathers' own SES (Elliott‐Harper 1984). Three studies (~18%) reported the family SES, which was broadly middle class (Gómez‐Castillo et al. 2018; Ratti 1994; Tafà et al. 2017).

### 3.3 Study Characteristics

To establish the BN diagnosis in the offspring, most studies (*n* = 24, ~83%) utilized a clinical assessment and a structured or semi‐structured clinical interview. While three studies (10%) used a self‐report scale applied for the participants who were recruited from clinical settings (Bonal 1990; Pentz 1992; Pisetsky et al. 2017), two studies (~7%) utilized a self‐report scale along with a diagnostic interview (Kanakis and Thelen 1995; Moreno and Thelen 1993).

25 (86%) out of 29 eligible studies employed cross‐sectional group comparisons to investigate father‐specific psychosocial variables between the BN and other groups (*n* = 24) or between the BN groups in different cultures (*n* = 1). The comparator groups in 24 studies included participants with anorexia nervosa (AN; *n* = 8, 33%), anorexia nervosa‐restricting type (AN‐R; *n* = 5, ~21%), anorexia nervosa‐binge‐eating/purging type (AN‐BP; *n* = 5, ~21%), binge‐eating disorder (BED; *n* = 2, 8%), ED not otherwise specified (EDNOS; *n* = 4, ~17%), subclinical BN (S‐BN; *n* = 2, 8%), purging disorder (*n* = 1, 4%), “weight‐obsessed” (*n* = 1, 4%), and control participants assessed for only BN or ED symptoms (non‐BN/ED CG; *n* = 5, ~21%), control participants with (psychiatric control [PC]; *n* = 4, ~17%) and without (healthy control [HC]; *n* = 12, 50%) a psychiatric diagnosis or problem. In addition to including other comparison groups, two studies (8%) also included participants with different BN characteristics (i.e., BN with versus without a history of AN, a recent versus chronic BN, and restricting versus normal weight BN on the basis of the participants' weight history) (Dyrenforth 1990; Schmidt, Tiller, and Treasure 1993). Finally, one study (4%) recruited only BN samples comparing them cross‐culturally (Austrian vs. American) (Mangweth, Pope, and Hudson 1995).

16 studies (55%) obtained data on fathers of participants with BN from the fathers themselves, whereas the relevant variables were rated by the offspring in 11 studies (~38%). In one study (3.5%), the paternal variable was reported by the offspring's therapist (Birckhead 1990), but another study did not clearly provide the relevant information (3.5%) even if it recruited both offspring and their fathers. With regards to the data collection tools, the majority (*n* = 25, 86%) of the studies used self‐report questionnaires, and the remaining studies (*n* = 4, 14%) utilized an (structured or semi‐structured) individual interview method (Elliott‐Harper 1990; Mangweth, Pope, and Hudson 1995; Schmidt, Tiller, and Treasure 1993; Woodside et al. 1998). For more detailed information, see Tables 1, 2, 3.

**TABLE 1:** The eligible studies with father‐specific body‐related variables—group comparisons in fathers.

Author(s) and year	The aim(s) of study	*N* and mean age (SD) (offspring with BN)	Sex	Race and ethnicity and SES	Diagnostic procedure	*N* and mean age (SD) (father)	Race and ethnicity and SES	Total *N*	Studied family Member (s)	Data collection tools^e^ and reported by	Research method and study design	Group comparisons	Main findings^f^, ^g^
Subtheme 1: Father's body‐image and related factors													
Benninghoven et al. (2007)	To assess the body image perception of the participants with EDs and their fathers	15 and 20.1 (3.1)	F	NR	SCID‐I for the DSM‐IV	15 and 50.2 (6.8)	NR	84 AN: 27, 27F BN:15, 15F	Offspring, fathers	Demographic information, BIA, The somatomorphic matrix, Perceptual body size distortion and self‐ideal discrepancy calculations and Fathers	Self‐report Quantitative and Cross‐sectional	Fathers of the BN group versus Fathers of the AN group	There was no significant difference in fathers' perceptual body size distortion in terms of both body‐fat (i.e., perceived minus actual percentage body‐fat) and muscularity (i.e., perceived minus actual percentage muscularity) between the BN and AN group (*p* = ns)
													Fathers' self‐ideal discrepancy regarding both body‐fat (i.e., perceived minus desired percentage body‐fat) and muscularity (i.e., perceived minus desired percentage muscularity) was similar in the groups, meaning that both father groups wished to be thinner (self‐ideal discrepancy—body fat) and more muscular (self‐ideal discrepancy—muscularity) (*p* = ns)
Bonal (1990)^a^	To investigate the normal weight BN patients' family environment and family beliefs and behaviors regarding food, eating, and weight	21 and NR The mean age of the total sample: 20.08 (1.7) The age range of the total sample: 18–26	F	NR	BULIT administered to participants recruited from clinical settings	—	—	80 BN: 21 PC: 25 HC: 34	Offspring	Demographic information, Questions about the family beliefs and behaviors regarding food, eating, dieting, and thinness and Offspring	Self‐report Quantitative and Cross‐sectional	The three groups (BN, PC, HC)	No significant differences were found among the offspring groups in terms of perceived paternal concern about his own weight (*p* > 0.05)
Bonne et al. (2003)	To examine family function, attitudes toward eating habits, body image, food, and inter‐personal relations in the family members To compare the outcomes between the groups	16 and 23.0 (2.0)	F	NR	Clinical assessment based on the DSM‐IV criteria	8 and 51.0 (7.0)	NR	80 BN: 16, 15 M, 8F HC^b^: 16, 15 M, 10F	Offspring, mothers, fathers	Demographic information, EDI and Fathers	Self‐report Quantitative and Cross‐sectional	Fathers of the BN group versus Fathers of the HC group	No significant differences were found on the body‐related subscales of the EDI^g^ (i.e., Drive for Thinness and Body Dissatisfaction) between the father groups (*p* = ns)
Gómez‐Castillo et al. (2018)	To simultaneously explore the differences in parents' and their offspring's ED symptoms	30 and 19.5 (5.12)	F + M (29F, 1 M)	NR Mostly middle family SES (36.7%)	SCID‐I for the DSM‐IV	24 and 49.4 (6.6)	NR Mostly middle family SES (36.7%)	348 AN: 30, 30 M, 28F BN: 30, 29 M, 24F EDNOS: 30, 30 M, 30F HC^b^: 30, 29 M, 28F	Offspring, mothers, fathers	A demographic questionnaire, EDI‐2 and Fathers	Self‐report Quantitative and Cross‐sectional	Fathers of the four groups (AN, BN, EDNOS, HC)	No significant differences were found among the four father groups on the Drive for Thinness subscale of the EDI‐2^g^ (*p* = ns)
												Fathers of the BN group versus Fathers of the EDNOS group	Fathers of the BN group had significantly higher scores on the Body Dissatisfaction subscale of the EDI‐2 than those of the EDNOS group (*p* = 0.016)
Kanakis and Thelen (1995)	To investigate whether offspring and parent variables were related to BN	36 and NR The mean age of the total proband sample: 18.9 (2.48)	F	NR^c^	BULIT‐R and The diagnostic interview ratings based on the DSM‐III‐R criteria	29 and NR The mean age of the total father sample: 47.5 (5.5)	NR	312 BN: 36, 34 M, 29F S‐BN: 39, 37 M, 34F Non‐BN CG^d^: 37, 32 M, 34F	Offspring, mothers, fathers	Demographic information, Tennessee Self‐Concept Scale, Body Image Scale, Body Esteem Scale, The Restraint and Disinhibition subscales of Three‐Factor Eating Questionnaire, History of Eating Questionnaire for parents and Fathers	Self‐report Quantitative and Cross‐sectional	Fathers of the three groups (BN, S‐BN, Non‐BN CG)	The three father groups did not show significant differences in terms of self‐ and ideal perceptions and body importance, when controlling for the paternal BMI (*p* = ns)
Moreno and Thelen (1993)	To examine the associations between parental factors and BN	24 and NR The data were collected from undergraduate students	F	NR Middle family SES	BULIT‐R and A structured diagnostic interview consisting of questions based on the DSM‐III‐R criteria	15 and NR	NR Middle SES	176 BN: 24, 17 M, 15F S‐BN: 23, 15 M, 11F Non‐BN CG^d^: 26, 23 M, 22F	Offspring, mothers, fathers	Demographic information, FHE‐P and Fathers	Self‐report Quantitative and Cross‐sectional	Fathers of the three groups (BN, S‐BN, Non‐BN CG)	With regard to fathers' attitudes toward themselves about being overweight, no significant difference was found on any relevant item of the FHE‐P among the three groups (*p* = ns)
Subtheme 2: Father's eating‐ and weight‐related behaviors													
Bonal (1990)^a^	To investigate the normal‐weight BN patients' family environment and family beliefs and behaviors regarding food, eating, and weight	21 and NR The mean age of the total sample: 20.08 (1.7) The age range of the total sample: 18–26	F	NR	BULIT administered to participants recruited from clinical settings	—	—	80 BN: 21 PC: 25 HC: 34	Offspring	Demographic information, Questions about the family beliefs and behaviors regarding food, eating, dieting, and thinness and offspring	Self‐report Quantitative and Cross‐sectional	The three groups (BN, PC, HC)	No significant differences were found among the groups in terms of paternal diet frequency (*p* > 0.05)
Kanakis and Thelen (1995)	To investigate whether offspring and parent variables were related to BN	36 and NR The mean age of the total proband sample: 18.9 (2.48)	F	NR^c^	BULIT‐R and The diagnostic interview ratings based on the DSM‐III‐R criteria	29 and NR The mean age of the total father sample: 47.5 (5.5)	NR	312 BN: 36, 34 M, 29F S‐BN: 39, 37 M, 34F Non‐BN CG^d^: 37, 32 M, 34F	Offspring, mothers, fathers	Demographic information, Tennessee Self‐Concept Scale, Body Image Scale, Body Esteem Scale, The Restraint and Disinhibition subscales of Three‐Factor Eating Questionnaire, History of Eating Questionnaire for parents and Fathers	Self‐report Quantitative and Cross‐sectional	Fathers of the three groups (BN, S‐BN, Non‐BN CG)	The three father groups did not show significant differences in terms of their eating behaviors and dieting, when controlling for father's BMI (*p* = ns)
Mangweth, Pope, and Hudson (1995)^a^	To compare two groups from different cultures in terms of a range of variables in the context of BN	66 and Austria 22.0 (2.2) USA.20.0 (2.0)	F	Austria Only White Native‐born Austrian NR USA 32 White, 1 Black Native‐born American NR	SCID‐I for the DSM‐III‐R	—	—	66 BN‐Austria: 33 BN‐USA.: 33	Offspring	Demographic information, Interview questions about preoccupation with weight of family members and Offspring	Semi‐structured Interview Quantitative and Cross‐sectional	The USA. BN group versus The Austria BN group	Fathers were reported to be significantly more likely to be dieting in the USA. than in Austria (*p* < 0.005)
Moreno and Thelen (1993)	To examine the associations between parental factors and BN	24 and NR The data were collected from undergraduate students	F	NR Middle family SES	BULIT‐R and A structured diagnostic interview consisting of questions based on the DSM‐III‐R criteria	15 and NR	NR Middle SES	176 BN: 24, 17 M, 15F S‐BN: 23, 15 M, 11F Non‐BN CG^d^: 26, 23 M, 22F	Offspring, mothers, fathers	Demographic information, FHE‐P and Fathers	Self‐report Quantitative and Cross‐sectional	Fathers of the three groups (BN, S‐BN, Non‐BN CG)	With regard to fathers' attitudes toward their own dieting, no significant differences were found on any relevant items of the FHE‐P among the three groups (*p* = ns)
Pentz (1992)^a^	To investigate developmental difficulties with the separation‐individuation process and dysfunction in the family‐of‐origin in the groups	40 and NR The age range of the total sample: 17–32	F	White NR Mostly at least middle class	BULIT administered to participants recruited from clinical settings	—	—	115 BN: 40 PC: 23 HC: 52	Offspring	The Personal Information Form including questions about the basic demographic information and weight and dieting data of the individual's family and Offspring	Self‐report Quantitative and Cross‐sectional	The three groups (BN, PC, HC)	No significant group differences were found in the rates of paternal dieting behavior (*p* > 0.05)
Reto (1997)^a^	To explore the relationships among childhood maltreatment, dissociation, and bulimia symptomatology	23 and 30.0 (8.0)	F	Mostly Caucasian NR	Clinical interview based on the EDE	—	—	130 BN: 23 Non‐BN CG^d^: 107	Offspring	Demographic information, A questionnaire about family psychiatric and ED‐related demographics and Offspring	Self‐report Quantitative and Cross‐sectional	The BN group versus The non‐BN CG	Fathers were reported to be significantly more likely to be on chronically dieting in the offspring BN group than in the non‐BN CG (*p* = 0.001)

Abbreviations: AN: anorexia nervosa; AN‐BP: anorexia nervosa—binging purging SUBTYPE; AN‐R: anorexia nervosa—restricting subtype; BDI: beck depression inventory; BED: binge‐eating disorder; BIA: bio‐impedance analysis; BMI: body mass index; BN: bulimia nervosa; CCQ: the cross‐cultural (environmental) questionnaire; DSM‐III: diagnostic and statistical manual of mental disorders, the third edition; DSM‐IV: diagnostic and statistical manual of mental disorders, the fourth edition; ED: eating disorder; EDI‐2: eating disorders inventory‐2; EDNOS: eating disorders not otherwise specified; F: father; F: female; HC: control group without a psychiatric diagnosis or problem; K‐SADS‐PL: kiddie schedule for affective disorders and schizophrenia—present and lifetime version; M: mother; MCMI‐III: millon clinical multiaxial inventory‐the third edition; *N*: number; NR: not reported; OR: odds ratio; PC: control group with a psychiatric diagnosis or problem; PD: personality disorder; PDBQ: a Psychiatric Data Base Questionnaire; SAS: The Self‐Rating Anxiety Scale; SCID‐I for the DSM‐III‐R/the DSM‐IV: the structured clinical interview for the DSM‐III‐R/the DSM‐IV axis‐I disorders; SD: standard deviation; SES: socioeconomic status; TCI: temperament character inventory.

^a^ Data collection tools regarding the father‐specific psychosocial variable were rated by offspring in this study.

^b^ The participants in the control group were stated as “healthy” or were assessed in terms of addictions, intellectual disability, or psychotic disorders; however, the screening criteria for BN were unclear for these participants.

^c^ The offspring groups did not differ in their socioeconomic status; however, no information was provided on the socioeconomic level corresponding to the overall mean value of the relevant analysis.

^d^ The participants in the control group were assessed for BN symptomatology; however, no information was provided whether these participants had other clinical conditions.

^e^ Only data collection tools used for the demographic and clinical data and the father‐specific variables are reported.

^f^ ns: non‐significant (i.e., There is no the exact *p*‐value information in the study).

^g^ *The subscales of the questionnaires*: EDI: Drive for Thinness, Bulimia, Body Dissatisfaction, Inefficiency, Perfectionism, Interpersonal Distrust, Lack of Self‐awareness, Fears of Maturity, Asceticism, Impulse Control, Lack of Confidence in Company; EDI‐2: Drive for Thinness, Bulimia, Body Dissatisfaction, Ineffectiveness, Perfectionism, Interpersonal Distrust, Interoceptive Awareness, Maturity Fears, Asceticism, Impulse Regulation, Social Insecurity.

**TABLE 2:** The eligible studies with father‐specific psychological variables—group comparisons in fathers.

Author(s) and year	The aim(s) of study	*N* and mean age (SD) (offspring with BN)	Sex	Race and ethnicity and SES	Diagnostic procedure	*N* and mean age (SD) (father)	Race and ethnicity and SES	Total *N*	Studied family member(s)	Data collection tools^f^ and reported by	Research method and study design	Group comparisons	Main findings^g^, ^h^
Subtheme 1: Father's personality characteristics													
Amianto et al. (2015)	To examine how parents' personality clusters are associated with their offspring's personality and eating psychopathology To determine whether there is a significant relationship between offspring's severe eating psychopathology in case of markedly altered parents' personality traits	31 and NR	F	NR	SCID‐I for the DSM‐IV‐TR	NR and NR The mean age of the total father sample: 51.6 (10.1)	NR	385 AN: 81 BN: 31 EDNOS:61 108 M, 104F	Offspring, mothers, fathers	Demographic information, TCI and Fathers	Self‐report Quantitative and Cross‐sectional	Fathers of the three ED groups (AN, BN, EDNOS)	The analysis on fathers' temperament traits (TCI)^h^ yielded two clusters (Explosive/Methodical and Independent/Methodical). No significant differences were found in the ED diagnosis distribution of offspring between the temperament clusters of fathers (*p* < 0.271)
													The analysis on fathers' character traits (TCI)^h^ yielded three clusters (Immature, Asocial, and Mature). No significant differences were found in the ED diagnosis distribution of offspring among the character clusters of fathers (*p* < 0.506)
Carney, Yates, and Cizadlo (1990)	To examine the prevalence of personality traits in first degree relatives of the groups to assess the role of familial personality pathology as a risk factor for normal weight BN without a history of AN	25 and NR The age range of the BN sample: 18–43	F	NR	SCID for the DSM‐III‐R	12 and NR	NR	157 BN family: 25 probands, 46 first degree relatives HC family: 25 probands, 61 first degree relatives	Offspring, first‐degree family members	Demographic information, PDQ‐R and Fathers	Self‐report Quantitative and Cross‐sectional	Fathers of the BN group versus Fathers of the HC group	No significant difference was found in the mean total PDQ scores between fathers of the BN group and those of the HC group (*p* = 0.390)
													Fathers of the BN group had an average of 2.42 pathological scores on the Histrionic subscale, meaning that they had nearly three times higher scores than fathers of the HCs (*p* = 0.036)
													On the scale‐item analysis, fathers of the BN group significantly differed on the questions “If I don't get my way, I get angry and behave childishly” (25% versus 0%) (*p* = 0.06) and “People say that I am self‐centered” (50% versus 5.9%) (*p* = 0.01) of the Histrionic subscale from fathers of HCs
Espina (2003)	To examine the probability of alexithymia in parents of patients with EDs by comparing them with the control participants To assess the relationships between alexithymia and psychopathologic and personality variables	30 and 19.67 (3.15)	F	NR	Clinical interview based on the DSM‐IV criteria	30 and 50.57 (6.26)	NR	435 AN‐R: 20, 20 M, 20F AN‐BP: 23, 23 M, 23F BN: 30, 30 M, 30F HC: 72, 72 M, 72F	Mothers, fathers	Demographic information, TAS‐20 and Fathers	Self‐report Quantitative and Cross‐sectional	Fathers of the three ED groups (AN‐R, AN‐BP, BN)	No significant differences were found among the three father groups on either the total TAS‐20 or its three subscales^h^ (*p* = ns)
												Fathers of the four groups (AN‐R, AN‐BP, BN, HC)	Significant differences were found among the four father groups in the total TAS‐20 (*p* = 0.02) and its subscale F3 (*p* = 0.006) in the univariate analysis. However, multiple group comparisons did not show group differences (*p* = ns)
													A significant difference was found on only the F3 subscale among the four father groups (*p* = 0.043), after the covariate adjustment of the BDI score on the TAS‐20 and its factors; however, this was not driven by the difference between the fathers of the BN group and those of the other groups (*p* = ns)
													No significant differences were found among the four father groups on either the total TAS‐20 or its subscales after the covariate adjustments of the BDI and SAS scores on the TAS‐20 and its factors (*p* = ns)
Fassino et al. (2003)	To assess the temperament and character traits of patients with BN and their parents To determine the correlation of temperament and character traits among family members in the BN group To investigate whether the tool of TCI distinguishes the healthy controls and patients with BN, their parents, and their families	28 and 21.7 (4.1)	F	NR	SCID‐I for the DSM‐IV	23 and 57.3 (7.6)	NR	164 BN: 28, 28 M, 23F HC: 29, 29 M, 27F	Offspring, mothers, fathers	Demographic information, TCI and Fathers	Self‐report Quantitative and Cross‐sectional	Fathers of the BN group versus Fathers of the HC group	In the temperament dimensions of the TCI^h^, fathers of the BN group reported lower Persistence than did fathers of the HCs (*p* = 0.011)
													No significant group differences were found on the other temperament subscales (*p* = ns)
													No significant differences were found on any subscale of the character dimensions of the TCI between fathers of the BN and HC groups (*p* = ns)
Fassino, Amianto, and Abbate‐Daga (2009)	To compare the personality traits and eating psychopathology of patients with EDs and their parents' personality characteristics with those of the control participants To explore the associations between parents' personality characteristics and their daughters' personality traits and eating psychopathology	37 and 25.59 (7.6)	F	NR	SCID‐I for the DSM‐III‐R	31 and 63.91 (2.5)	NR	447 AN‐R: 38, 32 M, 28F AN‐BP: 30, 29 M, 28F BN: 37, 36 M, 31F HC: 54, 54 M, 50F	Offspring, mothers, fathers	Demographic information, TCI, EDI‐2 and Fathers	Self‐report Quantitative and Cross‐sectional	Fathers of the four groups (AN‐R, AN‐BP, BN, HC)	No significant differences were found among the four father groups on the Novelty Seeking (*p* = 0.16), Reward Dependence (*p =* 0.26), Cooperativeness (*p* = 0.69), and Self‐transcendence (*p* = 0.82) subscales of the TCI^h^
													The groups were significantly different on the Harm Avoidance (*p* = 0.003) and Self‐directedness (*p* = 0.004) subscales of the TCI, although these were not driven by the differences between fathers of the BN group and those of any other group (*p* = ns)
												Fathers of the BN group versus Fathers of the HC group	The groups were significantly different on the Persistence (*p* = 0.003) subscale of the TCI; fathers of the BN group had significantly lower scores on the Persistence subscale of the TCI than did those of the HC group, even after the covariate adjustment for age (*p* < 0.001)
Subtheme 2: Father's psychopathological profile													
Birckhead (1990)^a^	To explore the differences among the groups in terms of several ED‐related and psychological variables	8 and NR The mean age of the total sample: 23.54 (NR) The age range of the total sample: 13–39	F	Caucasian NR	Clinical assessment based on the DSM‐III‐R criteria	—	—	24 AN‐R: 7 AN‐BP: 9 BN: 8	Offspring	Demographic information, Referral Source Questionnaire and Offspring's therapist	Self‐report Quantitative and Cross‐sectional	The three ED groups	Paternal anxiety (*p* = 0.23) and depression scores (*p* = 0.13) reported by the offspring's therapist did not show significant differences among the ED groups
Bonne et al. (2003)	To examine family function, attitudes toward eating habits, body image, food, and inter‐personal relations in the family members To compare the outcomes between the groups	16 and 23.0 (2.0)	F	NR	Clinical assessment based on the DSM‐IV criteria	8 and 51.0 (7.0)	NR	80 BN: 16, 15 M, 8F HC^b^: 16, 15 M, 10F	Offspring, mothers, fathers	Demographic information, EDI and Fathers	Self‐report Quantitative and Cross‐sectional	Fathers of the BN group versus Fathers of the HC group	No significant differences were found on any subscale of the EDI^h^ between the father groups (*p* = ns)
Elliott‐Harper (1984)	To compare the data concerning eating disorders and psychological or psychiatric disorders in the families of the ED patients with the families of a matched normal group	20 and 23.7 (7.5)	F	Caucasian NR Mostly family Class 4^c^	Clinical assessment based on the DSM‐III	20 and 54.9 (10.1)	Caucasian NR Mostly Class 4^c^	165 AN: 15, 15 M, 15F BN: 20, 20 M, 20F Non‐ED CG^d^: 20, 20 M, 20F	Offspring, mothers, fathers	An interview questionnaire about demographics, proband's and family's psychiatric history, A three‐generation pedigree and NR	Structured interview Quantitative and Cross‐sectional	The three offspring groups (AN, BN, Non‐ED CG)	No significant differences were found in the rates of fathers with “other psychiatric pathology” among the offspring groups (*p* > 0.05)
Gómez‐Castillo et al. (2017)	To examine the degree of psychopathology of ED patients and their parents To assess the relationship between these variables to use it as therapeutic tool	30 and 19.5 (5.12)	NR	NR	SCID‐I for the DSM‐IV	NR and 49.4 (6.6)	NR	339 AN:30 BN: 30 EDNOS: 30 HC: 30 Parents of ED groups: 139 Parents of the HC: 48	Offspring, mothers, fathers	A demographic questionnaire, SCL‐90‐R and Fathers	Self‐report Quantitative and Cross‐sectional	Fathers of the four groups (AN, BN, EDNOS, HC)	No significant differences were found on any subscales or global indices of the SCL‐90‐R^h^ among the four father groups (*p* > 0.05)
Gómez‐Castillo et al. (2018)	To simultaneously explore the differences in parents' and their offspring's ED symptoms	30 and 19.5 (5.12)	F + M (29F, 1 M)	NR Mostly middle family SES (36.7%)	SCID‐I for the DSM‐IV	24 and 49.4 (6.6)	NR Mostly middle family SES (36.7%)	348 AN: 30, 30 M, 28F BN: 30, 29 M, 24F EDNOS: 30, 30 M, 30F HC^b^: 30, 29 M, 28F	Offspring, mothers, fathers	A demographic questionnaire, EDI‐2 and Fathers	Self‐report Quantitative and Cross‐sectional	Fathers of the four groups (AN, BN, EDNOS, HC)	No significant differences were found among the four father groups on the subscales of the EDI‐2^h^ (except for the Impulse Regulation subscale) (*p* = ns)
												Fathers of the BN group versus Fathers of the HC and AN group	Fathers of the BN group had significantly higher scores on the Impulse Regulation subscale of the EDI‐2 than both those of the HC (*p* = 0.019) and AN (*p* = 0.022) groups
Mark (1993)^a^	To examine separation‐individuation, autonomy, social support, family functioning, and impulse control difficulties (i.e., personal and parental problem drinking) in the groups	51 and 20.2 (3.09)	F	NR	SCID‐NP for the DSM‐III‐R	—	—	152 BN: 51 PC (bulimic panic disorder): 17 PC (panic disorder): 27 HC: 57	Offspring	Demographic information, A modified version of the SMAST and Offspring	Self‐report Quantitative and Cross‐sectional	The BN group versus The HC or panic disorder groups	No significant differences were found between the BN group versus HC (*p* = 0.34) or the panic disorder (*p* = 0.55) groups in terms of perceived paternal alcohol use
												The BN group versus The bulimic‐panic disorder group	No significant differences were found between the BN group versus the bulimic panic disorder group in terms of perceived paternal alcohol use (*p* = 0.25)
Pisetsky et al. (2017)^a^	To identify the prevalence of reported suicide attempts in the first‐ and second‐degree family members of individuals with an ED	836 and NR The mean age of the total sample: 26.1 (7.7)	NR The total ED sample included both females and males	NR	EDQ administered to participants recruited from the clinical setting	—	—	1870 AN: 274 BN: 836 BED: 208 EDNOS: 498 Purging disorder: 54	Offspring	Demographic information, EDQ and Offspring	Self‐report Quantitative and Cross‐sectional	The five ED groups (AN, BN, BED, EDNOS, Purging disorder)	The prevalence of suicide attempts by fathers was higher in the BN group than in the other ED groups. However, the model predicting offspring paternal suicide attempt from the ED diagnosis was not significant (*p* = 0.58), when controlling for participants' age and gender, meaning that the rates of the paternal suicide attempt did not significantly differentiate among the five offspring ED groups
Reto (1997)^a^	To explore the relationships among childhood maltreatment, dissociation, and bulimia symptomatology	23 and 30.0 (8.0)	F	Mostly Caucasian NR	Clinical interview based on the EDE	—	—	130 BN: 23 Non‐BN CG^d^: 107	Offspring	Demographic information, A questionnaire about family psychiatric and ED‐related demographics and Offspring	Self‐report Quantitative and Cross‐sectional	The BN group versus The non‐BN CG	No significant differences were found in the rates of paternal substance abuse (i.e., drug or alcohol), depression, suicide and general mental illness between the offspring groups (*p* = ns)
Schmidt, Tiller, and Treasure (1993)^a^	To examine whether the childhood family experiences differ by the ED diagnosis and affect the course of the illness	116 and BN/HistAN 23.6 (5.3) BN/noHistAN 24.4 (5.4)	F	NR Mostly non‐working class	Clinical assessment based on the DSM‐III‐R criteria and the nosologies of Russell (1985) and Fairburn (1986)	—	—	203 AN‐R: 64 AN‐BP: 23 BN/HistAN: 37 BN/noHistAN: 79	Offspring	Demographic information, A semi‐structured interview questions about childhood including parental mental illness and Offspring	Semi‐structured Interview Quantitative and Cross‐sectional	The four ED groups (AN‐R, AN‐BP, BN/HistAN, BN/noHistAN)	The percentages of mental illness occurrence in fathers did not significantly differ across the offspring ED groups (*p* = 0.70); 26% of the AN‐BP group, 21% of the BN/noHistAN group, 19% of the AN‐R group, and 14% of the BN/HistAN group reported paternal mental illness.
												The recent onset BN group versus The chronic BN group	Regarding the differences between the recent onset (≤ 4 years duration) and chronic BN (> 4 years duration) groups, paternal mental illness was more common in the chronic BN sample than in the recent onset group. However, this difference was not statistically significant (*p* = ns)
Tafà et al. (2017)	To explore whether families with adolescents with EDs have particular family functioning characteristics To evaluate adolescents' psychological profiles and their parents' psychopathological risk profiles, and then compare these profiles among the ED groups To examine how adolescents' psychological profile and parents' psychopathological risk scores affect the perception of adolescents' family functioning within each group	50 and NR The mean age range of the total sample: 14–17 (2.4)	F	Mostly Caucasian NR Mostly middle family SES	Clinical interview based on the DSM‐5 criteria	50 and NR	Mostly Caucasian NR Mostly middle family SES	150 AN: 50, 50 M, 50F BN: 50, 50 M, 50F BED: 50, 50 M, 50F	Offspring, mothers, fathers	Demographic information, SCL‐90‐R and Fathers	Self‐report Quantitative and Cross‐sectional	Fathers of the three groups (AN, BN, BED)	No significant differences were found among the three father groups on the subscales of the SCL‐90‐R^h^ (*p* = ns), except for the paranoid ideation and psychoticism ones (*p* < 0.05)
												Fathers of the BN group versus Fathers of the AN group	Fathers of the BN group tended to report significantly less paranoid ideation (*p* < 0.05) and psychoticism (*p* < 0.05) than did those of the AN group
													Fathers of adolescents with BN had psychopathology scores that exceeded the clinical cut‐off in the subscales of obsessive‐compulsive, anxiety, and phobic anxiety.
Woodside et al. (1998)^a^	To assess whether the relevant data demonstrates familial of eating disorders To examine whether there is evidence to support a tendency for ED subgroups to cluster together in families	36 and NR	F	NR	SCID for the DSM‐III‐R	—	—	2218 AN: 57 BN: 36	Offspring	Demographic information, A semi‐structured interview questions about family history and Offspring	Semi‐structured Interview Quantitative and Cross‐sectional	Paternal versus Maternal descent in the BN versus the AN group	When examining the rates of EDs by maternal and paternal descent in first‐ and second‐degree relatives, the rates of AN and BN by maternal descent were 3.9% and 3.7%, respectively versus 3.5% and 3.6% for paternal descent (*p* = ns), meaning that there was no evidence from the analysis of maternal versus paternal descent to support X‐linked dominant transmission in the EDs.
Subtheme 3: Other psychological variables													
Dyrenforth (1990)^a^	To explore the participants' attributions of sex role characteristics toward themselves and their parents	39 and 22.92 (5.61)	F	NR	Clinical assessment based on the DSM‐III‐R	—	—	475 Restricting BN: 39 Normal weight BN: 39 Weight‐obsessed: 44 Non‐ED CG^d^: 353	Offspring	Demographic information, BSRI and Offspring	Self‐report Quantitative and Cross‐sectional	The four groups (Restricting BN, Normal weight BN, Weight‐obsessed, Non‐ED CG)	The paternal masculinity (*p* = 0.29) or femininity (*p* = 0.31) scores reported by the offspring did not show significant differences among the groups
													No significant differences were found in the distribution of fathers' attributed BSRI dimensions^h^ among the offspring groups (*p* = 0.75)
Humphrey (1988)	To compare the relationship patterns within families of the ED and control groups	16 and 17.1 (1.7)	F	NR^e^	The semi‐structured clinical interview based on the DSM‐III criteria	16 and NR	NR^e^	222 AN‐R: 16, 16 M, 16F AN‐BP: 18, 18 M, 18F BN: 16, 16 M, 16F HC^b^: 24, 24 M, 24F	Offspring, mothers, fathers	Demographic information, The 1974 version of Benjamin's SASB rating scales of parents and self and Fathers	Self‐report Quantitative and Cross‐sectional	Fathers of the four groups (AN‐R, AN‐BP, BN, HC)	No significant differences were found among the four father groups on the cluster 1, 2, 5, 6, 7, 8 of the SASB ratings^h^ assessing their relations with themselves (introjection), when conducting the multiple comparisons test (*p* = ns)
													Significant differences were found for fathers on the cluster 3 (*p* < 0.01) and 4 (*p* < 0.05) ratings, although only one of them was driven by the difference between fathers of the BN group and those of another group (the HC group)
												Fathers of the BN group versus Fathers of the HC group	Fathers of the BN group reported themselves as less self‐protecting and enhancing (the cluster 4) than did their counterparts in the HC group (*p* = ns)
Kanakis and Thelen (1995)	To investigate whether offspring and parent variables were related to BN	36 and NR The mean age of the total proband sample: 18.9 (2.48)	F	NR^e^	BULIT‐R and The diagnostic interview ratings based on the DSM‐III‐R criteria	29 and NR The mean age of the total father sample: 47.5 (5.5)	NR	312 BN: 36, 34 M, 29F S‐BN: 39, 37 M, 34F Non‐BN CG^d^: 37, 32 M, 34F	Offspring, mothers, fathers	Demographic information, Tennessee Self‐Concept Scale, Body Image Scale, Body Esteem Scale, The Restraint and Disinhibition subscales of Three‐Factor Eating Questionnaire, History of Eating Questionnaire for parents and Fathers	Self‐report Quantitative and Cross‐sectional	Fathers of the three groups (BN, S‐BN, Non‐BN CG)	The three father groups did not show significant differences in terms of the self‐esteem scores (*p* = ns), when controlling for father's BMI
Ratti (1994)	To compare the nature of the intra‐ and interpersonal‐relationships in families of female adolescents with an internalizing and externalizing disorder	14 and 18.2 (1.8)	F	Caucasian NR Mostly middle family SES	Clinical interview based on the SADS‐C and the DSM‐III‐R	14 and NR	Caucasian NR Mostly middle family SES	132 BN: 14, 14 M, 14F PC (polydrug dependence): 13, 13 M, 13F HC: 17, 17 M, 17F	Offspring, mothers, fathers	Demographic information, SASB ratings and Fathers	Self‐report Quantitative and Cross‐sectional	Fathers of the three groups (BN, PC, HC)	No significant differences were found among the three father groups on the clusters of the SASB^h^ introjection ratings (*p* = ns)

Abbreviations: AN: anorexia nervosa; AN‐BP: anorexia nervosa—binging purging subtype; AN‐R: anorexia nervosa—restricting subtype; BDI: beck depression inventory; BED: binge‐eating disorder; BMI: body mass index; BN/HistAN: bulimia nervosa with a history of anorexia nervosa; BN/noHistAN: bulimia nervosa without a history of anorexia nervosa; BN: bulimia nervosa; BSRI: bem sex role inventory; BULIT‐R: the bulimia test—revised version; DSM‐5: diagnostic and statistical manual of mental disorders, the fifth edition; DSM‐III: diagnostic and statistical manual of mental disorders, the third edition; DSM‐III‐R: diagnostic and statistical manual of mental disorders, the third edition, revised version; DSM‐IV: diagnostic and statistical manual of mental disorders, the fourth edition; ED: eating disorder; EDE: eating disorder examination; EDI: eating disorders inventory; EDI‐2: eating disorders inventory‐2; EDNOS: eating disorder not Otherwise specified; EDQ: the eating disorders questionnaire; F: father; F: female; HC: control group without a psychiatric diagnosis or problem; M: male; M: mother; *N*: number; Non‐BN CG: control group assessed for only BN symptomatology; Non‐ED CG: control group assessed for only ED symptoms; NR: not reported; PC: control group with a psychiatric diagnosis or problem; PDQ‐R: the personality disorder questionnaire‐revised version; SADS‐C: the schedule for affective disorders and schizophrenia‐current version; SAS: the self‐rating anxiety scale; SASB: structural analysis of social behavior; S‐BN: subclinical bulimia nervosa; SCID for the DSM‐III‐R: the structured clinical interview for the DSM‐III‐R; SCID‐I for the DSM‐III‐R/the DSM‐IV/the DSM‐IV‐TR: the structured clinical interview for the DSM‐III‐R/the DSM‐IV/the DSM‐IV‐TR axis‐I disorders; SCID‐NP for the DSM‐III‐R: structured clinical interview for the DSM‐III‐R—nonpatient version; SCL‐90‐R: The symptom check list‐revised version; SD: standard deviation; SES: socioeconomic status; SMAST: Short Michigan Alcoholism Screening Test; TAS‐20: Toronto Alexithymia Scale‐20; TCI: temperament character inventory.

^a^ Data collection tools regarding the father‐specific psychosocial variable were rated by the offspring or clinician in this study.

^b^ The participants in the control group were assessed in terms of psychiatric conditions, addictions, or intellectual disability; however, the screening criteria for BN were unclear for these participants.

^c^ This study reported the father's and family's SES as mostly Class 4 on a 5‐point Likert scale, but the author did not specify what “Class 4” means.

^d^ The participants in the control group were assessed for BN or an ED symptomatology; however, no information was provided whether these participants had other clinical conditions.

^e^ The offspring groups did not differ in their socioeconomic status and/or family income; however, no information was provided on the socioeconomic levels corresponding to the overall mean value of the relevant analysis.

^f^ Only data collection tools used for the demographic and clinical data and the father‐specific variables are reported.

^g^ ns: non‐significant (i.e., There is no the exact p‐value information in the study).

^h^ The subscales of the questionnaires: BSRI: Masculine, Feminine, Androgynous, Undifferentiated; EDI: Drive for Thinness, Bulimia, Body Dissatisfaction, Inefficiency, Perfectionism, Interpersonal Distrust, Lack of Self‐awareness, Fears of Maturity, Asceticism, Impulse Control, Lack of Confidence in Company; EDI‐2: Drive for Thinness, Bulimia, Body Dissatisfaction, Ineffectiveness, Perfectionism, Interpersonal Distrust, Interoceptive Awareness, Maturity Fears, Asceticism, Impulse Regulation, Social Insecurity; SASB: Self‐spontaneous, Self‐accepting and Exploring, Self‐nourishing and Cherishing, Self‐monitoring and Restraining, Self‐indicting and Oppressing, Self‐rejecting and Destroying, Day‐dreaming and Neglecting of self, Self‐protecting and Enhancing; SCL‐90‐R: Somatisation, Obsession‐Compulsion, Interpersonal Sensitivity, Depression, Anxiety, Hostility, Phobic Anxiety, Paranoid Ideation, Psychoticism, Global Severity Index, Positive Symptom Distress Index; TAS‐20: F1: Difficulty in identifying feelings, F2: Difficulty in describing feelings to others, F3: Externally orientated thinking; TCI: (i) Temperamental Traits: Novelty Seeking, Harm Avoidance, Reward Dependence, Persistence, (ii) Character Traits: Self‐directedness, Cooperativeness, Self‐Transcendence.

**TABLE 3:** The eligible studies with father‐specific body‐related and psychological variables—associations with outcomes in offspring.

Author(s) and year	The aim(s) of study	*N* and mean age (SD) (offspring with BN)	Sex	Race and ethnicity and SES	Diagnostic procedure	*N* and mean age (SD) (father)	Race and ethnicity and SES	Total *N*	Studied family member(s)	Data collection tools^c^ and reported by	Research method and study design	Main findings^d^, ^e^
Subtheme 1: Father's body‐image and related factors												
Benninghoven et al. (2007)	To assess the body image perception of the participants with EDs and their fathers	15 and 20.1 (3.1)	F	NR	SCID‐I for the DSM‐IV	15 and 50.2 (6.8)	NR	84 AN: 27, 27F BN:15, 15F	Offspring, fathers	Demographic information, BIA, The somatomorphic matrix, Perceptual body size distortion and self‐ideal discrepancy calculations and Fathers	Self‐report Quantitative and Cross‐sectional	BN daughters' self‐ideal discrepancy was significantly negatively associated with their fathers' perceptual body size distortion (only muscularity dimension) (*p* = 0.007) and self‐ideal discrepancy (only muscularity dimension) (*p* = 0.05)
												No significant associations were found between BN daughters' perceptual body size distortion and fathers' perceptual body size distortion or self‐ideal discrepancy in terms of body‐fat and muscularity dimensions (*p* = ns)
Subtheme 2: Father's eating‐ and weight‐related behaviors												
Fernández‐Aranda et al. (2007)^a^	To develop a sensitive instrument to the environmental factors related to the development of eating disorders To assess this instrument as a tool to measure associated factors in ED groups To examine the relationship between individual and family eating patterns or food choices during early life and the likelihood of developing a subsequent ED	123 and NR The mean age of the total clinical sample: 24.8 (5.6)	NR The total ED sample included both females and males	NR	SCID‐I for the DSM‐IV	—	—	421 AN: 88 BN: 123 EDNOS: 50 HC: 160	Offspring	Demographic information, The Early Eating Environmental subscale of the CCQ and Offspring	Self‐report Quantitative and Case–control	In terms of the effects of family eating patterns during early life on the eating disorder later, the importance placed on food by fathers was significantly associated with the likelihood for the subsequent BN in the offspring, after adjusting for sex and age (OR = 2.30; *p* = 0.008)
												Fathers' attention to healthy eating was likely to be positively correlated with the occurrence of BN, after adjusting for sex and age (*p* = 0.081)
												In terms of the effects of family eating patterns during early life on the BMI later, the offspring's current BMI was not significantly related to any father‐related food style variable in the BN or HC groups, after adjusting for sex and age (*p* = ns)
Subtheme 3: Father's personality characteristics												
Fassino, Amianto, and Abbate‐Daga (2009)	To compare the personality traits and eating psychopathology of patients with EDs and their parents' personality characteristics with those of the control participants To explore the associations between parents' personality characteristics and their daughters' personality traits and eating psychopathology	37 and 25.59 (7.6)	F	NR	SCID‐I for the DSM‐III‐R	31 and 63.91 (2.5)	NR	447 AN‐R: 38, 32 M, 28F AN‐BP: 30, 29 M, 28F BN: 37, 36 M, 31F HC: 54, 54 M, 50F	Offspring, mothers, fathers	Demographic information, TCI, EDI‐2 and Fathers	Self‐report Quantitative and Cross‐sectional	Significant correlations between the paternal TCI^e^ scores and the EDI‐2^e^ scores of the offspring with BN: Fathers' Reward Dependence (TCI) negatively predicted their offspring's EDI‐2‐Drive for Thinness (*p* < 0.027)
												Fathers' Persistence (TCI) positively predicted their offspring's EDI‐2‐Maturity Fears (*p* < 0.005)
												Fathers' Self‐directedness (TCI) negatively predicted their offspring's EDI‐2‐Maturity Fears (*p* < 0.002)
Pourdehghan et al. (2024)	To explore the association of parental personality disorders with offspring EDs	56 and NR The age range of the ED cohort: 6–18	NR The total ED sample included both boys and girls	NR Iranian	K‐SADS‐PL	37 and NR The mean age of the father sample: 43.81 (10.61)	NR Iranian	350 AN: 38, 36 M, 25F BN: 56, 48 M, 37F BED: 46, 45 M, 19F Cohort Population (children and adolescence): 27,111 Cohort Population (mothers and fathers): 45,309	Offspring, mothers, fathers	Demographic information, MCMI‐III and Fathers	Self‐report Quantitative and Cross‐sectional	The adjusted results of the relevant analysis assessing the relationships of paternal Personality Disorders (PDs) with the offspring's ED diagnosis indicated that the most paternal PDs included histrionic and obsessive‐compulsive PDs by 22.1% and 18.6%, respectively, in the total father sample. However, paternal PDs (paranoid, schizotypal, antisocial, avoidant, sadistic, borderline, schizoid, dependent, masochistic, negativistic, narcissistic, melancholic, obsessive‐compulsive, histrionic) was not significantly associated with the offspring ED diagnoses (including BN) (*p* > 0.05)
Subtheme 4: Father's psychopathological profile												
Arikian et al. (2008)^a^	To assess parental variables as predictors of treatment response and long‐term outcome in patients with BN	94 and 24.4 (5.4) (baseline) 34.5 (5.3) (follow‐up)	F	Mostly Caucasian NR	Clinical assessment based on the DSM‐III criteria (baseline) The SCID‐I for the DSM‐IV (follow‐up)	—	—	94 BN: 94	Offspring	Demographic information, PDBQ and Offspring	Self‐report Quantitative and Longitudinal	At treatment end, the significant association was found between the poor BN treatment outcome and paternal substance abuse. Accordingly, participants with the poor outcome were significantly more likely to have reported fathers who have substance abuse (including alcohol use) than those with the good outcome (*p* < 0.018)
												At long‐term follow‐up, no significant associations were found between patients' poor or good BN treatment outcomes and their fathers' ED, severe depression, severe anxiety, severe psychopathology, substance abuse, or any mental health problem (*p* > 0.05)
Espina, de Alda, and Ortego (2003)	To assess the dyadic adjustment in parents of patients with EDs by comparing the study outcomes with the control participants	31 and NR The selection criterion for age: 15–25	F	NR	Clinical interview based on the DSM‐IV criteria	31 and NR The mean age of the total father sample: 51.1 (5.8)	NR	441 AN‐R: 20, 20 M, 20F AN‐BP: 23, 23 M, 23F BN: 31, 31 M, 31F PC: 32, 32 M, 32F HC^b^: 41, 41 M, 41F	Offspring, mothers, fathers	Demographic information, BDI, SAS and Fathers	Self‐report Quantitative and Cross‐sectional	In a logistic regression with the offspring ED diagnosis as the outcome and the BDI and SAS scores of fathers as predictors, no variable predicted assignment to the BN group (*p* > 0.05)

Abbreviations: AN: anorexia nervosa; AN‐BP: anorexia nervosa—binging purging subtype; AN‐R: anorexia nervosa—restricting subtype; BDI: beck depression inventory; BED: binge‐eating disorder; BIA: bio‐impedance analysis; BMI: body mass index; BN: bulimia nervosa; CCQ: the cross‐cultural (environmental) questionnaire; DSM‐III: diagnostic and statistical manual of mental disorders, the third edition; DSM‐IV: diagnostic and statistical manual of mental disorders, the fourth edition; ED: eating disorder; EDI‐2: eating disorders inventory‐2; EDNOS: eating disorders not otherwise specified; F: father; F: female; HC: control group without a psychiatric diagnosis or problem; K‐SADS‐PL: kiddie schedule for affective disorders and schizophrenia—present and lifetime version; M: mother; MCMI‐III: millon clinical multiaxial inventory‐the third edition; *N*: number; NR: not reported; OR: odds ratio; PC: control group with a psychiatric diagnosis or problem; PD: personality disorder; PDBQ: A Psychiatric Data Base Questionnaire; SAS: The Self‐Rating Anxiety Scale; SCID‐I for the DSM‐III‐R/the DSM‐IV: the structured clinical interview for the DSM‐III‐R/the DSM‐IV axis‐I disorders; SD: standard deviation; SES: socioeconomic status; TCI: temperament character inventory.

^a^ Data collection tools regarding the father‐specific psychosocial variable were rated by the offspring in this study.

^b^ The participants in the control group were assessed in terms of general mental health; however, the screening criteria for BN were unclear for these participants.

^c^ Only data collection tools used for the demographic and clinical data and the father‐specific variables are reported.

^d^ ns: non‐significant (i.e., There is no the exact p‐value information in the study).

^e^ The subscales of the questionnaires: EDI‐2: Drive for Thinness, Bulimia, Body Dissatisfaction, Ineffectiveness, Perfectionism, Interpersonal Distrust, Interoceptive Awareness, Maturity Fears, Asceticism, Impulse Regulation, Social Insecurity; TCI: (i) Temperamental Traits: Novelty Seeking, Harm Avoidance, Reward Dependence, Persistence, (ii) Character Traits: Self‐directedness, Cooperativeness, Self‐Transcendence.

### 3.4 Study Quality Assessment

The JBI (The Joanna Briggs Institute, n.d.) checklists for the cross‐sectional (*n* = 27, ~93%), case–control (*n* = 1, 3.5%) (Fernández‐Aranda et al. 2007), and cohort study (*n* = 1, 3.5%) (Arikian et al. 2008) designs were used to appraise the methodological quality of the included 29 studies. 25 (86%) out of the eligible studies had a low or moderate risk of bias based on the frequency of responses with “no” or “unclear” (below 50%); only four studies (14%) showed a high risk of bias trend, whereby 50% of items were rated as “unclear” (Bonal 1990; Bonne et al. 2003; Humphrey 1988; Pentz 1992).

Regarding the included cross‐sectional studies (*n* = 27, ~93%), detailed information about study setting was present in all of them, whereas sociodemographic characteristics of participants were not clearly reported in the majority of the studies (*n* = 25, ~93%). Additionally, eight studies (~30%) did not include clearly defined inclusion criteria for the study sample. In four studies (~15%), the assessment criteria for the presence or absence of BN were unclear for control groups (Bonne et al. 2003; Espina, de Alda, and Ortego 2003; Gómez‐Castillo et al. 2018; Humphrey 1988), and the procedure for establishing the presence of BN was discrepant between the BN and comparison groups in three studies (11%) (Dyrenforth 1990; Elliott‐Harper 1990; Espina 2003). The validity and reliability of the measurement tools for the relevant paternal and/or offspring variables were unclear in a total of 15 studies (55%). Furthermore, the control of possible confounding factors was unclear in 17 cross‐sectional studies (63%). In one article with a case–control study design (3.5%) (Fernández‐Aranda et al. 2007), the same identification procedure was not applied to the case and control groups, and they were not matched appropriately. Finally, in the one longitudinal study (Arikian et al. 2008), insufficient information was provided about the reliability and validity of the measurement tool for the paternal variable of interest and the details of the follow‐up process. For more detailed information, see Data S2.

### 3.5 Study Findings

#### 3.5.1 Findings From Studies Reporting Group Comparisons in Fathers

##### 3.5.1.1 Body‐Related Variables

This main theme encompassed two subthemes: father's body‐image and related factors and father's eating‐ and weight‐related behaviors (Table 1).

###### 3.5.1.1.1 Father's Body‐Image and Related Factors

Six studies (~21%) investigated the body‐image perceptions and attitudes toward being overweight of fathers of participants with BN (Benninghoven et al. 2007; Bonal 1990; Bonne et al. 2003; Gómez‐Castillo et al. 2018; Kanakis and Thelen 1995; Moreno and Thelen 1993).

Kanakis and Thelen (1995) found no significant differences in fathers' self‐ideal perceptions and body importance among the fathers of BN, S‐BN, and non‐BN control groups. Another study aiming to understand fathers' attitudes toward themselves about being overweight showed no difference in attitudes on the Family History of Eating‐Parent scale (FHE‐P) among the fathers of the BN, S‐BN, and non‐BN control groups (Moreno and Thelen 1993). Similarly, Bonal (1990) did not report significant differences among the BN, PC, and HC groups regarding the perceived paternal concern about the fathers' own weight, which was rated by the offspring rather than the fathers themselves. Additionally, two studies (9%) using the Eating Disorder Inventory (EDI and EDI‐2) found no significant differences between fathers of the BN and HC groups on Drive for Thinness and Body Dissatisfaction subscales (Bonne et al. 2003; Gómez‐Castillo et al. 2018). Overall, studies revealed no remarkable differences in body‐image related variables between fathers of people with BN and HCs.

Moreover, another study found no significant differences between fathers of people with BN and AN (Benninghoven et al. 2007), in fathers' perceptual body size distortion and self‐ideal discrepancy ratings in terms of both body‐fat and muscularity, meaning that fathers of both the BN and AN group wished to be thinner and more muscular. However, there was no control group. Finally, in the study comparing fathers of individuals with BN to AN and EDNOS (Gómez‐Castillo et al. 2018), the Drive for Thinness and Body Dissatisfaction subscale scores of the EDI‐2 did not differ significantly between fathers of the BN and AN groups, but fathers of offspring with BN reported higher body dissatisfaction than their counterparts of the EDNOS group.

###### 3.5.1.1.2 Father's Eating‐ and Weight‐Related Behaviors

Six studies (~21%) focused on paternal eating‐ and weight‐related factors (Bonal 1990; Kanakis and Thelen 1995; Mangweth, Pope, and Hudson 1995; Moreno and Thelen 1993; Pentz 1992; Reto 1997); in four of them (~67%), these variables were rated by the offspring, not the fathers themselves (Bonal 1990; Mangweth, Pope, and Hudson 1995; Pentz 1992; Reto 1997).

The studies rated by the fathers themselves (33%) did not reveal significant differences among the BN, S‐BN, and non‐BN control groups (Kanakis and Thelen 1995; Moreno and Thelen 1993), in the Three‐Factor Eating Questionnaire and History of Eating Questionnaire for Parents (eating behavior and dieting scores) (Kanakis and Thelen 1995), or in the FHE‐P (attitudes toward their own dieting) (Moreno and Thelen 1993).

However, offspring with BN in the USA reported in the semi‐structured individual interview about preoccupation with weight of family members that their fathers were significantly more likely to diet than their BN counterparts in Austria (Mangweth, Pope, and Hudson 1995). Similarly, in another study (Reto 1997), fathers were reported to be significantly more likely to be on chronic dieting in the BN group than in the non‐BN CG. On the other hand, in two studies (33%) examining the familial attitudes and behaviors regarding weight and dieting through unstandardized self‐report questionnaires by the offspring, there were no significant differences in the rates of paternal dieting behaviors between the BN, PC, and HC groups (Bonal 1990; Pentz 1992).

Overall, the majority of studies did not find significant differences in dieting behaviors between fathers of people with BN and comparator groups, although this may be amenable by cultural context (Mangweth, Pope, and Hudson 1995) and the individual reporting the data (i.e., the offspring or father) (Reto 1997).

##### 3.5.1.2 Psychological Variables

This main theme was grouped into three subthemes: father's personality characteristics, father's psychopathological profile, and other psychological variables (i.e., father's relationship with himself and self‐esteem; Table 2).

###### 3.5.1.2.1 Father's Personality Characteristics

Three studies (10%) explored father's personality characteristics using the same self‐report questionnaire (Temperament and Character Inventory—TCI) (Amianto et al. 2015; Fassino et al. 2003; Fassino, Amianto, and Abbate‐Daga 2009). In one study (3.5%), fathers' TCI traits were divided into clusters of “Explosive/Methodological”, “Independent/Methodological”, “Immature”, “Asocial”, and “Mature”, which did not differentiate among the ED diagnoses of their offspring (i.e., BN, AN, and EDNOS) (Amianto et al. 2015). On the other hand, fathers of the BN group reported lower “Persistence” (i.e., perseverance in spite of fatigue and frustration) on the TCI than those of the HCs (Fassino et al. 2003; Fassino, Amianto, and Abbate‐Daga 2009). However, no significant differences were found on the other temperament (i.e., Novelty Seeking, Harm Avoidance, Reward Dependence) and character dimensions (i.e., Self‐directedness, Cooperativeness, Self‐transcendence) of the TCI between the fathers of the BN and HC groups (Fassino et al. 2003) or the AN‐R, AN‐BP, and HC groups (Fassino, Amianto, and Abbate‐Daga 2009). Personality characteristics recorded by the TCI did not appear to significantly differ between fathers of people with BN and HCs, and between fathers of those with BN and AN or EDNOS.

Furthermore, Espina, de Alda, and Ortego (2003) addressed alexithymia in fathers of patients with EDs compared with those of the controls using a self‐report questionnaire (Toronto Alexithymia Scale‐20; TAS‐20), finding no differences between fathers of the BN group and those of the other groups (i.e., HC, AN‐R, AN‐BP), even when controlling for paternal anxiety and depression symptoms. Additionally, using the Personality Disorder Questionnaire (PDQ‐R), Carney, Yates, and Cizadlo (1990) found no significant differences in the total score between fathers of the BN and control groups. However, fathers of the BN group had significantly approximately three times higher pathological score on the Histrionic subscale than their counterparts in the HC group.

###### 3.5.1.2.2 Father's Psychopathological Profile

This subtheme was the most examined subject among studies, with 11 studies (~38%) (Figure 1). Fathers' psychopathological variables were rated by their offspring in five (~45%) out of 11 studies (Mark 1993; Pisetsky et al. 2017; Reto 1997; Schmidt, Tiller, and Treasure 1993; Woodside et al. 1998), by fathers themselves in four studies (~36%) (Bonne et al. 2003; Gómez‐Castillo et al. 2017, 2018; Tafà et al. 2017), and by the offspring's therapist in one study (9%) (Birckhead 1990). However, the person (i.e., offspring or father) reporting the paternal variable was not clearly stated in one study (9%) (Elliott‐Harper 1990).

Two studies (~7%) investigated paternal alcohol/substance abuse (Mark 1993; Reto 1997). The first study (Reto 1997) found no significant differences in the proportions of paternal substance use (i.e., drug or alcohol) as rated by the offspring in the BN group and non‐BN CG. Another study examined perceived paternal alcohol use utilizing a modified version of the Short Michigan Alcoholism Screening Test (SMAST), with no differences between the BN group versus the PC (i.e., panic disorder, bulimic panic disorder) or HC groups (Mark 1993).

Three studies (10%) examined ED psychopathology in fathers (Bonne et al. 2003; Gómez‐Castillo et al. 2018; Woodside et al. 1998); one of them were based on the offspring's reports (Woodside et al. 1998). In the study by Woodside et al. (1998), paternal versus maternal descent of EDs did not significantly differentiate the BN from the AN group (Woodside et al. 1998). The other studies based on fathers' reports used the EDI and EDI‐2 scales to explore paternal ED‐related symptoms (Bonne et al. 2003; Gómez‐Castillo et al. 2018). Bonne et al. (2003) did not find significant differences on any subscale of the EDI between fathers of the BN and HC groups, whereas Gómez‐Castillo et al. (2018) reported significant difference between the fathers of the BN and other groups on the Impulse Regulation subscale. Fathers of the BN group had higher scores on this dimension than those of HCs and the AN group.

Regarding suicidality, in one study (3.5%), participants with BN nominally reported more paternal suicide attempts compared to the AN, BED, EDNOS, and Purging Disorder groups (Pisetsky et al. 2017), although the prevalence of paternal suicide attempt did not significantly differentiate between the ED diagnoses in offspring, when controlling for participants' age and gender. Similarly, another study (3.5%) (Reto 1997) reported that the rate of paternal suicide attempt in the BN group was not significantly different from that in the non‐BN CG.

Schmidt, Tiller, and Treasure (1993) reported no differences in paternal mental illness between the ED groups (i.e., AN‐R, AN‐BP, BN without a history of AN, and BN with a history of AN), although paternal mental illness was nominally, but not significantly, more common in chronic BN than in recent‐onset BN. Additionally, two studies (9%) assessed fathers' broad psychiatric symptoms using the Symptom Checklist‐90 (SCL‐90‐R), with one finding no differences between fathers of the BN, AN, EDNOS, and HC groups (Gómez‐Castillo et al. 2017) and another finding lower scores in paranoid ideation and psychoticism in the fathers of participants with BN compared to those of the AN group (Tafà et al. 2017). Reto (1997) also did not report significant difference in the presence of paternal (general) mental illness between the BN group and non‐BN CG.

Two studies (~7%) addressed paternal anxiety and depression based on reports of their offspring (Reto 1997) and their therapists (Birckhead 1990). Both studies did not reveal differences among ED groups (i.e., BN, AN‐R, AN‐BP) (Birckhead 1990) or between the participants with BN and non‐BN CG (Reto 1997) in terms of paternal anxiety or depression on unstandardized measurements. Elliott‐Harper (1984) utilized a structured interview to collect the data about participants' family psychiatric history. The rates of fathers with “other psychiatric pathology” were not significantly different among the offspring groups (i.e., BN, AN, non‐ED CG).

Concluding from these studies, there is broadly no evidence for an elevated incidence of mental illness, including substance/alcohol abuse, ED psychopathology, suicidality, anxiety, and depression, in fathers of people with BN compared with those of the control and other ED groups.

###### 3.5.1.2.3 Other Psychological Variables

This subtheme included four studies (~14%) that examined fathers' introjection and self‐esteem as well as attributed paternal masculinity/femininity (Dyrenforth 1990; Humphrey 1988; Kanakis and Thelen 1995; Ratti 1994); one of them was based on the offspring's report (Dyrenforth 1990).

Two studies (~7%) focused on whether and how father groups differed on the clusters of the Structural Analysis of Social Behavior ratings (SASB) in terms of their introjection. In the study by Humphrey (1988), the only difference found was between fathers of people with BN and the HCs, whereby fathers rated themselves as less “protecting and enhancing” than did their counterparts in the control group. However, another study (Ratti 1994) did not reveal significant differences on any cluster of the SASB introjection ratings among fathers of the BN, PC (i.e., polydrug dependence), and HC groups.

Kanakis and Thelen (1995) assessed paternal self‐esteem comparing fathers of the BN, S‐BN, and non‐BN control groups. They found that there were no significant differences in fathers' self‐esteem among the groups.

Finally, one study (3.5%) using the Bem Sex Role Inventory (BSRI) explored the offspring's attributions of sex role characteristics toward their fathers (Dyrenforth 1990). Neither paternal masculinity nor paternal femininity scores differentiated among the restricting BN, normal weight BN, “weight‐obsessed”, and non‐ED control groups. The same study also reported no significant differences in the distribution of fathers' attributed BSRI dimensions (i.e., masculine, feminine, androgynous, undifferentiated) among the groups.

#### 3.5.2 Findings From Studies Reporting Associations Between Paternal and Offspring Variables

Six (~21%) out of 29 included studies reported the associations of fathers' body‐image, food attitudes, personality traits, depression and anxiety scores, and substance misuse with their offspring's BN and related outcomes (Arikian et al. 2008; Benninghoven et al. 2007; Espina, de Alda, and Ortego 2003; Fassino et al. 2003; Fernández‐Aranda et al. 2007; Pourdehghan et al. 2024). For more information, see Table 3.

Benninghoven et al. (2007) examined whether there were significant relationships between the perceptual body‐size distortion (i.e., perceived minus actual percentage body‐fat) or self‐ideal discrepancy (i.e., perceived minus desired percentage body‐fat) ratings of the daughters with BN and their fathers' perceptual body‐size distortion or self‐ideal discrepancy ratings in terms of both body‐fat and muscularity dimensions. The self‐ideal discrepancy, but not the body size distortion, of the offspring with BN was significantly associated with their fathers' perceptual body‐size distortion and self‐ideal discrepancy ratings (in the muscularity but not body‐fat dimensions).

In another study (Fernández‐Aranda et al. 2007), the BN group was compared with controls in terms of the associated paternal factors during early life with the later BN risk in the offspring. A greater value placed on food by fathers, but not the father's attention on healthy eating, was significantly related to the likelihood of subsequent BN in the offspring, after adjusting for sex and age. The current body mass index (BMI) of the offspring was not significantly associated with any father‐related food style variable during early life in the BN or HC groups, when adjusting for sex and age.

Pertaining to the relationship between paternal personality and ED psychopathology in the offspring, Fassino, Amianto, and Abbate‐Daga (2009) found that drive for thinness (EDI‐2) of the BN group were negatively predicted by their fathers' reward dependence, while the maturity fears (EDI‐2) of the BN group was positively predicted by their fathers' persistence, but negatively predicted by their fathers' self‐directedness. In contrast, no personality disorder in fathers was related to any ED diagnosis in their offspring in a study using the Millon Clinical Multiaxial Inventory‐III (MCMI‐III) (Pourdehghan et al. 2024).

Two studies (~7%) investigated the associations between paternal psychopathologies and the offspring's BN diagnosis and treatment outcome (Arikian et al. 2008; Espina, de Alda, and Ortego 2003). The study by Arikian et al. (2008) revealed that the patients with BN with a poor outcome (i.e., disordered eating defined as the presence of any binge‐eating/purging episodes in the 4 weeks preceding) at the end of treatment were significantly more likely to report their fathers as having had substance abuse than those with a good outcome (i.e., remission defined as the absence of binge‐eating/purging symptoms in the 4 weeks preceding). However, there were no significant relationships between paternal substance abuse and the poor or good treatment outcome of the offspring with BN at the 10‐year follow‐up time‐point. The same study also reported no significant associations between the patients' poor or good BN treatment outcomes and their fathers' ED, severe anxiety, depression, psychopathology, and any mental health problem at the 10‐year follow‐up. Similarly, Espina, de Alda, and Ortego (2003) did not find significant associations between paternal depression and anxiety scores and their offspring's BN diagnosis relative to the AN‐BP and control groups.

Overall, no significant relationships were found between paternal perceptual body size distortion and self‐ideal discrepancy ratings (both body‐fat and muscularity dimensions), attention on healthy eating in early life, several personality traits and/or disorders, and psychopathologies (ED, depression, anxiety, any mental health problem) and the offspring's perceptual body‐size distortion, the subsequent BN risk and/or current BMI, several ED features, BN diagnosis, and treatment outcomes (poor or good) at long‐term follow‐up. However, fathers' muscularity ratings (perceptual body‐size distortion and self‐ideal discrepancy), food attitudes (the importance placed on food during childhood), certain personality characteristics (reward dependence, persistence, self‐directedness), and substance abuse were found to be associated with the offspring's self‐ideal discrepancy, risk of developing BN later, some ED‐traits (drive for thinness, maturity fears), and poor outcome at treatment end.

## 4 Discussion

### 4.1 Summary of Evidence

This systematic review synthesized the results of 29 studies investigating psychosocial and psychopathological characteristics of fathers of individuals with BN and their possible associations with the offspring's BN and related factors.

Overall, fathers of the BN group did not discernibly differ from those of controls and clinical comparison groups (i.e., AN, subclinical BN, BED, EDNOS, Purging Disorder, and psychiatric control groups) in most body, eating, and weight‐related variables, including perceptual body‐size distortion and self‐ideal discrepancy (in body‐fat and muscularity), body importance, attitudes toward themselves being overweight, concern about their own weight, eating/dieting behaviors, and ED‐associated features (except for impulse regulation and body dissatisfaction). Similarly, no differences were found in other aspects of the general psychological functioning of fathers (i.e., personality characteristics, psychopathological profiles, suicide attempt, substance/alcohol abuse, several introjection dimensions, self‐esteem, and attributed sex roles) between the groups.

However, some studies found differences in impulse regulation, body dissatisfaction, histrionic traits, persistence, paranoid ideation, psychoticism, and tendencies to self‐protect and self‐enhance. Additionally, in some studies, fathers' food attitudes, muscularity ratings, personality traits, and substance misuse were found to be associated with their offspring's risk of developing BN, body dissatisfaction, ED traits, and poor outcome at treatment end. This evidence suggests possible links between certain paternal psychological and psychopathological characteristics and the offspring's BN symptomatology and ED‐related features. These findings are summarized in Table 4.

**TABLE 4:** Psychological features that were more pronounced in fathers of people with BN or associated with outcomes in offspring.

Psychological domain	Specific psychological features
Group comparisons in fathers	
Body‐image	Higher body dissatisfaction^a^
Personality characteristics	High levels of histrionic traits^b^ Lower persistence^b^
General psychological functioning	More difficulties with impulse regulation^b^, ^c^ Less paranoid ideation and psychoticism^c^ Low levels of self‐protecting and enhancing^b^
Associations with outcomes in offspring	
Body‐image	Father's perceptual body‐size distortion and self‐ideal muscularity ratings and the offspring's self‐ideal body rating
Eating‐related behaviors	The importance on food placed by the father during early life and the risk of developing BN later in the offspring
Personality characteristics	Father's reward dependence, persistence and self‐directedness and offspring's drive for thinness and maturity fears
General psychological functioning	Increased prevalence of paternal substance abuse and the offspring's poor outcome at treatment end

^a^ Fathers of the BN versus the EDNOS group.

^b^ Fathers of the BN versus the HC group.

^c^ Fathers of the BN versus the AN group.

The diversity in assessment measures, offspring's clinical profiles and sociodemographic characteristics and the low power of the studies may account for some discrepant findings. For example, although some studies used the same questionnaire (Gómez‐Castillo et al. 2017; Tafà et al. 2017), they recruited different offspring samples, obscuring comparison between the studies. Additionally, these studies all had a cross‐sectional research design, in which obtaining discrepant results regarding relationships between the variables in question is more common than in longitudinal studies (Phillips 2006). Thus, there is a need for longitudinal research with more homogenous research methods in order to facilitate more reliable conclusions.

Several studies compared paternal variables between the different ED diagnoses; some of them also explored the relationships between certain father‐specific factors and offspring's outcomes (Benninghoven et al. 2007; Gómez‐Castillo et al. 2018; Fassino, Amianto, and Abbate‐Daga 2009; Tafà et al. 2017). For example, in the study by Gómez‐Castillo et al. (2018), fathers' body dissatisfaction was higher in the BN group than those of the EDNOS group. Similarly, the difference between the perceived and desired body‐fat of the offspring was associated with their father's self‐ideal muscularity scores in the BN group, but their father's BMI in the AN group. These findings are interesting against the backdrop of previous literature about people with BN as the patients were reported as more dissatisfied with their bodies in comparison to the other ED groups (Cash and Deagle 1997; Laporta‐Herrero et al. 2018; Ruuska et al. 2005). The patients' dissatisfaction with their bodies might be linked to the body dissatisfaction experienced by their fathers. However, the mechanisms of whether and how body dissatisfaction of fathers is transferred to their offspring with BN cannot be elucidated by the studies included in this systematic review due to their research design (i.e., cross‐sectional).

Furthermore, the likelihood for the subsequent BN in the offspring was significantly associated with the importance placed on food, not healthy eating, by the father during childhood (Fernández‐Aranda et al. 2007). Although the findings of this single case–control study need to be corroborated by cohort studies, they appear intriguing in terms of investigating different aspects of paternal attitudes toward food as risk factors for the offspring's BN in future research. Also, these results are based on only the offspring's reporting. However, several studies revealed the differences between the reports of fathers and their offspring with BN on the same variable (not related to food) (Bonne et al. 2003; Tafà et al. 2017). Thus, quantitative and qualitative studies focusing on both fathers' relationship with food and how it is perceived by the offspring might help to understand the perceptual differences and the potential influences of paternal eating attitudes on the BN symptomatology in detail.

Interestingly, the fathers of the BN group had higher impulse regulation difficulties than those of the AN group (Gómez‐Castillo et al. 2018). One risk factor relating to the emergence of the binge‐eating/purging ED psychopathology is impulsivity (Claes, Vandereycken, and Vertommen 2005; Crisp and Grant 2024). Evidence has shown that higher levels of impulsivity is related to poorer BN treatment outcomes, and that decreased impulsivity preceded a decline in binge‐eating behavior (Fichter, Quadflieg, and Rief 1994; Giner‐Bartolomé et al. 2015; Keel and Mitchell 1997). Therefore, this finding of the present review highlights the need for more research examining the relationship between impulsivity in fathers and their offspring with BN. In addition, addressing paternal impulsivity in the treatment process may be helpful to support an offspring with BN. Given that children with BN described their relationships with their fathers as less nurturing and supportive, but more blaming and rejecting than those with AN (Fichter and Quadflieg 1996; Haslam et al. 2008; Herraiz‐Serrrano et al. 2015; Humphrey 1988), developing an understanding of such paternal behaviors and their perception by the offspring can play an important role in reorganizing their relationship dynamics, thus making valuable contributions to the treatment outcomes of BN.

### 4.2 Strengths and Limitations

To the authors' knowledge, the present study is the first systematic review to comprehensively examine psychosocial and psychopathological characteristics of fathers of individuals with BN and their possible associations with the offspring's BN‐related outcomes based on the available literature. This review included studies that were conducted with participants with clinically diagnosed BN, increasing the homogeneity of the included samples.

However, this review included only the quantitative studies. Qualitative research would add depth to these findings (Tenny, Brannan, and Brannan 2022). Furthermore, the majority of the included studies had a cross‐sectional research design. Only two studies (~7%) (case–control and longitudinal) explored the associations between paternal psychological factors and the risk of development of BN and the BN treatment outcome (Arikian et al. 2008; Fernández‐Aranda et al. 2007). As longitudinal studies are lacking, we cannot draw conclusions on whether the identified features of fathers are related to the risk of developing BN, its prognosis or treatment outcomes.

In addition, the variable of interest was reported by fathers in 16 studies (55%) and reported by offspring in 11 studies (~38%), which may have led to divergent information reporting. Moreover, the questionnaires used for the father‐specific variables were heterogeneous across the eligible studies; this made it difficult to compare the equivalent results and to quantitatively collate the results through a meta‐analysis. The information about the standardization of these questionnaires was not clearly presented in 11 studies (38%), thus restricting to obtain more reliable results with regards to the relevant paternal features. Similarly, most of the included studies (63%) did not report whether strategies were utilized to address confounding factors, which would have influenced the interpretation and reliability of the study results. Finally, data on participants' sociodemographic characteristics (i.e., race, ethnicity, SES) were provided in only a few studies. This implies an important limitation of this systematic review regarding the difficulty in determining the diversity level of the study samples.

### 4.3 Future Implications

This systematic review may have implications for further research and clinical practice. Although considering the aforementioned limitations, some findings of this review suggested the relationships between certain paternal factors (e.g., food attitudes, muscularity ratings) and offspring's outcomes (e.g., the risk of developing BN later, body dissatisfaction) (Benninghoven et al. 2007; Fernández‐Aranda et al. 2007). These may imply the importance of investigating the role of father‐specific variables as possible risk or maintaining factors for the offspring's BN, using more robust research methods such as longitudinal study designs. Importantly, studies should endeavor to include gender‐, race‐, ethnically‐, socioeconomic‐, and neuro‐diverse populations, and examine and distinguish between different family structures. This research could guide the development and/or improvement of prevention and intervention programs focusing on these aspects in the family context to support both fathers and their offspring with BN.

Furthermore, the finding about more paternal substance abuse reported by the participants with poor outcome compared with those with good outcome at treatment end (Arikian et al. 2008) might indicate the need for psychological support of fathers. Although this single study did not include data about when paternal substance misuse started (i.e., before or after the emergence of the offspring's BN), the finding of this study would provide valuable information about this aspect of their psychological health and well‐being. Thus, further research can provide a more comprehensive understanding about paternal well‐being and its possible relations with the treatment outcomes.

As far as we know, there is no study in the existing literature that directly compared psychosocial characteristics of fathers and mothers of people with BN. To focus on this research gap seems important to gain an understanding about the similarities and/or differences in individual psychological characteristics of fathers and mothers and their possible impacts on parents' caregiving experiences or the BN symptomatology.

The studies we identified focused mainly on the problems and symptoms of fathers of people with BN, but not on the fathers' abilities, talents, and strategies they developed to support their offspring. Therefore, future research should also examine fathers' strengths and how they may play a protective role against the risk of developing BN, and may help with the treatment and the recovery processes.

Overall, the available literature indicates that fathers of people with BN do not discernibly differ from those of the comparison groups in terms of perceptual body‐size distortion and self‐ideal discrepancy (in body‐fat and muscularity), eating‐/weight‐/body‐related attitudes, several personality and ED traits, and general psychological functioning. However, some studies suggest differences in certain psychological aspects (e.g., impulse regulation) and ED‐associated variables (e.g., body dissatisfaction). Several studies found relationships between the fathers' food attitudes, muscularity ratings, personality traits, and substance abuse and the offspring's risk of developing BN later, body dissatisfaction, ED‐associated features, and poor outcome at treatment end. Further longitudinal research should be designed to interrogate whether or how these father‐specific psychosocial and psychopathological variables are related to the offspring's BN symptomatology as risk and/or maintaining factors. Additionally, future research would benefit from identifying paternal strengths, including the ways in which fathers help their offspring with BN, how they can support their treatment and/or recovery process and relapse prevention. This knowledge can direct the development of more holistic prevention and/or treatment programs to facilitate an active role of the fathers in their own well‐being and the offspring's recovery.

## Author Contributions

**M. N. Akkese:** conceptualization, data curation, funding acquisition, investigation, methodology, project administration, writing – original draft, writing – review and editing. **J. L. Keeler:** conceptualization, methodology, supervision, writing – review and editing. **J. Y. Teh:** investigation. **J. Treasure:** conceptualization, supervision, writing – review and editing. **H. Himmerich:** conceptualization, supervision, writing – review and editing.

## Conflicts of Interest

The authors declare no conflicts of interest.

## Supporting information


**Data S1.** Supporting Information.

**Data S2.** Supporting Information.

**Data S3.** Supporting Information.

**Data S4.** PRISMA Checklist.

## Data Availability

Data sharing are not applicable to this article as no new data were created or analysed in this study.
