# Supplementary material for: Psychological Characteristics of Fathers of People With Bulimia Nervosa: A Systematic Review
Source: Int J Eat Disord. 2024 Nov 26;58(2):261–90. doi: 10.1002/eat.24333 (PMC11861882; doi:10.1002/eat.24333)
Supplement: Supplementary file 1 — Data S1. Supporting Information. [file EAT-58-261-s003.docx]

| **Table S1** The Database-specific Search Queries |
| --- |
| 1. APA PsycINFO <1806 to 2024> |
| 1. exp Bulimia/ 8280  2. bulimi*.mp. (mp=title, abstract, heading word, table of contents, key concepts, original title, tests & measures, mesh word) 14702  3. exp Expectant Fathers/ or exp Adolescent Fathers/ or exp Single Fathers/ or exp Fathers/ 12987  4. father*.mp. (mp=title, abstract, heading word, table of contents, key concepts, original title, tests & measures, mesh word) 56432  5. exp Childhood Development/ or exp Family/ or exp Parental Involvement/ or exp Child Care/ or exp Parenting/ or exp Child Abuse/ or exp Caregivers/ or exp Father Child Relations/ or exp Parent Child Relations/ or exp Fathers/ 512934  6. "dad*".mp. (mp=title, abstract, heading word, table of contents, key concepts, original title, tests & measures, mesh word) 6197  7. exp Attachment Behavior/ or exp Family/ or exp Parenting/ or exp Parental Investment/ or exp Father Child Relations/ or exp Risk Factors/ or exp Parent Child Relations/ or exp Fathers/ or exp Parental Role/ or exp Childhood Development/ 578455  8. patern*.mp. (mp=title, abstract, heading word, table of contents, key concepts, original title, tests & measures, mesh word) 18064  9. exp Parent Child Relations/ or exp Child Abuse/ or exp Family Relations/ or exp Adolescent Development/ or exp Family Structure/ or exp Fathers/ or exp Marital Relations/ or exp Relationship Quality/ or exp Stepfamily/ or exp Father Child Relations/ or exp Stepparents/ or exp Biological Family/ or exp Adult Offspring/ or exp Stepchildren/ 302709  10. stepfather*.mp. (mp=title, abstract, heading word, table of contents, key concepts, original title, tests & measures, mesh word) 886  11. exp Family/ or exp Father Child Relations/ or exp Childhood Development/ or exp Parent Child Relations/ or exp Stepparents/ or exp Sexual Abuse/ or exp Remarriage/ or exp Family Structure/ or exp Adolescent Development/ or exp Child Abuse/ or exp Fathers/ or exp Father Absence/ or exp Family Relations/ 547438  12. "step-father*".mp. (mp=title, abstract, heading word, table of contents, key concepts, original title, tests & measures, mesh word) 83   - 1. or 2 14702   14. 3 or 4 or 5 or 6 or 7 or 8 or 9 or 10 or 11 or 12 705068  15. 13 and 14 |
| 1. ISI Web of Science <1991 to 2024> |
| ALL= (bulimia nervosa OR bulimi*) AND ALL= (father* OR dad* OR patern* OR stepfather* OR step-father* OR  "step-father*") |
| 1. PubMed <1985 to 2024> |
| (father(MeSH Terms) OR father child relation(MeSH Terms) OR father child relations(MeSH Terms) OR father child relationship(MeSH Terms) OR father child relationships(MeSH Terms) OR father* OR "dad" OR age, paternal(MeSH Terms) OR ages, paternal(MeSH Terms) OR behavior, paternal(MeSH Terms) OR behaviors, paternal(MeSH Terms) OR deprivation, paternal(MeSH Terms) OR patern* OR stepfather* OR "step-father*" OR single step parent(MeSH Terms)) AND (bulimia(MeSH Terms) OR bulimia nervosa(MeSH Terms) OR bulimias(MeSH Terms) OR nervosa, bulimia(MeSH Terms) OR bulimi*) |
| 1. Google Scholar^†^ < anytime > |
| - (father* OR dad OR stepfather OR patern*) AND (bulimi* OR "bulimia nervosa") AND (thesis) - (father OR dad OR stepfather OR paternal) AND (bulimia OR "bulimia nervosa") AND (thesis) - (father* OR dad OR stepfather OR patern*) AND (bulimi* OR "bulimia nervosa") AND (conference) - (father OR dad OR stepfather OR paternal) AND (bulimia OR "bulimia nervosa") AND (conference) - (father* OR dad OR stepfather OR patern*) AND (bulimi* OR "bulimia nervosa") AND (preprint) - (father OR dad OR stepfather OR paternal) AND (bulimia OR "bulimia nervosa") AND (preprint) |
| 1. ResearchGate^†^ < anytime > |
| (father* OR dad OR stepfather OR patern*) AND (bulimi* OR "bulimia nervosa") all types |
| 1. Open Science Framework^†^ < anytime > |
| - (father*) AND (bulimi*) ALL - (father) AND (bulimia) ALL - (patern*) AND (bulimi*) ALL - (paternal) AND (bulimia) ALL - (dad) AND (bulimi*) ALL - (dad) AND (bulimia) ALL - (stepfather*) AND (bulimi*) ALL - (stepfather) AND (bulimia) ALL |

^†^ The first 20 pages of search results were screened for the grey literature materials (i.e., theses, conference proceedings, preprint manuscripts).
