# Supplementary material for: Psychological Characteristics of Fathers of People With Bulimia Nervosa: A Systematic Review
Source: Int J Eat Disord. 2024 Nov 26;58(2):261–90. doi: 10.1002/eat.24333 (PMC11861882; doi:10.1002/eat.24333)
Supplement: Supplementary file 2 — Data S2. Supporting Information. [file EAT-58-261-s001.docx]

| **Table S2.1** JBI Quality Assessment Results – Cross-sectional Studies | | | | | | | | | |
| --- | --- | --- | --- | --- | --- | --- | --- | --- | --- |
| Reference | Q1: Were the criteria for inclusion in the sample clearly defined? | Q2: Were the study subjects and the setting described in detail? | Q3: Was the exposure measured in a valid and reliable way? | Q4: Were objective, standard criteria used for measurement of the condition? | Q5: Were confounding factors identified? | Q6: Were strategies to deal with confounding factors stated? | Q7: Were the outcomes measured in a valid and reliable way? | Q8: Was appropriate statistical analysis used? | Overall Appraisal |
| Amianto et al. (2015) | Y | Y^a^ | Y | Y | U | U | Y | Y | Included |
| Benninghoven  et al. (2007) | U | Y^a^ | Y | Y | U | U | Y | Y | Included |
| Birckhead (1990) | Y | Y^a^ | U | Y | U | U | Y | Y | Included |
| Bonal (1990) | Y | Y^a^ | U | Y | U | U | U | Y | Included |
| Bonne et al. (2003) | U | Y^a^ | Y | Y^b^ | U | U | U | Y | Included |
| Carney et al. (1990) | Y | Y^a^ | Y | Y | U | U | Y | Y | Included |
| Dyrenforth (1990) | Y | Y^a^ | Y | Y^c^ | U | U | U | Y | Included |
| Elliott-Harper (1984) | Y | Y | U | Y^c^ | Y | Y | U | Y | Included |
| Espina (2003) | Y | Y^a^ | Y | Y^c^ | Y | Y | Y | Y | Included |
| Espina et al. (2003) | Y | Y^a^ | Y | Y^b^ | Y | Y | U | Y | Included |
| Fassino et al. (2003) | U | Y^a^ | Y | Y | Y | N/A | Y | Y | Included |
| Fassino et al. (2009) | Y | Y^a^ | Y | Y | Y | Y | Y | Y | Included |
| Gómez-Castillo  et al. (2017) | U | Y^a^ | Y | Y | Y | U | Y | Y | Included |
| Gómez-Castillo  et al. (2018) | U | Y^a^ | Y | Y^b^ | Y | U | U | Y | Included |
| Humphrey (1988) | U | Y^a^ | Y | Y^b^ | U | U | U | Y | Included |
| Kanakis & Thelen (1995) | U | Y^a^ | Y | Y | Y | Y | Y | Y | Included |
| Mangweth et al. (1995) | Y | Y | U | Y | Y | N/A | Y | Y | Included |
| Mark (1993) | Y | Y^a^ | U | Y | U | U | Y | Y | Included |
| Moreno & Thelen (1993) | Y | Y^a^ | U | Y | Y | N/A | Y | Y | Included |
| Pentz (1992) | Y | Y^a^ | U | Y | U | U | U | Y | Included |
| Pisetsky et al. (2017) | U | Y^a^ | U | Y | Y | Y | U | Y | Included |
| Pourdehghan  et al. (2024) | Y | Y^a^ | U | Y | Y | Y | Y | Y | Included |
| Ratti (1994) | Y | Y^a^ | Y | Y | U | U | Y | Y | Included |
| Reto (1997) | Y | Y^a^ | U | Y | U | U | Y | Y | Included |
| Schmidt et al. (1993) | Y | Y^a^ | Y | Y | U | U | Y | Y | Included |
| Táfa et al. (2017) | Y | Y^a^ | Y | Y | U | U | Y | Y | Included |
| Woodside  et al. (1998) | Y | Y^a^ | Y | Y | U | U | Y | Y | Included |

^a^The detailed information about the study setting was presented; however, several sociodemographic characteristics of the participants were not clearly reported.

^b^The measurement criteria of the condition used for the control group were unclear, contrary to those used for the BN sample.

^c^The BN group was diagnosed through the DSM criteria, whereas the control group was screened through self-report questionnaires.

*Abbreviations:* N/A: not applicable; Y: Yes; U: Unclear

| **Table S2.2** JBI Quality Assessment Results – Case-control Studies | | | | | | | | | | | |
| --- | --- | --- | --- | --- | --- | --- | --- | --- | --- | --- | --- |
| Reference | Q1: Were the groups comparable other than presence of disease in cases or absence of disease in controls? | Q2: Were cases and controls matched appropriately? | Q3: Were the same criteria used for identification of cases and controls? | Q4: Was exposure measured in a standard, valid and reliable way? | Q5: Was exposure measured in the same way for cases and controls? | Q6: Were confounding factors identified? | Q7: Were strategies to deal with confounding factors stated? | Q8: Were outcomes assessed in a standard, valid and reliable way for cases and controls? | Q9: Was the exposure period of interest long enough to be meaningful? | Q10: Was appropriate statistical analysis used? | Overall Appraisal |
| Fernández-Aranda et al. (2007) | Y | N | Y^a^ | Y | Y | Y | Y | Y | Y | Y | Included |

^a^The BN group was diagnosed through the SCID-I for DSM-IV, whereas the control group was screened through the DSM-IV criteria and the GHQ-28.

*Abbreviations:* N: No; Y: Yes

| **Table S2.3** JBI Quality Assessment Results – Longitudinal Studies | | | | | | | | | | | | |
| --- | --- | --- | --- | --- | --- | --- | --- | --- | --- | --- | --- | --- |
| Reference | Q1. Were the two groups similar and recruited from the same population? | Q2. Were the exposures measured similarly to assign people to both exposed and unexposed groups? | Q3. Was the exposure measured in a valid and reliable way? | Q4. Were confounding factors identified? | Q5. Were strategies to deal with confounding factors stated? | Q6. Were the groups/participants free of the outcome at the start of the study (or at the moment of exposure)? | Q7. Were the outcomes measured in a valid and reliable way? | Q8. Was the follow up time reported and sufficient to be long enough for outcomes to occur? | Q9. Was follow up complete, and if not, were the reasons to loss to follow up described and explored? | Q10. Were strategies to address incomplete follow up utilized? | Q11. Was appropriate statistical analysis used? | Overall Appraisal |
| Arikian et al. (2008) ^a^ | Y | Y | U | Y | Y | N/A | Y | N/A | U | U | Y | Included |

^a^The quality assessment of this study was conducted based on the JBI critical appraisal tool of cohort studies due to the lack of the relevant tool for a longitudinal study.

*Abbreviations:* N/A: not applicable; Y: Yes; U: Unclear
