# Supplementary material for: Psychological Characteristics of Fathers of People With Bulimia Nervosa: A Systematic Review
Source: Int J Eat Disord. 2024 Nov 26;58(2):261–90. doi: 10.1002/eat.24333 (PMC11861882; doi:10.1002/eat.24333)
Supplement: Supplementary file 3 — Data S3. Supporting Information. [file EAT-58-261-s004.docx]

**Excluded non-English Studies (N= 225)**

1. Alantar, Z., & Maner, F. (2008). Eating disorders in the context of attachment theory. *Anadolu Psikiyatri Dergisi, 9*(2), 97-104. Retrieved from <http://ovidsp.ovid.com/ovidweb.cgi?T=JS&PAGE=reference&D=psyc7&NEWS=N&AN=2008-09120-006>.

**Abstract**

Objective: In this review article eating disorders were examined in the context of attachment theory. Methods: Attachment theory focuses on the mother-infant relationship and its effects on child’s behavioral development. Results: Unstability in child’s earliest relationship with caregivers may lead to eating disorders. From this perspective, since food consumption usually replaces parental affection, the patient believes that she/he can control and arrange it both qualitatively and quantitatively. Eating disordered individuals are considered to fail frequently in establishing emotional communication with the persons they ‘attached’ and also to have built insecure attachments during childhood. Although insecure attachment is frequent among eating disordered patients when compared to non-eating disordered individuals, relationship between risk factors which may lead to eating disorders and nature of attachment in pre-adolescence period have not been thoroughly studied. Struggling extensively with fears of death plays a major role in the onset and development of eating disorders, especially anorexia nervosa. Fear from weight gaining and hidden symptoms of anorexia and bulimia nervosa may serve to protect the patient from the fear of death. Conclusion: Both eating disordered and insecurely attached individuals have low self-esteem, feeling of insufficiency and incapability.

1. Alvarez Tapia, D., Franco Pardeds, K., Mancilla Diaz, J. M., Alvarez Rayon, G., & Lopez Aguilar, X. (2000). Predictive factors for eating disorder symptoms. *Psicologia Contemporanea, 7*(1), 26-35. Retrieved from <http://ovidsp.ovid.com/ovidweb.cgi?T=JS&PAGE=reference&D=psyc3&NEWS=N&AN=2000-15044-003>.

**Abstract**

Studied predictive factors for eating disorders in 912 male and female university students (mean age 18.3 yrs) in Mexico. Data on sociodemographic variables and eating behavior were obtained by questionnaire, using the Eating Disorders Inventory and the Eating Attitudes Test (D. M. Garner and P. E. Garfinkel, 1979). Factor structure and internal consistency were determined. Multiple regression analysis was used. Eight factors associated with eating disorders were identified: motivation to be thin, lack of interpersonal confidence, body dissatisfaction, fear of growing up, bulimia, ineffectiveness, difficulty in recognizing emotions, and perfectionism. The results indicate that motivation to be thin, body dissatisfaction, bulimia, and ineffectiveness are risk factors for this population. The results are discussed in relation to results from other studies of risk factors for eating disorders.

1. Amianto, F., Bertorello, A., Marech, L., Pagnanelli, R., Daga, G. A., & Fassino, S. (2012). Family functioning and eating disorders. *Minerva Psichiatrica, 53*(4), 333-342. Retrieved from <http://ovidsp.ovid.com/ovidweb.cgi?T=JS&PAGE=reference&D=psyc11&NEWS=N&AN=2013-10227-006>.

**Abstract**

Objective: Our study evaluates the perception of family functioning in the family members of patients with eating disorders and analyzes the specific aspects that differentiate diagnostic subgroups: anorexia nervosa (AN) and bulimia nervosa (BN).  Methods: 171 subjects (103 affected by AN and 68 by BN) were assessed at the admission with regard to depressive symptoms, eating psychopathology and family functioning through the following psychometric tests: the Beck Depression Inventory (BDI), the Eating Disorder Inventory (EDI-2) and the Family Assessment Device (FAD). Even the parents of the patients were assessed the test FAD.  Results: The mothers of patients with eating disorders report the more positive view of family functioning while patients are the family member with the most critical perception. In patients with BN are found more pathological scores compared to patients suffering from AN, with significant differences in family roles and affective involvement that persist using as confounding depressive symptoms persist assessed with the BDI, test. The perception of family functioning assessed by the daughters correlates well with their eating psychopathology, while a few correlations are found with FAD scores of parents.  Conclusions: The study highlights the influence of family dynamics on the eating psychopathology of patients and emphasizes the presence of a low awareness on altered family dynamics, in mothers of patients confirmed as an immature parental figure.

1. Anzieu, A. (1995). Troubling femininity. Adolescence in girls. *Psyche: Zeitschrift fur Psychoanalyse und ihre Anwendungen, 49* (9-10), 886-902. Retrieved from <http://ovidsp.ovid.com/ovidweb.cgi?T=JS&PAGE=reference&D=psyc3&NEWS=N&AN=1996-85940-001>.

**Abstract**

Stresses the importance of adolescence for the establishment of female identity. The author outlines a somatopsychic relation model, emerging between mother and daughter at birth, and based on unconscious projections of the mother. The model is structured around the contrast between the image of the body as envelope and as (empty) cavity. During the course of adolescence, in which femininity, sexuality, and motherhood should be represented and combined in the female body, the female adolescent must relinquish the idea of her body as a cavity and identify with the idea of physical containment. The clinical case of a bulimic adolescent illustrates the arduous path toward the appropriation of femininity, which, if unsuccessful, can lead to the edge of death.

1. Arevalo, R. V., Diaz, J. M. M., Gonzalez, C. M., Aguilar, X. L., Alvarez Rayon, G. L., Martinez, A. O. R., & Paredes, K. F. (2005). Eating disorders and risk factors in an incidental sample of young Mexicans. *Revista Mexicana de Psicologia, 22*(1), 53-63. Retrieved from <http://ovidsp.ovid.com/ovidweb.cgi?T=JS&PAGE=reference&D=psyc5&NEWS=N&AN=2005-09045-005>.

**Abstract**

The objective of the present investigation was to detect the presence of eating disorder and its risk factors in sample incidental of young Mexican. 525 young (n=288 women and n=237 men) with an age average of 15 years (SD=3) participated, they completed five questionnaires regarding to eating attitudes (EAT-40), body dissatisfaction (BSQ), attitudes toward aesthetic body shape model (CIMEC), behavioral and cognitive aspects (EDI) and bulimic behavior (BULIT). Moreover, all subjects were administered a diagnostic interview. The results indicated that 7 women reported eating disorders (ED, 2 Anorexia Nervosa and 5 Bulimia Nervosa) and 22 presented Eating Disorders Not Otherwise Specified (EDNOS, 19 women and 3 men). The best predictor factor for ED in woman was body dissatisfaction and for EDNOS in the men ineffectiveness.

1. Arevalo, R. V., & Escursell, R. M. R. (1997). The role of family in eating disorders. *Psicologia Conductual: Revista Internacional de Psicologia Clinica de la Salud, 5*(3), 391-407. Retrieved from <http://ovidsp.ovid.com/ovidweb.cgi?T=JS&PAGE=reference&D=psyc3&NEWS=N&AN=1997-39079-004>.

**Abstract**

Examined 3 dimensions of family life relevant to eating disorders: parental attitudes toward weight, family dynamics, and eating patterns. 16 families participated (mother, father, and daughter [mean age 21.6 yrs] with an eating disorder) were given 3 tests pertaining to family environment, attitudes toward body shape, and eating patterns. The results show that there is a similarity between mothers and daughters regarding family perceptions and eating patterns. Daughters were more vulnerable to social stereotypes than their parents and they differed significantly with their fathers in their eating patterns.

1. Arevalo, R. V., Escursell, R. M. R., Viladrich i Segues, M. C., Alvarez Rayon, G. L., & Diaz, J. M. M. (2001). Three aspects of family life associated to eating disorders. *Revista Mexicana de Psicologia, 18*(3), 325-335. Retrieved from <http://ovidsp.ovid.com/ovidweb.cgi?T=JS&PAGE=reference&D=psyc3&NEWS=N&AN=2003-99047-005>.

**Abstract**

It is generally accepted that the causes of eating disorders constitute a complex set of phenomena involving the interplay of biological, psychological and social factors. Within this etiological framework, family relationships have received considerable attention as either risk or maintenance factors. In an attempt to identify family factors that seem specific to eating disorders, three dimensions of family life were compared: Family dynamics, attitudes toward the aesthetic body shape model and eating habits. Sixty-eight Spanish families participated (34 had one daughter with an eating disorder and 34 acted as control group). Mothers, fathers and daughters of each family answered three scales. Data were analyzed in relation to the presence of the disorder using a stepwise linear discriminate function analysis. A high presence of worry regarding the aesthetic body shape model and less importance given to the development dimension within the family dynamic discriminated eating disordered daughters from control group daughters.

1. Arevalo, R. V., Ruiz Martinez, A. O., Rayon, G. A., Diaz, J. M. M., & Antonio, T. S. (2010). Family functioning perception of women with eating disorders. *Behavioral Psychology, 18*(1), 105-117. Retrieved from <http://ovidsp.ovid.com/ovidweb.cgi?T=JS&PAGE=reference&D=psyc9&NEWS=N&AN=2010-07995-006>.

**Abstract**

The aim of the present study was to compare the perception of family functioning in Mexican women with eating disorders (ED): anorexia nervosa (AN), bulimia nervosa (BN) and eating disorders not otherwise specified (EDNOS) with a control group. One hundred young women (M= 18.24 years, TD= 3.9) participated in this study; 70 of which met DSM-IV criteria for an ED (16 AN, 24 BN, 30 EDNOS) and 30 who presented no ED (control group). Participants completed a structured interview (IDED) for assessment of eating disorder psychopathology as well as three questionnaires (EAT-40, BULIT and FES). The ED groups perceived that in their families, acting was more important than cohesion; moreover, they presented less expressivity. In conclusion, the ED groups differ from the control group in terms of hierarchy and intensity in each familiar aspect evaluated. The EDNOS and AN groups were similar and the BN group is the one that presents the most problems.

1. Attili, G., Di Pentima, L., & Magnani, M. (2004). Eating disorders, mental models of attachment and family relationships. *Psicologia Clinica dello Sviluppo, 8*(1), 69-96. Retrieved from <http://ovidsp.ovid.com/ovidweb.cgi?T=JS&PAGE=reference&D=psyc5&NEWS=N&AN=2004-15045-004>.

**Abstract**

Studied the relation of attachment processes, family relationships, and eating disorders among 36 male and female adolescents (aged 13-18 yrs) diagnosed with eating disorders (restricted anorexia, binge/purging anorexia, and bulimia nervosa) and 28 male and female adolescents (aged 14-19 yrs) without diagnoses of eating disorders (controls) in Italy. Data on sociodemographic variables, psychological symptoms, and family dynamics were obtained using the Symptom Checklist-90 (Derogatis et al, 1973), Eating Attitudes Test-40 (D. M. Garner and P. E. Garfinkel, 1979), Parental Bonding Instrument (G. Parker et al, 1979), and Separation Anxiety Test (M. Klagsbrun and J. Bowlby, 1976; Italian modification, G. Attili, 2001). The results suggest that restricted anorexia is related to avoidant/dismissing attachments and a dismissive idealization of parents, purging anorexia is associated with confused attachments and maternal parenting as optimal but paternal parenting as weak or absent, and bulimia is linked to disorganized attachments and maternal parenting as caring and protective but paternal parenting as less caring and more protective. Implications for treatment are discussed.

1. Bahrke, U., Arends, M., Bandemer-Greulich, U., Dreyer, B., Ropke, H., & Fikentscher, E. (2003). The Incidence of Pathological Eating Behaviour among Schoolchildren in a Large Urban Area. *PPmP: Psychotherapie Psychosomatik Medizinische Psychologie, 53*(1), 29-34. <https://doi.org/10.1055/s-2003-36481>

**Abstract**

Clinical experience and international epidemiological studies indicate an increase in pathological eating behaviour as well as a shift towards increasingly younger children. In order to study its frequency in Germany a large random sample, 2844 pupils of the 10th class level were contacted as part of a school medical examination to take part in a questionnaire study using the Eating Disorder Inventory. Age, gender, BMI and the type of school were also recorded. From the statistical description of healthy matching samples, gender-specific cut-off values were established and this methodical approach was discussed critically. Of the predominantly 15/16-year-old schoolchildren, 83% took part in the study. Regardless of gender and type of school, more than 7% had an anorexic body weight and 10% of female and 2% of male pupils had bulimic eating behaviour.

1. Bailly-Lambin, I., & Bailly, D. (1999). Separation anxiety disorder and eating disorders. *L'Encephale: Revue de psychiatrie clinique biologique et therapeutique, 25*(3), 226-231. Retrieved from <http://ovidsp.ovid.com/ovidweb.cgi?T=JS&PAGE=reference&D=psyc3&NEWS=N&AN=1999-03505-004>.

**Abstract**

Conducted an epidemiological retrospective study of 81 inpatients in France with anorexia nervosa (AN) or bulimia nervosa (BN) according to Diagnostic and Statistical Manual of Mental Disorders-III-Revised (DSM-III-R) criteria to investigate the possible association between eating disorders and childhood separation anxiety disorder (SAD). The current and lifetime psychiatric histories of 4 male and 41 female adolescents and adults (aged 13-42 yrs) with AN and 1 male and 35 female adolescents and adults (aged 14-47 yrs) with BN were assessed with the Structured Clinical Interview for DSM-III-R Personality Disorders and the Schedule for Affective Disorders and Schizophrenia. Psychopathological profiles were assessed with the SCL-90. The results show that approximately 20% of Ss with AN and BN had a history of childhood SAD. This subgroup differed significantly from Ss without this history in terms of associated anxiety and depressive disorders.

1. Bak, D. (2008). Eating disorders in men. Psychiatria Polska, *42*(2), 167-178. Retrieved from <http://ovidsp.ovid.com/ovidweb.cgi?T=JS&PAGE=reference&D=psyc7&NEWS=N&AN=2011-25228-002>.

**Abstract**

Despite of being perceived as 'woman's diseases', eating disorders were described among boys and adult men. This article presents epidemiological data on anorexia nervosa, bulimia nervosa and binge eating disorder in men. The clinical presentation of eating disorders in men was described and compared with similar data from the female population. Moreover, a significance of selected risk factors, specifically those referring to men, was discussed. These are: the disturbance of body perception, personality traits and potential association of eating disorders with sexual orientation. Efficacy of different psychotherapy approaches aimed at eating disorders was summarized. Rules governing psychotherapy of men suffering from eating disorders were described. Specific features of eating disorders' aetiology were taken into account together with characteristic difficulties influencing treatment.

1. Baltruschat, N., Geissner, E., & Klein, M. (2005). Parental alcohol misuse. Risk factors for eating disorders in their daughters. *Zeitschrift fur Gesundheitspsychologie, 13*(2), 58-68. <https://doi.org/10.1026/0943-8149.13.2.58>

**Abstract**

Background: In the last two decades psychological effects on children of alcoholics (COA) have gained importance.  Objective: The question of the present study is, could growing up in a household with an alcoholic or alcohol-abusing parent increase the risk for their daughters developing an eating disorder? Recent studies analyzed: (a) if women with an eating disorder have higher rates of parents with drinking problems (b) if daughters of parents with alcohol problems are at more risk of developing an eating disorder. Results up until now have proved inconclusive.  Method: Following two different ways of examination: (a) assessement of 100 women with an eating disorder (13-26 years) by clinical interviews and questionnaires ([1] family history, [2] Children of Alcoholics Screening Test (CAST); (b) assessement of 47 alcohol-abusing parents to see if they have noticed symptoms of eating disorders in their daughters ([1] clinical interviews, [2] Eating Attitudes Test 26 and {3] Subscales of the Eating Disorder Inventory - EDI from the parents about their daughters) and additional inquiry of their daughters (N = 54) [EAT-26, EDI, CAST]. Results: The results could verify our hypothesis about a connection between eating disorder in daughter and parental alcohol-abuse. In sample (a) daughters we found a rate of parental alcohol-abuse of 47% (baseline of the population 1.1-4%). In sample (b) (parents with an alcohol problem) we found a rate of daughters with symptoms of an eating disorder of aproximately 40% (Rates of eating disorders of young women, e.g., 3% bulimia nervosa, 10% "less severe" symptoms of an eating disorder). Conclusions: Parental alcohol-abuse is an important risk factor in their daughters developing an eating disorder. Results are discussed against the background of familiar socialisation and the general transmission of psychological diseases.

1. Baltruschat, N., Klein, M., & Geissner, E. (2009). Eating Disorders of Daughters as a Consequence of Paternal Alcoholism. *Suchttherapie, 10*(2), 81-90.

**Abstract**

Background: In the present retrospective study, we analyzed the familial risk factors – especially parental alcoholism – for daughters for an eating disorder with a structured clinical interview (Structural clinical interview; SKID). Emotional stress for children, growing up or living with alcoholic parents is analyzed by questionnaire (Children of alcoholics screening test CAST [1],). Objective: The question of the present study is, if growing up in a household with an alcoholic or alcohol-abusing parent could increase the risk for their daughters developing an eating disorder? In a recent study [2] parental alcohol abuse could be found as an important risk factor for their daughters developing an eating disorder. Until now it is not clear, if parental alcohol abuse or other mental disorders are a transmission factor for mental disorders for their daughters. (a) if women with an eating disorder have higher rates of parents with drinking problems (b) if daughters of parents with alcohol problems are at more risk of developing an eating disorder. Results up until now have proved inconclusive. Method: Following two different ways of examination: (a) Assessment of 39 women with an eating disorder (13-26 years) by structural clinical interviews and questionnaires ([1] SKID [2], Children of Alcoholics Screening Test (CAST, scale I “intrapsychical stress” and scale 2 “interactional stress”) [3], Eating Attitudes Test (EAT) [4], subscales of the Eating Disorder Inventory (EDI)) and their parents with structural clinical interviews (SKID); (b) Assessment of 35 healthy women (16-27 years) by additional inquiry of themselves and their parents. Eating disordered patients where at time of questioning attending therapy in hospitals. Results: The results could verify our hypothesis about a connection between an eating disorder of the daughters and parental alcohol-abuse. In sample (a) (eating disordered patients) we found a higher rate of parental alcohol-abuse compared with the control group (7 parents [15 %] of eating disordered women and none of the control group; baseline of the population 1.1-4%). We also found higher rates of depression and an eating disorder of the mothers of patients in contrast to the parents of the control group. Conclusions: Parental alcohol-abuse is an important risk factor for their daughters developing an eating disorder. Results also show that young women are emotionally stressed by depression and eating disorders of the mothers.

1. Barone, L., & Guiducci, V. (2008). Mental representations of attachment in anorexia: A study of the Adult Attachment Interview. *Infanzia e Adolescenza, 7*(2), 87-97. Retrieved from <http://ovidsp.ovid.com/ovidweb.cgi?T=JS&PAGE=reference&D=psyc7&NEWS=N&AN=2009-11194-003>.

**Abstract**

Objective: The main purpose of the study was to assess mental representations of attachment in a sample of adults with Eating Disorders (ED) and to identify differences between anorexia nervosa and other ED subgroups.  Method: sixty-five subjects (30 non-clinical and 35 clinical) were assessed using the Adult Attachment Interview (AA1). 15 subjects had a diagnosis of anorexia, 10 of bulimia and 10 of binge eating disorder.  Results: Within the clinical group Free/autonomous subjects represented only 8 %, Dismissing classifications reached about 46%, Entangled/Preoccupied accounted for around 23% and Disorganized patterns obtained about 23%. The two samples differed in their attachment patterns distribution and were significantly different in some coding system subscales. Entangled/Preoccupied classification was overrepresented in Anorexia Nervosa subgroup (53%), whereas no subjects were classified as Unresolved or Cannot Classified. Further evidence was obtained analysing differences between Anorexia and the other ED subtypes: father rejecting and passivity were the risk factors that discriminate the two groups. Concerning the differential role of the two parental figures in the development of attachment representations our data suggest the importance of father neglecting in anorexia nervosa group, whereas, in addition to this dimension, the relevance of mother loving and role-reversing in the other ED subgroups.  Conclusions: Findings related to anorexic subjects underline the importance of Preoccupied pattern, the absence of Disorganized classifications and the specific differential role played by the two parental figures.

1. Beato-Fernandez, L., Rodriguez-Cano, T., Belmonte-Llario., A., & Pelayo-Delgado, E. (2005). Factores de riesgo y de protection para el uso de toxicos en tudioents. Un tudio longitudinal. *Actas Espanolas de Psiquiatria, 33*(6), 352-358. Retrieved from <http://ovidsp.ovid.com/ovidweb.cgi?T=JS&PAGE=reference&D=psyc5&NEWS=N&AN=2005-16310-002>.

**Abstract**

Introduction: The aim of the present study was to identify possible risk and protective factors for the development of problems with the use of drugs in adolescents.  Methods: A two-year follow-up was carried out (from the 2nd to the 4th year of the Compulsory Secondary Education). Sociodemographic variables, general and eating psychopathology, family functioning and patterns of drugs use were assessed in 1,076 students.  Results: After controlling for the effect of having problems with the use of drugs at the beginning, cigarette smoking at the age of 13 years predicted the consumption of alcohol 2 years later, and vice versa. Independently of the effect of this association, general psychopathology, body image dissatisfaction and self-harm at the beginning were risk factors for alcohol consumption 2 years later. Moreover, besides female gender and high academic achievement, normal family functioning was a protective factor against the fact of suffering problems with drugs later on. 
Conclusions: These present findings might have relevance in the development of preventive strategies for the use of drugs in adolescent population.

1. Bellini, M., & Bove, P. (2003). Impact of child sexual abuse in anorectic patients. *Minerva Psichiatrica, 44*(4), 207-215. Retrieved from <http://ovidsp.ovid.com/ovidweb.cgi?T=JS&PAGE=reference&D=psyc4&NEWS=N&AN=2004-12397-002>.

**Abstract**

This is a critical review of the relationship between childhood sexual abuse and anorexia nervosa, which is differentiated by the overall eating-disorders category. Referring to abused persons, many authors believe that a non-specific risk is present, eliciting anorexia and being similar to the risk for other psychiatric disorders. Among eating-disordered patients, the mentioned abuse is known mainly to involve the bulimic ones; as for anorectic patients, it is found as well, but more frequently in the bingeing/purging subtype than in the restrictive subtype. It depends on the fact that sexual abuse is much more related to bingeing and purging. Moreover, sexual vs physical or psychological childhood abuses are discussed. Among many variables, the impact of family support is considered on patients with a history of sexual abuse. When the abused child is protected by his/her family, he/she is more able to overcome the trauma with fewer psychopathologic consequences. On the contrary, when the family ignores requests for help or criticizes the victim, the psychopathologic effects increase. Fewer authors point out that child sexual abuse is a specific risk factor for subsequent development of eating disorders, particularly when co-occurring with a younger age-cohort under possible influence of environmental variables.

1. Bellino, S., Bozzatello, P., Blandamura, A., & Bogetto, F. (2010). Psychotherapy of borderline personality disorder: Current trends and research issues. *Giornale Italiano di Psicopatologia / Italian Journal of Psychopathology, 16*(1), 11-26. Retrieved from <http://ovidsp.ovid.com/ovidweb.cgi?T=JS&PAGE=reference&D=psyc9&NEWS=N&AN=2010-12346-002>.

**Abstract**

Borderline personality disorder (BPD) is an Axis II mental disorder characterized by a pervasive pattern of instability in interpersonal relationships, affects, and identity, as well as severe impulsive behaviors. According to literature data and treatment guidelines of the American Psychiatric Association, borderline personality disorder can be usefully treated with both pharmacotherapeutic and psychotherapeutic interventions. Clinical experience suggests that patients with BPD need extended psychotherapy (at least 7 year) to attain and maintain lasting improvement in psychiatric symptoms, interpersonal problems, and overall functioning. Main models of psychotherapy that have been tested for BPD treatment in trials of single or combined therapy are: psychodynamic psychotherapy, dialectical behavior therapy (DBT), schema focused therapy (SFT) and system training for emotional predictability and problem solving (STEPPS) interpersonal psychotherapy (IPT). The present review aims to report and comment results of clinical trials assessing the efficacy of the above listed models of psychotherapy in the treatment of borderline personality disorder. Psychodynamic psychotherapy is a therapy that is focused on the transference relationship. The therapist provides interpretations of transference choosing the most suitable times in the therapeutic process. Authors proposed different approaches to psychodynamic therapy of borderline patients, preferring the use of supportive or explorative techniques. Psychodynamic psychotherapy is recommended for treatment of patients with BPD on the basis of the positive results of several randomized controlled trials. However, the use of this model of psychotherapy in clinical practice is limited, probably due to the high number of resources required to both therapists and the patients. Dialectical behavior therapy is a model proposed by Linehan and derived from cognitive-behavioral therapy. It was specifically designed to treat women who meet diagnostic criteria for BPD and engage in self-injurious behaviors. DBT can be currently considered the psychotherapy with the largest number of controlled studies in BPD samples. It has been found to be efficacious in the treatment of high-risk behaviors and suicide attempts, but it is not so useful in reducing depressive symptoms and increasing patients' degree of overall satisfaction. DBT has been also studied to treat BPD patients with substance abuse or eating disorders comorbidity. Interpersonal therapy was initially proposed to treat major depression, but has been more recently considered a good option to treat other psychiatric disorders, such as bipolar disorder, bulimia nervosa, and anxiety disorders. Markowitz (2006) proposed an adaptation of IPT to the peculiar clinical and relational features of patients with borderline personality disorder (IPTBPD). Interpersonal therapy appears a suitable approach to these patients, who show prominent relational problems and high comorbidity rates with major depression. Growing clinical evidence supports combination of psychotherapy and pharmacotherapy in treatment of BPD, but only few controlled trials comparing efficacy of combined and single therapy have been completed. Borderline personality disorder is characterized by various psychopathological dimensions, that could be more responsive to specific models of psychotherapy, to different drug interventions or to combined or sequential therapy. Further investigations are required to collect data on this core issue of BPD treatment, identifying response predictors and designing more individualized treatment projects.

1. Berdah, C. (2010). Obesity and psychiatric disorders. *Annales Medico-Psychologiques, 168*(3), 184-190.

**Abstract**

Obesity is defined as being overweight from an excess of fat with harmful effects on health. It is a chronic disorder of complex etiology, a serious risk factor, which compromises psychosocial functioning and the patient's quality of life. Physical corporal satisfaction and dissatisfaction with regard to one's body are two distinct aspects of the concept of bodily image. The major characteristic of dissatisfaction of social origin is the sensation of not being attractive; therefore, it is not an isolated cognition but truly one that reasons in reference to others, bringing avoidance behaviors as well as explaining a high probability of social phobia and agoraphobia in obese subjects. The dissatisfaction of an obese subject regarding his/her body seems to depend on several factors. Indeed, this disorder seems to be more frequent in female subjects suffering from bulimia and searching for help to lose weight, but the studies remain contradictory.

1. Bessoles, P. (2005). Vulnerability and sexual aggressions about child under 15 years old. *Pratiques Psychologiques, 11*(4), 371-385. <https://doi.org/10.1016/j.prps.2005.09.006>

**Abstract**

The present preliminary study concern immediately post traumatic disorder about 60 children under age 15 years (sex and age are not considered). This research purpose the hypothesis of specific pathology. The analysis of results concerns symptomatic frequency during the first clinical interview. The research objective is not, about this article, a factorial or correlation study but pathological investigation. The objective is a contribution to a creation of clinical investigation scale. A prime time of investigation concern a circumscribed area about syndrome classes. A second time concern the item of each syndrome class. The argumentation compares the frequency of clinical signs with others research like "the Finkelhor and Browne Model". Our research shows a pathological post traumatic entity about child under 15 years. The specificity of pathological frequency concern learning troubles binding psychic inhibitions, body image troubles particular bulimia behavior, paranoid anguish and invalidate phobia.

1. Binswanger, R. (1996). Child masturbation-a genetic viewpoint, with special reference to anorexia and bulimia nervosa. *Psyche: Zeitschrift fur Psychoanalyse und ihre Anwendungen, 50*(7), 644-670. Retrieved from <http://ovidsp.ovid.com/ovidweb.cgi?T=JS&PAGE=reference&D=psyc3&NEWS=N&AN=1996-00781-004>.

**Abstract**

Examines the functions of child masturbation in the development of narcissism and distinguishes a demarcation function, a compensatory function and a function serving to establish autonomy. The author argues that parental reactions to child masturbation may affect the interactive relationship between the child and the parent representing the primary relational object, thus thwarting or undermining these functions. The result is the appearance of certain symptoms. Binswanger differentiates between "horrified,", "liberal," and "eroticized" parental reactions, relating the 1st to compulsion neurosis, the 2nd to obesity, and the 3rd to anorexia-bulimia. The author illustrates his hypotheses with several cases from his own practice.

1. Bojorquez, R. M. C., Escalante, M. L. A., de la Cruz Cortes Sobrino, M., Arevalo, R. V., & Diaz, J. M. M. (2008). Dietary restriction and risky behaviors for eating disorders in Nutrition students. *Psicologia y Salud, 18*(2), 189-198. Retrieved from <http://ovidsp.ovid.com/ovidweb.cgi?T=JS&PAGE=reference&D=psyc7&NEWS=N&AN=2012-09931-005>.

**Abstract**

The objective of the study was to identify the relation between dietary restriction and other risky behaviors for eating disorders in nutrition students of a public university. Three instruments validated in the Mexican population: the Eating Attitudes Test, the Bulimia Test, and the Body Shape Questionnaire were applied to 111 students, 88 women and 23 men, with an average of 20 years of age. The results showed that men exhibited greater symptoms of eating disorders, corporal dissatisfaction, and risky behaviors than women.

1. Bonassi, E. (2003). Anorexia--bulimia nervosa and the paternal function. *Richard e Piggle: Studi Psicoanalitici del Bambino e dell'Adolescente, 11*(1), 31-49. Retrieved from <http://ovidsp.ovid.com/ovidweb.cgi?T=JS&PAGE=reference&D=psyc4&NEWS=N&AN=2003-07410-002>.

**Abstract**

Discusses anorexia nervosa and bulimia as expressions of a depersonalization phenomenon connected with the father figure and paternal functioning. Family status, family dynamics, parent personality, expressed emotion, and parent-child boundary problems are examined in relation to eating disorders.

1. Bosi, M. L. M., Luiz, R. R., da Costa Morgado, C. M., dos Santos Costa, M. L., & de Carvalho, R. J. (2006). Self-perception of body image among nutrition students in Rio de Janeiro. J*ornal Brasileiro de Psiquiatria, 55*(1), 34-40. <https://doi.org/10.1590/S0047-20852006000100005>

**Abstract**

Objective: To characterize risky eating habits and factors related to eating disorders among nutrition students in the city of Rio de Janeiro Methods: Sectional study with a segment of the population pointed out in literature as being at risk of developing eating disorders. The Bulimic Investigatory Test Edinburgh (BITE) questionnaire, the Eating Attitudes Test (EAT-26) questionnaire and a variable which considers the two related tools (Nunes et al, 2001) were used.

1. Bottin, J., Salbach-Andrae, H., Schneider, N., Pfeiffer, E., Lenz, K., & Lehmkuhl, U. (2010). Personality disorders in adolescent patients with anorexia and bulimia nervosa. *Zeitschrift fur Kinder- und Jugendpsychiatrie und Psychotherapie, 38*(5), 341-350. <https://doi.org/10.1024/1422-4917/a000058>

**Abstract**

Objective: The present study aimed to ascertain the occurrence of personality disorders (PD) in adolescent patients with anorexia (AN) and bulimia nervosa (BN) by means of the Structured Clinical Interview for DSM-IV Personality Disorders (SCID-II).

1. Briere, M., Poussevin, C., Vayleux, E., Lefranc, J., Cayrol, B., Garre, J. B., & Gohier, B. (2012). Teenagers and mobile phones, which place during hospitalization? *Annales Medico-Psychologiques, 170*(8), 587-590. <https://doi.org/10.1016/j.amp.2012.08.006>

**Abstract**

Mobile phones occupy a dominating place in society, particularly for teenagers. A spectacular increase of mobile phone used among teenagers occurred, from 8% in 1998 to 94% in 2007. At school, conditions of use are not unanimous and vary from discretion to ban. At the hospital, it belongs to physicians and nurses to define it status. Mobile phone comes to echo the adolescence problems, as demonstrated in the following clinical cases, with a compulsive use and sometime a bulimia of contact. For some teenagers, this bulimia of contact can be addressed to the other teenagers, searching a support, which is not provided by their own family. For others, mobile phone is used to strengthen the link following the example of the fears of separation. The hardship of the telephone can then engender a profound suffering by increasing the feeling of solitude. Others else, subject to a social isolation, cut investment the object, are excluded from groups by cutting the links with the other teenagers to see their own family. Some centers forbid the use of mobile phone, in a contractual way, but the positions differ according to the services. Within our department setting in charge of the young people in crises, the arguments are in favour of a use resounded with the mobile phone leaning on the peculiarities of the clinical situations.

1. Bydlowski, S., Corcos, M., Consoli, S. M., Paterniti, S., Laurier, C., Chambry, J., Loas, G., & Jeammet, P. (2002). Anhedonia and emotional awareness: facts and perspectives in eating disorders. *Annales De Medecine Interne, 153,* S52-S61.

**Abstract**

According to clinical reports, patients with eating disorders (EDs) are unable to differentiate and regulate emotional states. They are hypothesized alexithymic and lacking of emotional awareness. We investigate EDs capacity to experimente pleasure and levels of emotional awareness, in a comparative study. As expected, EDs show a global deficit of emotional functioning, with inability to identify and describe their own emotions, as well as an impairment in mentalising others' emotional experience, and an anhedonia. No relations between the duration of illness and the emotional dimensions were found. This trouble in regulation of emotions either follows the eating disorder and constitutes a lasting sequel, or appears to be a personality trait.

1. Candelori, C., Fassone, P., & Mancone, A. (1999). Attachment theory and eating disorders. *Psicoterapia e Istituzioni: Review of Psychoanalytical Methodological Research and Clinical Experiences, 6*(1-2), 51-64. Retrieved from <http://ovidsp.ovid.com/ovidweb.cgi?T=JS&PAGE=reference&D=psyc3&NEWS=N&AN=2001-18928-003>.

**Abstract**

Discusses the role of attachment theory in research on anorexia nervosa and bulimia. Emphasis is on the usefulness of (1) the Adult Attachment Interview (G. C. Kaplan and M. Main, 1985) in parent-child psychotherapy and (2) an adapted version, the Attachment Interview for Childhood and Adolescence (M. Ammaniti et al, 1990).

1. Carano, A., Totaro, E., De Berardis, D., Mancini, L., Faiella, F., Pontalti, I., & Mariani, G. (2011). Correlations between bodily dissatisfaction, alexithymia, and dissociation in eating disorders. *Giornale Italiano di Psicopatologia/Italian Journal of Psychopathology, 17*(2), 174-182. Retrieved from <http://ovidsp.ovid.com/ovidweb.cgi?T=JS&PAGE=reference&D=psyc10&NEWS=N&AN=2013-05331-003>.

**Abstract**

Objectives: This study investigated the relationships between body image, alexithymia and dissociative symptoms in a sample of patients with eating disorders (EDs).  Methods: Self-rating tests were filled-out by 107 patients with a DSM-IV eating disorder diagnosis (Diagnostic and Statistical Manual of Mental Disorders, 4th edition). Alexithymia has been assessed through the Toronto Alexithymia Scale (TAS-20), dissociative symptoms through the Dissociative Experiences Scale (DES), eating disorder symptoms were assessed through the Eating Disorder Inventory-2 (EDI-2), and beliefs about the body through the Body Shape Questionnaire (BSQ). Additional assessment comprised the Whiteley Index, the State-Trait Anxiety Inventory (STAI) and the Beck Depression Inventory (BDI).  Results: Our sample consisted of 37 patients with anorexia nervosa (AN), 38 with bulimia nervosa (BN), and 30 patients with binge eating disorder (BED). The prevalence of alexithymia in our sample was 18.1% (n = 19); people with alexithymia scored higher on all self-rating scales (Table III). Patients with AN and BED showed more dissociative symptoms (45.9 +/- 20.1; 44.3 +/- 20.1) compared to patients with BN, and patients with BED scored higher also on the BSQ (71.1 +/- 26.5) (Table II). Adult age, higher body dissatisfaction, alexithymia, depressive symptoms, and dissociative experiences are predictive of a higher risk for developing an ED according to the linear regression analysis (Table V).  Conclusions: There is evidence for an association between dissociative tendency and alexithymia in EDs. Dissociative tendency would represent a coping style to deal with a negative body image in patients with alexithymia. Furthermore, alexithymic people with a negative body image showed a stronger trend toward the development of an ED, compared to patients who score lower on the TAS-20. Finally, an association between more dissociative symptoms and an alexithymic trait could possibly suggest a body image disorder or a subsyndromal ED.

1. Carrel, M. (2002). The cry of the body: Refusal of the feminine in a post-adolescent anorexic. *La Psychiatrie de l'Enfant, 45*(2), 593-603. <https://doi.org/10.3917/psye.452.0593>

**Abstract**

Marina is a 19 yr old anorexic patient. Short and very thin, she seems to be barely out of childhood. Confronted with a severe depression and few free associations, I propose that she undertake individual psychodrama. This treatment, over a 6 yr period, will be recounted. Dreams of water and liquid, swimming pools or seas filled with sharks will be the object of numerous settings for her enactments. The importance of primary homosexuality and the rapprochement between her and myself, her female therapist, keeps us vigilant during the action. Her quest for feminine identification is both recurrent and poignant to the point where she chooses me in the later stages of therapy to play the role of boys who interest her. To reaffirm the acceptance of a unisex body and no longer an ambisexual, androgynous one, she verifies the integrity of her own body in the identical one belonging to her female therapist. Narcissistic failure, a precocious defect in the protective shield of the maternal relationship, too great a proximity with a father-mother, all of these things spill out over the limits. She is prone to bulimic binges followed by, vomiting to control all the orifices, all the outlets of her body. The post-adolescent period which she is beginning to accept still worries her.

1. Castro, U., Larroy, C., & Gomez, M. A. (2010). Cognitive behavioral and parental group intervention for adolescents in the treatment of bulimia nervosa. *Revista de Psicopatologia y Psicologia Clinica, 15*(1), 49-60. <https://doi.org/10.5944/rppc.vol.15.num.1.2010.4084>

**Abstract**

The objective was to evaluate the effectiveness of an intervention program for adolescents with bulimia composed of a cognitive-behavioral treatment for patients (12 sessions) and a psychoeducational group activity with their parents (12 sessions). The study was conducted with 21 adolescent patients, diagnosed with bulimia nervosa, of the Eating Disorders Unit at the Hospital del Nino Jesus (Madrid, Spain), and their parents. The control group consisted of 17 patients with similar characteristics, whose parents received no intervention. The results show that this form of intervention is highly effective in reducing the core symptoms of bulimia nervosa and associated symptoms, maintaining the result at one year follow up, and that it is more effective than the control group (consisting in treatment for patients only) as regards dropouts and relapses, finding some statistically significant differences in these two variables.

1. Chinello, A., Corlazzoli, G., Zappa, L. E., & Ricciardelli, P. (2021). Parents of women affected by an eating disorder: Differences in empathy but not alexithymia. Psicoterapia Cognitiva e Comportamentale, 27(3), 279–292.

**Abstract**

Empathy and alexithymia constitute complex and relevant constructs in anorexia (AN) and bulimia nervosa (BU), with contrasting evidences at the family level. This study aims to explore the possible difference in these constructs by comparing 44 parents with anorexia nervosa daughters, 13 parents with bulimia nervosa daughters and 64 control parents by administering the TAS-20, the EQ and the RME. Regarding facial emotion recognition and empathy, between-group comparisons showed higher accuracy in the control group. Additionally, the difference is greater when AN parents and control parents are compared. This may suggest the presence of a deficient cognitive mechanism regarding emotion recognition, associated to the duration of illness. Alternatively, no between-group differences emerged in alexithymic levels, highlighting how this construct may be partially independent from empathy-related processes, as evidenced by the «Self to Others Model of Empathy» (SOME). These findings show the necessity of emotion-focused cognitive interventions in AN/BU parents with an aim to restore emotion recognition of facial expressions and empathy, especially in parents with AN daughter exhibiting a longer duration of illness. Further studies are required, adopting other methods to measure these complex constructs.

1. Cygankiewicz, P., Solecka, D., Pilecki, M. W., & Jozefik, B. (2012). Predictors of symptomatic improvement in eating disorders. Preliminary analysis. *Psychiatria Polska, 46*(2), 201-212. Retrieved from <http://ovidsp.ovid.com/ovidweb.cgi?T=JS&PAGE=reference&D=psyc11&NEWS=N&AN=2012-33204-006>.

**Abstract**

AIM: The article discusses the preliminary results of a follow-up study carried out in 2009-2010 on former patients with a diagnosis of anorexia nervosa and bulimia nervosa, first seen in 2001-2004 at the Department of Child and Adolescent Psychiatry, the Jagiellonian University Medical College in Krakow. At that time, they had been taking part in a research project, whose aim was to define the relationships among the psychopathological picture of eating disorders, self-image and family relations and also the influence of socio-cultural factors. The aim of the current study is to attempt to define factors influencing the course and prognosis of eating disorders in the studied group. METHOD: Results from the Eating Disorder Inventory Questionnaire (EDI) and the Polish version of Family Assessment Measure (KOR) in the first study were juxtaposed with the clinical state and parameters of psychosocial functioning of the studied women assessed on the basis of the follow-up study. RESULTS: In the studied group, 13 girls suffered from anorexia nervosa--restricting type, 6 from anorexia nervosa binge-eating/purging type, and 6 from bulimia. In the studied group, there was complete symptomatic improvement in 12 persons (48%), subclinical symptoms continued to be observed in 9 persons (36%), and 4 persons (16%) met full diagnostic criteria for eating disorders. The most favourable course was observed in the group with a diagnosis of anorexia nervosa restricting type. The least favourable was observed in the group with a diagnosis of bulimia. Results. In the studied group, 13 girls suffered from anorexia nervosa - restricting type, 6 from anorexia nervosa binge-eating/purging type, and 6 from bulimia. In the studied group, there was complete symptomatic improvement in 12 persons (48%), subclinical symptoms continued to be observed in 9 persons (36%), and 4 persons (16%) met full diagnostic criteria for eating disorders. The most favourable course was observed in the group with a diagnosis of anorexia nervosa restricting type. The least favourable was observed in the group with a diagnosis of bulimia. CONCLUSIONS: The occurrence of symptoms of binging and purging turned out to be a negative prognostic factor in the whole group of patients suffering from eating disorders. A smaller starting declared intensity of aspiring to slimness and level of dissatisfaction with own body by patients measured with the EDI, and satisfaction in the area of communication and emotional commitment on the part of the mother (of the patient) in her relationship with her husband (the father of the patient) measured with the KOR were positive prognostic factors. The significant overrepresentation of patients with a diagnosis of restrictive anorexia in the group of persons with clinical improvement may have an influence on the obtained results.

1. da Silva, G. A., Ximenes, R. C. C., Pinto, T. C. C., D'Arc de Souza Cintra, J., dos Santos, A. V., & do Nascimento, V. S. (2018). Consumption of dietary substances and their association with risk of eating disorders in college students. *Jornal Brasileiro de Psiquiatria, 67*(4) 239-246. <https://doi.org/10.1590/0047-2085000000211>

**Abstract**

Objective: to evaluate the consumption of weight loss formulations and their possible association with risk of eating disorders (ED) in university students of health courses of different socioeconomic levels. Methods: A cross-sectional epidemiological study was carried out with 276 university students enrolled in four health courses. To obtain the data, three self-applied instruments were used: the Eating Attitudes Test (EAT-26), the Bulimic Investigatory Test of Edinburgh (BITE) and to investigate the consumption of weight-loss formulations a questionnaire prepared by the research team was used. For the data analysis, the chi-square test was applied, adopting the level of significance of 5%. Results: Twenty-one university students presented a risk of ED by the EAT-26 scale, corresponding to 7.6% of the respondents. The frequency of use of weight loss formulations was 7.2%. There was a significant association (p < 0.001) between the use of weight loss formulations and the presence of risk for ED (33.3%), with a very high percentage when compared to the percentage of non-ED respondents who were using medication (5.1%). Conclusions: The consumption of dietary formulations was associated with both the presence of risk for ED, on the EAT-26 and BITE scales, and on socioeconomic levels, especially for income class C.

1. da Silva Leal, G. V., Philippi, S. T., Polacow, V. O., Cordas, T. A., & dos Santos Alvarenga, M. (2013). What is adolescents' eating disorder risk behavior? *Jornal Brasileiro de Psiquiatria, 62*(1), 62-75. <https://doi.org/10.1590/S0047-20852013000100009>

**Abstract**

Objective: To characterize eating disorder (ED) risk behavior and its prevalence among adolescents in a review of national and international literature. Methods: We carried out an integrative review on PubMed (U.S. National Library of Medicine), Lilacs and SciELO, using descriptors related to "eating disorder risk behavior". Articles published in the last 10 years, in Portuguese, Spanish and English, and specifically with adolescents were selected. The nomenclature and instruments used to assess eating disorder risk behaviors and its frequency were evaluated and analyzed in 76 articles. Results: Several terms to assess ED risk behavior were found; questionnaires and scales were used, mainly EAT-26 or 40 and BITE. The prevalence of risk ranged from 0.24% to 58.4%. Conclusion: Different names and instruments are used to assess ED risk behavior among adolescents, and there is a wide variation in the prevalence. Standardization of terms and evaluation methodology might enhance a better comparison of epidemiological studies in different locations.

1. Daga, G. A., Quaranta, M., Notaro, G., Urani, C., Amianto, F., & Fassino, S. (2011). Family therapy and eating disorders in young female patients: State of the art. *Giornale Italiano di Psicopatologia / Italian Journal of Psychopathology, 17*(1), 40-47. Retrieved from <http://ovidsp.ovid.com/ovidweb.cgi?T=JS&PAGE=reference&D=psyc10&NEWS=N&AN=2011-08527-012>.

**Abstract**

Background and objective: Eating disorders essentially affect females in their adolescence and early adulthood. Lifetime prevalence of anorexia nervosa and bulimia nervosa are respectively 0.9% and 1.5% 1. Due to the complexity of their pathogenesis, multidisciplinary approaches are required. Family Therapy is commonly recommended and implemented with adolescents with eating disorder. Nevertheless, a general agreement on the most effective approaches has not yet been reached. This article aims to review the main scientific evidence concerning family-based therapy in eating disorders, to clarify its efficacy and pertinence in improving patients' symptoms and family relationship.  Methods: We searched the Medline database using eating disorders, anorexia nervosa, bulimia nervosa and family therapy as general keywords. Two authors separately reviewed papers satisfying all inclusion criteria. Patients were female adolescents and young adults aged between 12 and 22 years. Studies involving adult patients older than 22 years were excluded to minimize sample heterogeneity. Moreover, we excluded case reports, commentaries, studies published before 1987 or those with unclear methodology, as well as studies with less than 20 patients.  Results: 18 studies respected inclusion criteria and were included in this analysis. Available evidence regarded anorexia nervosa almost exclusively; reviewed studies showed higher efficacy of family-therapy in some subgroups, i.e., younger patients and those with a short duration of illness. Evidence on bulimia nervosa is still lacking. Many controversies exist, especially on the role of family- based therapy  during the early phases of treatment. Peculiar personality traits preclude its applicability and mandate for other types of intervention such as Cognitive Behaviour Therapy or Individual Supportive Therapy. 
Conclusions: Data concerning the efficacy of family-based therapy applied to anorexia nervosa seem to be promising, but future research will need to focus on larger samples and possibly, on high-quality designs. With respect to bulimia nervosa, evidence is still poor and controversial. Current data do not enable us to formalize a correct analysis. Future research should clarify any specific role of family-based therapy in treating this complex disorder.

1. Dahlbender, R. W., Buchheim, A., & Doering, S. (2004). OPD and AAI: integrative diagnostics of structure, conflict and attachment representation. PTT*: Personlichkeitsstorungen Theorie und Therapie, 8*(4), 251-261. Retrieved from <http://ovidsp.ovid.com/ovidweb.cgi?T=JS&PAGE=reference&D=psyc5&NEWS=N&AN=2005-01214-005>.

**Abstract**

This paper deals with the question, in how far attachment theory and psychoanalysis can benefit from each other with regard to their clinical interview techniques. Three groups of questions concerning representation of early relationship, early separation, and early loss were taken from the Adult Attachment Interview (AAI) and integrated into the interview of the Operationalized Psychodynamic Diagnostics (OPD). One female patient suffering from bulimia, cannabis use, and borderline personality disorder was interviewed with both, the AAI and the modified OPD interview. It could be demonstrated that the OPD interview can benefit from the integration of the evaluation of early representations of attachment, particularly for the assessment of psychodynamic conflicts and psychic structure. Moreover, the analysis of transference and countertransference can be confounded from an attachment theory point of view.

1. De Alda, I. O., Espina, A., & Ortego, M. A. (2006). A study about personality, anxiety and depression in parents of patients with an eating disorder. *Clinica y Salud, 17*(2), 151-170. Retrieved from <http://ovidsp.ovid.com/ovidweb.cgi?T=JS&PAGE=reference&D=psyc6&NEWS=N&AN=2007-01401-002>.

**Abstract**

Objective: To study personality, anxiety and depression in parents of patients in an eating disorder sample (ED) compared with a control group.  Method: 100 families (consisting of father, mother and daughter) with a daughter suffering from ED [DSM-IV] (32 with restrictive anorexia nervosa, 31 with bulimic anorexia nervosa and 37 with bulimia nervosa, and a control group of 90 families) were evaluated with the Eysenck Personality Questionnaire, the Beck Depression Inventory and the Self-Rating Anxiety Scale.  Results: Parents in the experimental group presented higher scores in BDI, SAS, neuroticism and psychoticism. Discussion: these results could be associated with the family burden, even though it could be pre-existent to the daughter's pathology and play some etiological role. ED treatment could be improved through interventions on family burden and parents' personality.

1. De Amusquibar, A. M. G., & De Simone, C. J. (2005). Childhood trauma. Its effects. *Anales de Psiquiatria, 21*(7), 307-311. Retrieved from <http://ovidsp.ovid.com/ovidweb.cgi?T=JS&PAGE=reference&D=psyc5&NEWS=N&AN=2006-04937-001>.

**Abstract**

Objective: To evaluate the frequency of Childhood Sexual Abuse (CSA) and Family Violence (FV) in patients that consulted the Eating Disorders Department at the Hospital Italiano of Buenos Aires. 
Method: 440 patients were interviewed and diagnosed according to the DSM IV criteria. 
Results: 340 patients met diagnostic criteria for eating disorders constituted group I. The remaining 100 patients, group II, had other psychiatric disorders. In group I, 13.2% had a history of CSA and 23.5% of V.F. Both were more frequent in bulimic patients. In group II the frequency of CSA was 5% and FV 14%. Conclusions: The childhood traumatic experiences may increase the vulnerability to suffer from eating disorders, especially bulimia nervosa.

1. de Bernart, R., Ferrara, M., & Pecchioli, S. (2019). The importance of being siblings. *Terapia Familiare: Rivista Interdisciplinare di Ricerca e Intervento Relazionale, 119*, 9-20. <https://doi.org/10.3280/TF2019-119002>

**Abstract**

The Siblings Sub-System is considered a very important resource for family therapy. The authors propose a relatively new kind of intervention focused on this Sub-System. After an overview of the literature on this subject, and particularly on Siblings in the "Normal Family", the Authors propose two models of intervention for "High Access Siblings " and "Low Access Siblings ". Both are based on the building of a "Mind of the Siblings ", which is described. Results, limits and counterindications are discussed.

1. de Oliveira, J., Figueredo, L., & Cordas, T. A. (2019). Prevalence of eating disorders risk behavior and "low-carb" diet in university students. *Jornal Brasileiro de Psiquiatria, 68*(4), 183-190. <https://doi.org/10.1590/0047-2085000000245>

**Abstract**

Objectives: To identify the presence of binge eating associated or not with compensatory practices in low-carb dieters.  Methods: Binge Eating Scale (BES) and Hay Questionnaire were used in order to assess the frequency of binge eating and compensatory practices, in addition to a frequency questionnaire for the consumption of chocolate, bread and rice. A scale of 1-8 points assessed the carbohydrate restriction intensity, and participants were divided into groups (i) low-carb diet and (ii) control. Comparisons between groups were part of the analysis, as well as correlations between variables of interest per diet group (p < 0.05). Results: Participants were a total of 853 university students, in which 75.97% were women with an average of 22.04 years old (SD = 3.33) and an average BMI of 23.56 kg/m2, (SD = 4.38). From the aforementioned total, 214 had a low-carb diet, and 639 did not. The prevalence of a high score suggestive of binge eating without compensatory practices was 17.94% (n = 153), while the presence of binge eating associated with compensation was 2.23% (n = 19). As for the diet group, 35.05% (n = 75) also performed intermittent fasting. The diet group reached higher values for ECAP and BMI, and lower for frequency of consumption of rice and bread. Furthermore, ECAP scores correlated positively with chocolate consumption (r = + 0.14; p = 0.0377) and BMI values (r = + 0.19; p = 0.0042), whereas carbohydrate restriction showed negative correlation with chocolate consumption (r = - 0.13; p = 0.041); French bread (r = - 0.20; p = 0.0024) and rice (r = - 0.36; p = <0.0001).  Conclusions: We highlight the high prevalence of diet practice (25.09%), and the higher levels of binge eating in this group, as well as the lower consumption of rice and bread compared to those who did not diet.

1. de Vito, E. (2013). The link between ambivalent-preoccupied mode of attachment and narcissistic vulnerability in adolescence. *Praxis der Kinderpsychologie und Kinderpsychiatrie, 62*(10), 748-757. <https://doi.org/10.13109/prkk.2013.62.10.748>

**Abstract**

Attachment research has deepened our understanding of the essential continuities and discontinuities of psychological development from childhood to adolescence and to adulthood. This paper considers the relevance of ambivalent-preoccupied attachment, one of the two types of insecure attachment, for understanding emotional vulnerability during adolescence. How current social and economic conditions have exacerbated the effects of ambivalent-preoccupied attachment in the attainment of the developmental tasks of adolescence is considered. By describing three clinical vignettes clinical recommendations are given.

1. Delaunay, A. L., Gerardin, P., & Godart, N. (2019). State of knowledge and perspectives of eating disorders adolescents’ management in day hospital program: Where do we stand in France? *Neuropsychiatrie de l'Enfance et de l'Adolescence, 67*(4), 203-212. <https://doi.org/10.1016/j.neurenf.2019.03.003>

**Abstract**

Objective of the study: To establish an international literature review and to make a state of knowledge of patient care in day-care hospital program settings for adolescents with eating disorders by a national survey: existing care, modalities and care programs. Method: The bibliographic search was done according to the Prisma method (23 initial articles and 9 selected articles published between 2003 and 2015). A national survey was sent via e-mail. All 2015 FFAB indexed structures have been contacted. In overall, 62 structures claimed to have a day-care hospital program treating adolescent eating disorder patients. The existing literature promotes the day-care hospital program benefits: global patient evaluation, care grading, intensive outpatient treatment avoiding continued inpatient treatment, post-inpatient treatment handover to step up outpatient treatment, inpatient treatment preparation. Results: Fifteen of the 62 finally replies stated they offered a day-care hospital program for adolescent eating disorder patients (only five with a specialized patient care for eating disorders), representing 77 beds in day-care hospital settings. All of them treat patients with anorexia nervosa, 73,3% bulimia nervosa and 26,7% hyperphagia. Patient age varies between 6 and 40 years. The patient care weekly frequency varies from 1 to 5 half-days per week. A total of 26,7% follows an intensive care of 5 days per week. Conclusion: It appears crucial that, in the coming years, France has to set up dedicated day-care hospitals for adolescents, such as those found in Canada, Germany and Great Britain. With a real efficacy, while limiting costs, they allow to smoothly adapt patient care with efficiency for patients while preserving their social insertion.

1. Delourmel, C. (2005). The analytic third party and the self-reflexive power of the psyche: On a number of risks involved. *Revue Francaise de Psychanalyse, 69*(3), 809-826. <https://doi.org/10.3917/rfp.693.0809>

**Abstract**

The author proposes to consider the problematic of the analytic third party with regard to the risks pertaining to the self-reflexive power of the ego, of which the Greek myth of Perseus gives a happy metaphorical account, and the story of Bluebeard a more traumatic version. The effacement of this psychic capacity is considered to be at the source of a recourse to defence, the prototype of which is the process of "primary fetishism" described by Michel Fain with regard to merycism. This last bastion against the threat of disorganisation underlies Mme A...'s bulimic behaviour, the narcissistic organisation of Mr M...'s discourse in the session, and Mme C...'s somnambulism, as well as the very complex processes used by Glen Gould to play the piano.

1. Demir, T., Eralp Demir, D., Kayaalp, M. L., & Buyukkal, B. (1998). Sociodemographic, familial, and personal characteristics of adolescents with eating disorders. *Turk Psikiyatri Dergisi, 9*(4), 257-264. Retrieved from <http://ovidsp.ovid.com/ovidweb.cgi?T=JS&PAGE=reference&D=psyc3&NEWS=N&AN=1999-00123-002>.

**Abstract**

The aim of this study was to detect cases of eating disorders among high school students and then to compare the cases with normal controls on sociodemographic, familial, and personal variables. The Eating Attitudes Test (EAT) was given to 9th graders from 4 high schools in Istanbul, Turkey. The students who scored equal to or above the cut-off point were interviewed using Structured Clinical Interview for %DSM-III-R%. The case group included students who had any eating disorder diagnosis. The Ss in the control group had test scores below the cut-off point. A detailed questionnaire prepared by the investigators was filled for all the students, and for each student, weight and height were measured. Of 944 students who filled out the EAT correctly, 101 scored 30 or above. After diagnostic interview, 25 Ss had an eating disorder diagnosis, of whom 17 had the diagnosis of bulimia nervosa and 8 binge eating disorder. Significant differences were found between the case and control groups with regard to mean weight, reluctance to go to school, running away from home, and having menstrual irregularities. Results suggest that eating disorders are not infrequent among adolescents.

1. Denda, K., Sunami, T., Inoue, S., Sasaki, F., Sasaki, Y., Asakura, S., & Koyama, T. (2002). Clinical Study of Early-Onset Eating Disorders. *Japanese Journal of Child and Adolescent Psychiatry, 43*(1), 30-56. Retrieved from <http://ovidsp.ovid.com/ovidweb.cgi?T=JS&PAGE=reference&D=psyc4&NEWS=N&AN=2004-12009-003>.

**Abstract**

The clinical features, comorbidity, clinical courses, and general outcomes of early-onset eating disorders were examined. Forty-four children and adolescents (4 boys, 40 girls; 9 to 14 years old) who had eating disorders, based on DSM-IV criteria, and had been referred to the Department of Psychiatry, Hokkaido University Hospital between 1991 and 2000, were studied. The characteristic features of early-onset eating disorders were 1. Of those studied, such disorders accounted for 12.5%. Mean age at onset was 13.3 +/- 1.4 (means+/-SD) years. 2. Of the 44 patients, 22 (50%) were classified as having anorexia nervosa of the restricting type (AN-R); 7 (15.9%) anorexia of the binge eating type (AN-BP); 8 (18.2%) bulimia nervosa of the purging type (BNNP). 3. Those with AN-R formed two groups; "non-dieter" and "dieter". Of the early-onset eating disorder patients, "non-dieter" AN-R ones had the most characteristic symptoms. They suffered loss of appetite and subsequent involuntary weight loss as a result of psychological or physical stresses at home or at school, but did not intentionally restrict their food intake. They expressed no fear of obsesses nor a desire for thinness. They did not talk about being handicapped by their body image and offered much resistance to treatment for gaining weight. The "dieter" AN-R patients and AN-BP, BN-P, and BN-NP ones had symptoms similar to those of adolescent and adult patients. 4. There was a high rate (72.7%) of comorbidity in the early-onset eating disorder patients. Specifically, the rates of comorbidity were 56.8% with mood disorders, 15.9% with obsessive compulsive disorder, and 6.8% with schizophrenia. 5. General outcomes for the patients with early-onset eating disorders were excellent 36.8%, good 28.9%, fairly good 13.2%, and poor 21.1%. The diagnostic problems, psychopathology of each type of disorder, characteristic features of comorbidity, and general outcomes are discussed.

1. Diaz, J. M. M, Gasga, L. M., Rodriguez, E. M., Rayon, G. L. A., Aguilar, X. L., & Fernandez, M. R. (1999). Risk factors in eating disorders. *Revista Mexicana de Psicologia, 16*(1), 37-46. Retrieved from <http://ovidsp.ovid.com/ovidweb.cgi?T=JS&PAGE=reference&D=psyc3&NEWS=N&AN=2001-00679-004>.

**Abstract**

The purpose of this study is to evaluate the occurrence of anorexia and bulimia nervosa in Mexican students, to evaluate the relationship between risk factors and eating disorders, and to verify if there are differences between normal Ss and Ss with eating disorders with regard to the risk factors. A total of 524 college students (mean age 19.3 yrs), were administered the Eating Attitudes Test (EAT-40), Test of Bulimia (BULIT) and the Eating Disorders Inventory (EDI). Those students who exceeded the cut-off point of the EAT-40 and BULIT scales were classified as having an eating disorder. 27 college students were diagnosed as anorexic while 17 were diagnosed as bulimic. Risk factors were identified from some of the scales of the EDI. The authors found significant differences between the eating disorders sample and the control group sample when risk factors were assessed. Dissatisfaction with body image, social pressure, a desire for thinness, ineffectiveness, interpersonal distrust, and perfectionism were correlated significantly with EAT-40 scores.

1. Diebel-Braune, E. (1991). Some critical reflections on the status of the psychoanalytic discussion of bulimia. *Zeitschrift fur Psychosomatische Medizin und Psychoanalyse, 37*(3), 292-304. Retrieved from <http://ovidsp.ovid.com/ovidweb.cgi?T=JS&PAGE=reference&D=psyc3&NEWS=N&AN=1992-85188-001>.

**Abstract**

Observes that, between 1941 and 1988, the majority of studies on bulimia emphasized the pathogenic significance of early and the earliest psychological development stages in mother-child interaction. In contrast, only a few works discuss conflict elaboration at later psychosexual development stages. Clinical research and the results of a study conducted by the present author with 40 female bulimia sufferers and 28 female medical students of the same age (controls) militate against emphasis being placed exclusively on early-childhood factors and early childhood genesis of adolescent bulimia. Adolescent psychosexual development problems, and phenomena typical of female adolescence as psychogenic factors in bulimia, are highlighted.

1. Dimova, N., Kolibas, E., Novotny, V., Havlickova, E., Obuch, I., & Balcarova, K. (1999). Manifestations of anxiety in patients with eating disorders and their changes during citalopram treatment. *Ceska a Slovenska Psychiatrie, 95*(7), 462-471. Retrieved from <http://ovidsp.ovid.com/ovidweb.cgi?T=JS&PAGE=reference&D=psyc3&NEWS=N&AN=1999-01305-001>.

**Abstract**

Clinical characteristics of eating disorders are associated with a high psychiatric comorbidity. In a pilot study of anxiety disorders in Ss with anorexia nervosa and bulimia, the authors investigated the incidence of anxiety disorders in 1st-grade relatives as well as in the Ss. The group comprised 35 women (aged 13-28 yrs). Data on anxiety disorders in 1st-grade relatives were found in 5 Ss (14.29% of the group). In 4 cases an obsessive-compulsive disorder was involved and in 1 case a separation anxiety disorder was found. An anxiety disorder preceded the development of an eating disorder in 20 Ss. An obsessive-compulsive disorder was found in 14 Ss (40% of the group), and a phobic disorder was found in 6 Ss (15.4% of the group). Results found actual anxiety disorders or isolated anxiety symptoms were in 27 Ss (77.1%). Ss completed questionnaires measuring anxiety and anxiousness. During the 28 day Citalopram treatment, the Ss' anxiety was reduced.

1. Dumet, N., & Juteau, A. (2013). "To kill a fetus, to kill a body?" When anorexia and bulimia tell the story of an intra-uterine destructiveness. *Cliniques mediterraneennes: Psychanalyse et Psychopathologie Freudiennes, 87*, 143-158. <https://doi.org/10.3917/cm.087.0143>

**Abstract**

The authors of this article propose to study some eating disorders regarding the impact of antenatal experiences, and more specifically the traumatic and destructive experiences occurring during womb life. Based on two clinical case studies, the authors show how bulimia and anorexia, far from showing a lack of psychoaffective expression in those wifes, could rather represent the embodied and expressive trace of fetal traumas related to maternal destructiveness (real and\or symbolic). Having not been able to be metabolized by the maternal object (or, more generally, its environment), those traumatic experiences, inherent of the mother-child bonding process, appear to have been engramed in those subjects on the same historical way they were actually lived, in its corporal and sensorial way. In this respect, the eating disorders could bring up to date and make perceptible those primitive experiences. Anorexia and bulimia would not be related to a mortiferous repetition compulsion but to a symbolization compulsion, sustained on the corporal way, preliminary or premise to any other modality of symbolization or subjectivation.

1. Dunker, K. L. L., dos Santos Alvarenga, M., & Alves, V. P. O. (2009). Eating disorders and pregnancy-A review. *Jornal Brasileiro de Psiquiatria, 58*(1), 60-68. <https://doi.org/10.1590/S0047-20852009000100010>

**Abstract**

Objective: The objective of this study was to show the impact of eating disorders on reproductive functions, pregnancy and puerperium problems, and difficulties in feeding her babies. Methods: Review of literature of last 28 years in MedLine and Lilacs database. The keywords used were anorexia nervosa, bulimia nervosa, eating disorders and pregnancy. Results: Review and case studies, and research made with eating disorders pregnant patients demonstrate that there are an association between eating disorders and many pregnancy complications on birth, to the child with an increased risk of morbidity after the birth and some implications on food behavior of the child. Conclusions: It was noticed a need for specialized treatment, especially before the pregnancy, regarding eating habits and worries about weight and body shape, especially for women that present inadequate weight gain, hyperemesis gravidarum, pica and other eating problems.

1. Duo, I., Sepulveda, A. R., Leon, M., Pastor, J., & Lopez, M. P. (2015). Bulimic Behaviour Observation Questionnaire (COBU): Developing an instrument for caregivers of patients with bulimia nervosa. *Revista de Psicopatologia y Psicologia Clinica, 20*(2), 135-149. <https://doi.org/10.5944/rppc.vol.20.num.2.2015.15168>

**Abstract**

The aim was to develop and evaluate the psychometric properties of the Bulimic Behaviour Observation Questionnaire (COBU), related to the symptoms observed in bulimia nervosa (BN) by caregivers. The sample consisted of 162 caregivers of patients with BN (n = 126), anorexia nervosa (AN) (n = 26) and binge eating disorder (n = 10). Exploratory analysis of the COBU revealed three factors: Overeating observation (alpha = 0.77), Restriction Interval Observation (alpha = 0.76) and Impulsivity Observation (alpha = 0.62). The questionnaire showed good convergent validity with the ABOS scale for carers (rho = 0.58), p < .01) and the BITE scale for patients (rho = 0.40, p < 0.01). The questionnaire discriminates between clinical diagnoses, except for the Impulsivity Observation subscale. It is recommended for use in the assessment by the family and to assist in recognizing and assessing changes in the symptoms.

1. Edelstein, E. L. (1996). Object creation and personification in eating disorders. *Revue de Neuropsychologie, 12*(1), 45-56. Retrieved from <http://ovidsp.ovid.com/ovidweb.cgi?T=JS&PAGE=reference&D=psyc3&NEWS=N&AN=1996-00736-004>.

**Abstract**

Describes eating disorders as expressions of a disturbed personal sphere, which cause a permanent conflict between yearning for a symbiotic closeness and fear of being abandoned. The author illustrates his theme with the clinical case of an anorexic-bulimic patient, who contributed much to his understanding of object relations and their difficulties. Eating and not-eating are considered to be tantamount to having or not-having a relationship, or to the difference between life and death. The body becomes a transitional object in the struggle for autonomy. The personification of food represents an object choice that lessens, or even avoids, the closeness-distance conflict. This happens in the effort of trying to reconcile internal and external reality.

1. Edit, C., Peter, C., & Robert, U. (2011). Body dissatisfaction and its risk factors; Hungarian adaptation of Body Shape Questionnaire Short Form 14. *Psychiatria Hungarica, 26*(4), 241-249. Retrieved from <http://ovidsp.ovid.com/ovidweb.cgi?T=JS&PAGE=reference&D=psyc10&NEWS=N&AN=2011-26231-004>.

**Abstract**

Introduction: Body dissatisfaction has severe impact on obesity related psychological distress and other mental health problems. The main goals of this study were to present psychometric analysis of the Hungarian version of Body Shape Questionnaire Short Form 14 (BSQ-14), and to explore the risk factors of body dissatisfaction in participants of a health promotion program. Methods: The study design is a cross sectional analysis nested in a prospective study. Our respondents (n = 377; 61% women; the mean age = 38.8 SD = 9.58, BMI = 27.0 kg/m2 SD = 5.52) participate in a countrywide worksite health promotion program targeting weight problems and physical inactivity. Measures included the Hungarian version of Body Shape Questionnaire Short Form 14, Eating Disorder Inventory Bulimia sub-scale, body-weight, body-high, self-reported lifetime jojo effect (weight cycling), weight satisfaction. Results: The one-factor measurement model is supported by confirmatory factor analysis with inclusion two error covariances. Internal consistency of this scale is excellent (Chronbach alpha = 0.95). A multivariate analysis revealed that gender (female), actual weight, bulimic tendency/binge eating, self-reported lifetime jojo effect and higher educational attainment are associated with higher body dissatisfaction. Conclusions: The reliability and construct validity of the Hungarian version of Body Shape Questionnaire Short Form 14 are supported in the present study. Measuring body dissatisfaction might provide a good tool to identify high risk groups and risk factors for psychological distress and to develop optimal intervention programs.

1. Eicke-Spengler, M. (2002). On anality in women. *Zeitschrift fur Psychoanalytische Theorie und Praxis / Journal for Psychoanalytical Theory and Practice, 17*(1-2), 23-30. <https://doi.org/10.15534/ZPTP/2002/1-2/3>

**Abstract**

The author sketches the path of the female child's psychic development into a woman from the point of view of the fate of anality. Beginning with the invisibility and untouchability of the female genitals and with the fact that sensations of the inner body become conscious for the first time during the anal phase and are being differently experienced by girl and boy, the author argues that this turns into a crucial point for developing a stable and well-integrated bodily self. She points to differences regarding the fate of anality during those levels of development where they first appear as a topic. The essay especially focusses on influences of defense maneuvers related to anality during pregnancy and in bulimic patients.

1. Eizaguirre, A. E., de Cabezon, M. A. O. S., de Alda, I. O., de Apellaniz, M., & Mendez, A. A. (2001). Alexithymia, family and eating disorders. *Anales de Psicologia, 17*(1), 139-149. Retrieved from <http://ovidsp.ovid.com/ovidweb.cgi?T=JS&PAGE=reference&D=psyc3&NEWS=N&AN=2001-05708-009>.

**Abstract**

The objective of this study was to contrast the differences in the level of alexithymia (as measured by the Toronto Alexithymia Scale-20, TAS-20) among families with a daughter with eating disorders (ED) and a control group. 64 women with DSM-IV-diagnosed ED (26 with restrictive anorexia nervosa (RAN), 14 with bulimic anorexia nervosa (AN-BN), 29 with bulimia nervosa (BN), and a control group, 55 women and their parents, with similar sociodemographic characteristics) were evaluated with the TAS-20 and the Eating Attitude Test (EAT). The daughters with ED and their parents presented bigger scores on the TAS-20. The daughters' alexithymia was associated to their parents'. It is concluded that alexithymia could be a characteristic of families with an ED, but it cannot be confirmed whether it is a trait, or a state due to the ED.

1. Escandon-Nagel, N., Dada, G., Grau, A., Soriano, J., & Feixas, G. (2017). Eating disorder patients’ evolution three years after intake in day hospital. *Revista Argentina de Clinica Psicologica, 26*(1), 59-69. Retrieved from <http://ovidsp.ovid.com/ovidweb.cgi?T=JS&PAGE=reference&D=psyc16&NEWS=N&AN=2017-22466-005>.

**Abstract**

Course of eating disorders (ED) in patients treated in Day Hospital is analyzed by evaluating 24 women at baseline and approximately 3 years after initial assessment, in several variables: personality factors, motivation, psychological symptoms, severity, self-esteem, self-construction, construction of parental figures and polarization. Clinical variables of the history of the disorder are also included. 70.8% of the patients completed treatment and, 3 years after intake, 47.06% relapsed. The following factors seem to be associated with negative course: severity of ED, high scores on relapse (Transtheoretical Model of Change) and opening to experience, long period between onset of symptoms and first treatment, self-perception of fatness body image? low self-esteem prior to ED, and negative perception of their mother.

1. Esparza, M. L., Sauceda Garcia, J. M., & Ulloa Flores, R. E. (2011). Family characteristics and eating disorders in a sample of female adolescent inpatients in a psychiatric hospital. *Salud Mental, 34*(3), 203-210. Retrieved from <http://ovidsp.ovid.com/ovidweb.cgi?T=JS&PAGE=reference&D=psyc10&NEWS=N&AN=2011-15366-003>.

**Abstract**

The aim of this study is to describe the frequency of the eating disorders as well as eating disorder not otherwise specified in a sample of inpatient female adolescents; and to establish the relationship that functioning and quality of the family environment hold with the severity and/or characteristics of the eating psychopathology. The study included a group of 36 female adolescents hospitalized due to any type of psychopathology in the Children's Psychiatric Hospital Dr. Juan N. Navarro. From the 36 patients included, 39% presented an eating disorder, 42% presented only body dissatisfaction and 19% of the sample was free of eating psychopathology. Eating disorders represent an important cause of morbidity in adolescent female inpatients; likewise, the patients were more frequently diagnosed with eating disorders not otherwise specified than with anorexia nervosa and bulimia nervosa. In the sample recruited for the present study, we found that the eating disorders not otherwise specified represented 56% of the total of eating disorders, making early detection necessary for the beginning of treatments directed to avoid the evolution to severe forms.

1. Espina Eizaguirre, A., Ortego Saenz de Cabezon, M. A., & Ochoa de Alda Martinez de Apellaniz, I. (2000). A controlled trial of family interventions in eating disorders. *Anales de Psiquiatria, 16*(8), 322-336. Retrieved from <http://ovidsp.ovid.com/ovidweb.cgi?T=JS&PAGE=reference&D=psyc3&NEWS=N&AN=2000-14408-002>.

**Abstract**

Studied the efficacy of systemic family therapy and parental support group plus group therapy in 71 female adolescents and adults (aged 14-33 yrs) with eating disorders in Spain. Data on sociodemographic variables and clinical and psychological symptoms were obtained by semistructured interview and questionnaire. The Eating Attitudes Test (D. M. Garner and P. E. Garfinkel, 1979), the Body Shape Questionnaire (P. J. Cooper et al, 1987), the Bulimia Investigation Test Edinburgh (M. Henderson and C. P. Freeman, 1987), Eating Disorder Inventory, the Anorexic Behavior Observation Scale (W. Vandereycken, 1992), and the Diagnostic and Statistical Manual of Mental Disorders-IV (DSM-IV) were used. The results indicate that both systemic family therapy and parental support plus group therapy are effective in reducing eating disorder symptoms at 1 yr, but systemic family therapy shows greater reductions in eating disorder symptoms in patients with bulimia nervosa than parental support plus group therapy. Implications for improving treatment for eating disorders are discussed

1. Fichter, M. M. (2005). Anorexic and bulimic eating disorders. *Der Nervenarzt, 76*(9), 1141-1153. <https://doi.org/10.1007/s00115-005-1976-8>

**Abstract**

Anorexic and bulimic eating disorders today are rather frequent in adolescent girls and young women of developed industrial countries. News media frequently report such patients, and lay people are interested. For scientists, it is not easy to explain the etiology and pathophysiology of these eating disorders. Clinically, treatment is a challenge. General risk factors for the development of anorexic and bulimic eating disorders are (1) female gender, (2) adolescence, and (3) living in an industrial country. Special risk factors are (1) obesity or mental disorders (eating, depression, substance use), (2) premorbid characteristics (early menarche, childhood obesity, anxiety disorder, low self-esteem, and perfectionism), and (3) premorbid stresses. Biological and sociocultural factors and personally threatening experiences all play a role in the etiology. Especially in early phases of the illness, affected patients do not appear to suffer, are reluctant to admit symptoms, and may avoid necessary treatment. Progress has recently been made in the understanding and treatment of anorexic and bulimic eating disorders.

1. Fortes, L. S., Cipriani, F. M., Paes, S. T., Coelho, F. D., & Ferreira, M. E. C. (2016). Relationship between mood and risk eating behaviors to eating disorders in adolescents. Jornal Brasileiro de Psiquiatria, *65*(2) 155-160. <https://doi.org/10.1590/0047-2085000000117>

**Abstract**

Objective: To analyze the relationship between mood and risk eating behaviors to eating disorders (ED) in female adolescents. Methods: Three hundred and ninety-seven adolescents aged between 12 and 17 years participated. We used the subscales of the Eating Attitudes Test (EAT-26) to assess the risk eating behaviors to ED. We used the Brunel Mood Scale (BRUMS) to assess negative mood (anxiety, strain, depression, mental confusion, anger and fatigue). Led to Binary Logistic Regression and Multivariate Analysis of Covariance to analyze the data. Results: We revealed a statistically significant association between negative mood and risk eating behaviors to ED (X^2^ = 25.71; Wald = 31.92; p = 0.001). We identified differences in Diet subscale scores of adolescents with high and low negative mood [F (1, 396) = 13.40; p = 0.04], which was not found for the Bulimia and Food Preoccupation subscale [F (1, 396) = 1.69; p = 0.09] and Selfcontrol Oral [F (1, 396) = 0.95; p = 0.17]. Conclusion: The risk eating behaviors to ED were related to negative mood.

1. Franco, K., de Jesus Diaz, F., Lopez-Espinoza, A., del Consuelo Escoto, M., & Camacho, E. J. (2013). Predictors of risk for eating disorders in women. *Terapia Psicologica, 31*(2), 219-225. <https://doi.org/10.4067/S0718-48082013000200008>

**Abstract**

The purpose of this research was to examine the role of body composition, body dissatisfaction, and thinness model on the risk of developing eating disorders (ED). The sample comprised 289 female students who answered the Eating Attitudes Test, the Bulimic Investigatory Test, Edinburgh, and the Questionnaire on Influences on Body Shape Model. The body composition was assessed by electrical impedance. The results showed that the risk for having an ED was found in 9.69% of women, and the percentage was high among normal weight women, especially women who had excessive body fat. The body dissatisfaction predicted the risk for anorexia nervosa, and the interaction between body dissatisfaction, influence of advertisement and body mass index predicted the risk for bulimia nervosa. These findings suggest that the role of body dissatisfaction was relevant to predict the risk for ED.

1. Frost, U., Strack, M., Kronmuller, K. T., Stefini, A., Horn, H., Winkelmann, K., Bents, H., Rutz, U., & Reich, G. (2014). Shame and family relations in bulimia. Mediation analysis of eating disorder symptoms and psychiatric burden. *Psychotherapeut, 59*(1), 38-45. <https://doi.org/10.1007/s00278-013-1010-8>

**Abstract**

Background: Empirical studies show a relationship between family factors and disturbed eating behavior. Feelings of shame are associated with a higher level of eating disorder symptoms and with family relations perceived as being dysfunctional. Thus, shame can be understood as a mediator of the relationship between dysfunctional family relations and eating disorder symptoms. Material and methods: For 69 female patients, including 55 with bulimia nervosa and 14 with eating disorder not otherwise specified (EDNOS) between 14 and 22 years of age, who participated in a comparative study of psychotherapy outcome, eating disorder symptoms (EDI, EDE-Q), general psychiatric symptoms severity (SCL-90R), level of shame (TESE-KJ) and perceived family relations (FB-A) were measured at the beginning of psychotherapy. Results: The higher the feeling of shame the more dysfunctional the perceived family relationships were and the more the eating disorders and general symptoms severity were reported. Shame was a partial mediator of the relationship between family functionality and symptoms. Discussion: Feelings of shame could originate in dysfunctional family relationships but could also evoke more negative perceptions of interpersonal relationships. The direction of causality could not be proven in the correlative design; nevertheless, shame contributes to an understanding of the mechanisms between dysfunctional family relationships and eating disorder symptoms.

1. Garrido, R, R., Garcia Garcia, J. J., Luaces Cubells, C., FashehYoussef, W., Pou Fernandez, J., & Alda Diez, J. A. (2004). Predictive factors of suicide attempt recidivism. *Revista de Psiquiatria Infanto-Juvenil, 21*(3), 149-170. Retrieved from <http://ovidsp.ovid.com/ovidweb.cgi?T=JS&PAGE=reference&D=psyc5&NEWS=N&AN=2005-01227-001>.

**Abstract**

Background: Suicide attempt is an important problem among teen population, being the most frequent psychiatric emergency in this age group. There is more suicide attempt repetition risk in teenagers with previous attempts, and this repetition rate ranges from 6%-15% per year. Objectives: The aims of this study are to determine the incidence of suicide attempt repetition and its possible predictive factors. Material and methods: A study about teenagers admitted by voluntary poisoning as a suicide attempt was developed in the Emergency Department of Sant Joan de Deu Hospital between January 1996 and December 1997. We carried out a prospective observation of these 43 patients between January 1998 and July 2000. Results: The mean age is 15.6 years, being more frequently the female sex (87%). 58.1% have been under previous psychiatric control. Among the causes of this observation the highlighted ones are: alimentary conduct disorder (9: 21%): 7 (16.3%) anorexia nervosa and 2 (4.7%) bulimia. Suicide attempt is repeated in 20 cases (46.51%). Suicide attempt number is 2 in 12 cases, 3 in 6 cases and 4 in 2 cases. No significant differences are observed between recidivist and non-recidivist groups regarding the analysed risk family factors: psychiatric background, suicide attempt, alcohol consumption or separated parents. There is a significant relation (p = 0.039) between the chronic pathology and the recidivist group: from 20 repeating patients, 4 (205) present an organic pathology, while none of the recidivist patients present it. There is a significant relation between previous psychiatric control and recidivism: 80% of the recidivists are under psychiatric control versus 39% of non-recidivists (p = 0.012). 66.7% from the alimentary conduct disorder patient group repeats (6/9). Comments: There is a high suicide attempt and alimentary conduct disorder repetition percentage. No differences are seen regarding family risk background between recidivist and non-recidivist groups. Differences are significant if psychiatric background and chronic organic pathology are analysed.

1. Agnes, G., Ildiko, A., & Amaryl, A. (2009). Dreams and imaginations in the therapy of eating disordered patients. *Psychiatria Hungarica, 24*(5), 320-327. Retrieved from <http://ovidsp.ovid.com/ovidweb.cgi?T=JS&PAGE=reference&D=psyc8&NEWS=N&AN=2010-00335-002>.

**Abstract**

Recently the integrative approach has been applied in the treatment of eating disorders with multicausal origin. In order to achieve long-term therapeutic effect, the psychodynamic interpretation is often needed and favoured as a part of the personal, multimodal therapeutic strategy. The present paper focuses on body image distortion as one of the most decisive and least influenceable symptom of the disorder. The symptom is interpreted along the body image distortion-body boundaries-self boundaries-autonomy line. With illustrative therapy details of dreams, imaginations, the authors aim to demonstrate the therapeutic use of dynamically oriented therapy and dynamically oriented hypnotherapy.

1. Gaudriault, P., Belandre, C., & Sellem, C. (2007). Bulimia in the Rorschach: Self and object representations in multivariate analysis. Pratiques *Psychologiques, 13*(1), 81-104. <https://doi.org/10.1016/j.prps.2006.07.006>

**Abstract**

The aim of this study was to examine self and object representation disorders defined by bulimic women. 113 bulimics and 115 non-bulimics, aged 18 to 58, both outpatient women, where assessed by the Rorschach test according to the french method. 14 topics were selected from their international psychopathological literature then translated into 14 Rorschach indices from the analysis of each protocol. Ten of the fourteen selected indices distinguish the two groups in a significant way. A multivariate analysis (AFCM) reveals in the bulimic group five dimensions corresponding to 63% of the inertia. If the first dimension appears to be related to general psychopathology, the four others are more specific to bulimic representation disorders.

1. Gerlinghoff, M. (1988). Disorders in the process of maturation in the psychosexual development of patients with anorexia nervosa. Schweizer *Archiv fur Neurologie, Neurochirurgie und Psychiatrie, 139*(5), 61-73. Retrieved from <http://ovidsp.ovid.com/ovidweb.cgi?T=JS&PAGE=reference&D=psyc3&NEWS=N&AN=1990-76619-001>.

**Abstract**

Studied the association among anorexia nervosa, sexual attitudes and behaviors, maturation disorders, and family factors among adolescent and adult females. Human subjects: 51 female adolescents and adults (aged 13-33 yrs) (anorexia nervosa and/or bulimia). The duration of anorexia nervosa with or without bulimia, engagement in various sexual behaviors, desire for and attitudes toward sexual behaviors, household atmosphere, type of sexual education, role of the mother and father in framing the developmental process, and the ages of the Ss' parents were examined.

1. Gil, M., Simoes, M. M., de Oliveira-Cardoso, E. A., Pessa, R. P., Leonidas, C., & dos Santos, M. A. (2022). Perception of family members of people with eating disorders about treatment: A meta-synthesis of the literature. Psicologia: Teoria e Pesquisa, 38. <https://doi.org/10.1590/0102.3772e38417.pt>

**Abstract**

Family relationships seem to have a relevant contribution to the etiopathogenesis of eating disorders (EDs), which makes the international treatment guidelines recommend family participation. This study aimed to synthesize and reinterpret the findings of primary qualitative studies about the perception of family of people with EDs about treatment. The search strategy was organized using the SPIDER tool, and the analyzes were carried out by two independent reviewers. From 1115 studies originally retrieved from six databases (CINAHL, LILACS, PsycINFO, PubMed, Scopus and Web of Science), 19 articles were selected. The results converge to the recognition that the family is part of the process and also needs to be considered as a care recipient, not just as a coadjuvant/companion for the patient. Since family dynamics have a relevant contribution in the development and maintenance of EDs, it is necessary to invest in the transformation of family relationships so that more effective and lasting gains in treatment can be obtained.

1. Goldstein, R. M. (1999). The pleasures outside word and the organic and addictive supplications. *Tropicos: Revista de Psicoanalisis, 7*(2), 185-196. Retrieved from <http://ovidsp.ovid.com/ovidweb.cgi?T=JS&PAGE=reference&D=psyc3&NEWS=N&AN=2000-12374-011>.

**Abstract**

In the causation of psychosomatics, and in some types of insidious drug addictions, the local symbolic Verwerfung (foreclosure or repudiation) of the Name of the Father, addressed by J. Lacan (and recognized by Freud and many other authors), is linked to two others modalities of the Verwerfung: the imaginary Verwerfung and the real Verwerfung. These can be discerned in the cure of psychotics and autistics and in the "craziness" of somatics, bulimics-anorexics, and addicts as a jouissance outside word or as one of the modalities of the jouissance of the other. In this article the author emphasizes the importance of the constitution of the invisible as one's own imaginary register-seat of the ego, the feeling of one's self, and of the imaginary foreclosure which make them explode. The basis on which this topic is developed has 2 fundamental axes: (1) the distinction between reversible "supleciones" and irreversible "suplencias" of efficiency in the achievement of some social bond or not for the subjective structure in the cure, and (2) the distinction between constituent Verwerjung and deconstituent Verwerfung. Finally, the author presents the clinical vignette of a 40-yr-old man to illustrate these clinical propositions.

1. Gonzalez-Macias, L., Romero, M., Rascon, M. L., & Caballero, A. (2013). Anorexia nervosa: Family experiences about the start of suffering, treatment, relapse and remission. *Salud Mental, 36*(1), 33-39. <https://doi.org/10.17711/SM.0185-3325.2013.005>

**Abstract**

Introduction Anorexia nervosa is a suffering entity from eating disorders and family has a relevant role in these disorders; a severe family dysfunction is a risk factor and an important maintainer factor. On the other hand, the family's participation in treatment increases the possibility of success. The aim of this paper is to contribute with the analysis of the form on which the subjects, who suffer from anorexia nervosa and their parents, coexist with their suffering, and share their story. Materials and methods: It is a qualitative research study that pretends to know about the family's experiences. The informants were selected according to a theoretical non probabilistic sample. Depth interviews were used and the material was analysed through a narrative analysis by thematic axis according to Kolher Riessman model. Results The presented family is formed by three members, mother, father and daughter, with anorexia nervosa. Presenting excessively restrictive behaviors oscillating between control and impulsivity. The mother turns her into her confident and the father is too strict and demanding. Concerning the treatment, it lasted approximately three years on impasse, the patient did not show any evolution. Discussion The four evolution moments of the suffering had several points of view according to each of the informants, in literature, the mother and daughter's symbiotic relationship and the peripheral relationship established with the father are mentioned, change was achieved in the family alliances and the change in the nature of their bonds was facilitated. Conclusions These findings are an initial step to know the experiences of each of the family members during the treatment and the expectations they have towards the remission of the suffering.

1. Guarin, M. R. (2009). Drug use, impulsivity, and trauma in patients with eating disorders: A triad of risks for complex comorbidities affecting the prognosis. *Revista Colombiana de Psiquiatria, 38*(3), 420-432. Retrieved from <http://ovidsp.ovid.com/ovidweb.cgi?T=JS&PAGE=reference&D=psyc8&NEWS=N&AN=2011-13716-002>.

**Abstract**

Introduction: Few studies have explored, in a clinical population of patients receiving outpatient treatment for eating disorders (ED), the variables drug abuse and dependency, history of trauma, and multi-impulsivity, and their impact on treatment compliance and prognosis. The objective of this study is to do so and analyse their impact on relapse frequency and early treatment dropout. Method: 578 male and female patients, who consecutively entered the outpatient program EQUILIBRIO in Bogota between June 1997 and December 2007 to be treated for their eating disorders, were studied using the DSM-IV Structured Clinical Interview (SCID 1).  Results: On entry, 201 patients presented with nervous anorexia; 220 with nervous bulimia; 121 with food gorging disorder; and 36 with incomplete pictures of anorexia or bulimia; 108 had drug-related problems (alcohol, marijuana, cocaine and other stimulants) at the time of entry to the program. Of the main group 111 periodically self-injured. Conclusion: The presence of drug abuse and trauma history is associated with a complex pattern of predominantly impulsive comorbidities, with a higher risk for suicide attempts, other parasuicidal behaviours, relapses or early treatment dropout. This situation makes it necessary to manage each comorbidity specifically.

1. Guedeney, A. (1995). The latest on merycism to the latest on early depression: A critical review of the literature and apsychopathological hypothesis. *La Psychiatrie de l'Enfant, 38*(1), 345-363. Retrieved from <http://ovidsp.ovid.com/ovidweb.cgi?T=JS&PAGE=reference&D=psyc3&NEWS=N&AN=1996-02350-006>.

**Abstract**

Merycism has always intrigued clinicians. A review of current literature reveals its characteristics. The auto-stimulation dimension appears important, whatever the underlying psychopathology of merycism. Its frequency during early childhood seems to have considerably diminished, even almost disappeared. Merycism continues to exist in the anorexic and bulimic young adult. The relationship of merycism in early childhood to depression and growth delay is analyzed.

1. Gueniche, K., Vibert, S., Ouallouche, C., Nataf, N., & Polak, M. (2016). Receiving a diagnosis of utero-vaginal agenesis. *Revue Adolescence, 34*(3), 525-538. <https://doi.org/10.3917/ado.097.0525>

**Abstract**

Referring to clinical data and projective testing results gleaned from medical and psychological studies open to patients suffering from utero-vaginal agenesis, our article focuses on the anorexic or bulimic reactions adolescents frequently present the diagnosis has been pronounced. Our reflections on access to feminine sexual life will be supported by a clinical case highlighting the psychical treatment involved in the constriction of the psychical-corporal inner life.

1. Guenter, R. (2000). Female obesity. *Analise Psicologica, 18*(1), 59-70. Retrieved from <http://ovidsp.ovid.com/ovidweb.cgi?T=JS&PAGE=reference&D=psyc3&NEWS=N&AN=2000-00045-002>.

**Abstract**

Studied the psychodynamics of the personality of obese women in relationship to earlier parent-child interactions. The Rorschach and a diagnostic interview were administered to 40 premenopausal obese women (without endocrine dysfunction) of low, average, and high SES in Portugal. 6 Ss were bulimic. The development and course of Ss' oral phase were assessed and were correlated with depressive feelings. 32 Ss exhibited obsessive-compulsive behavior. Ss generally demonstrated (1) a fixation on the oral phase of mental development, (2) conflicts regarding sexuality, and (3) projective identification with their mother. The results indicate a possible link between depression and demanding, narcissistic mothers who undervalued their daughters.

1. Guiducci, V., Bizzi, F., Ferro, A., & Cavanna, D. (2018). Affective dysregulation, attachment disorganization and eating disorders: Individual and familial risk factors. *Maltrattamento e Abuso All'Infanzia: Rivista Interdisciplinare, 20*(2), 65-85. <https://doi.org/10.3280/MAL2018-002005>

**Abstract**

Attachment working models have been interpreted such as affective regulation/dysregulation patterns, whose disruption is associated with eating disorders. The purpose of this research work was to investigate attachment models and alexithymia in a sample of young 50 outpatients with anorexia and bulimia, in a subsample of parents and in a control group. The following instruments were administrated: Adult Attachment Interview, Toronto Alexithymia Scale, Observer Alexithymia Scale, SCID-I. The results show an over-representation of dismissing and disorganized attachment in the clinical sample. ED subjects show a significantly higher level of alexithymia than the control group. Daughters and parents tend to attribute to each other alexithymic characteristics. Alexithymia could be considered as a symptom of a family that is not able to promote the development of affect mentalization.

1. Guilbaud, O., Corcos, M., Chambry, J., Paterniti, S., Flament, M., & Jeammet, P. (1999). Psychosomatic vulnerability and eating disorders. *Annales Medico-Psychologiques, 157*(6), 390-401. Retrieved from <http://ovidsp.ovid.com/ovidweb.cgi?T=JS&PAGE=reference&D=psyc3&NEWS=N&AN=1999-11457-001>.

**Abstract**

Conducted a retrospective study of 64 female patients (aged 18-25 yrs) with anorexia nervosa (32 Ss) and bulimia (32 Ss) and 74 matched control Ss in France to determine the incidence of 4 psychosomatic ailments: asthma, migraine, eczema, and urticaria. Ss were administered the Hospital Anxiety and Depression Scale (A. S. Zigmond and R. P. Snaith, 1983) and the 20-item version of the Toronto Alexithymia Scale (R. M. Bagby et al, 1992). The results showed no significant difference in the psychosomatic vulnerability of anorexic and bulimic patients vs control Ss. Migraine was more frequent in bulimic patients, and eczema was more common in anorexic patients. Alexithymia was encountered more often in patients vs control Ss, but nonthymo-dependent alexithymia was not predictive of psychosomatic vulnerability except for migraine.

1. Guilbaud, O., Corcos, M., Chambry, J., Paterniti, S., & Jeammet, P. (2000). Alexithymia and depression in eating disorders. *L'Encephale: Revue de psychiatrie clinique biologique et therapeutique, 26*(5), 1-6. Retrieved from <http://ovidsp.ovid.com/ovidweb.cgi?T=JS&PAGE=reference&D=psyc3&NEWS=N&AN=2000-12714-001>.

**Abstract**

Patients suffering from eating disorder show elevated rates of alexithymia and depression. We compared alexithymia and depression ratings for nonhospitalized women meeting DSM IV criteria for anorexia nervosa (n = 32) and bulimia nervosa (n = 32) to healthy women (n = 74). Alexithymia was evaluated by the Toronto Alexithymia Scale (TAS-20) and depression by the Hospital Anxiety and Depression Scale (HAD). We found that TAS and HAD scores were significantly higher in anorexic compared to bulimic patients, although alexithymia and depression, as evaluated were significantly and positively correlated with each other (r=0,53, p=0,001). Finally, a logistic regression with alexithymia and depression as independent variables showed a strong correlation between the HAD ratings and anorexia, but no correlations between TAS score and the eating disorder subgroups. In eating disorder patients, alexithymia, as evaluated by the Toronto Alexithymia Scale, seems to exhibit a thyme-dependent component which could be secondary to concurrent depression. Through recent studies and results of our research, we analyse and give several interpretations which may explain this correlation between alexithymia and depression.

1. Hagenah, U., Blume, V., Flacke-Redanz, M., & Herpertz-Dahlmann, B. (2003). Psychoeducation for parents of children with eating disorder. *Zeitschrift fur Kinder- und Jugendpsychiatrie und Psychotherapie, 31*(1), 51-58. <https://doi.org/10.1024/1422-4917.31.1.51>

**Abstract**

Psychoeducation is a well-established component of cognitive-behavioral therapy in adult patients with eating disorders. This paper describes a group model of psychoeducation that has been offered by our department for the past two years to parents of adolescent patients with anorexia and bulimia nervosa. According to their own reports, parents appreciate this means of support to help them cope with their child's illness.

1. Hagenah, U., & Vloet, T. (2005). Parent psychoeducation groups in the treatment of adolescents with eating disorders. *Praxis der Kinderpsychologie und Kinderpsychiatrie, 54*(4), 303-317. Retrieved from <http://ovidsp.ovid.com/ovidweb.cgi?T=JS&PAGE=reference&D=psyc5&NEWS=N&AN=2005-06413-004>.

**Abstract**

Parents of adolescents with eating disorders show high levels of emotional distress, which may contribute to less functional coping with the illness of their child. In our department a psycho-educational group program is been offered to the parents of 153 children and adolescents with anorexia and bulimia nervosa. According to their own reports parents are highly interested in gaining information about the eating disorder and appreciate this means of support to help them to cope with the illness of their child.

1. Harms, A. (2012). On the search - The therapeutic dealing with the emotion of maternal responsibility in the long-term treatment of a traumatized adolescent. *Selbstpsychologie: Europaische Zeitschrift fur psychoanalytische Therapie und Forschung / Self Psychology: European Journal for Psychoanalytic Therapy and Research, 13*(47), 29-43. Retrieved from <http://ovidsp.ovid.com/ovidweb.cgi?T=JS&PAGE=reference&D=psyc11&NEWS=N&AN=2012-19460-002>.

**Abstract**

This paper presents the multiphasic and at this point unfinished treatment initially anorectic and later bulimic adolescent which has been stretching over a number of years. Growing up in an abusive environment the young girl developed a false self <<. The central matter discussed in this paper is the thin line which may be running between the spontaneous motherly responses of the analyst and the parental self-object aspects of the transference as it unfolds in the therapeutic relationship. What are the boundaries and what are the preconditions of the therapeutic relationship at times when the adolescent - who not only needs to develop a more stable sense of a true << self but has free herself from abusive relationships - not only seeks in her analyst the the protection, the comfort, and support she never had when the analyst also wants support her in her efforts to reach more independence and an increasing degree determination? Throughout the years, the patient needed both: the real protection, real support, and the specific analytic approach addressing her developmental needs.

1. Henning, K., & Straus, B. (2003). Personal Child-Related Motives and Attitudes in Females with Eating Disorders: Indicators for Gender Role Conflicts? *PPmP: Psychotherapie Psychosomatik Medizinische Psychologie, 53*(1), 23-28. <https://doi.org/10.1055/s-2003-36482>

**Abstract**

In the context of the etiology of eating disorders it is common to discuss emotional conflicts related to the identification with the female gender role. Based upon this theoretical background this study was designed to examine the attitudes of 46 females suffering from anorexia or bulimia towards their wish to have children. It was assumed that the personal evaluation of motherhood and attitudes towards own children might reflect one important aspect of the identification with the female gender role. The results of this study are based on the "Leipziger Fragebogen zu Kinderwunschmotiven" and the "Leipziger Fragebogen zu Einstellungen zum Kinderwunsch" by Stobel-Richter and Brahler [16] and suggest that females suffering from eating disorders are more likely to refuse traditional concepts of the female gender role, especially a traditional concept of motherhood.

1. Hernandez Munoz, S., & Camarena-Medellin, B. (2014). Role of serotonin transporter gene in eating disorders. *Revista Colombiana de Psiquiatria, 43*(4), 218-224. <https://doi.org/10.1016/j.rcp.2014.08.003>

**Abstract**

Background: The serotoninergic system has been implicated in mood and appetite regulation, and the serotonin transporter gene *(SLC6A4)* is a commonly studied candidate gene for eating disorders. However, most studies have focused on a single polymorphism (5-HTTLPR) in *SLC6A4*. Objective: We present the studies published on the association between eating disorders (ED) and 5-HTTLPR polymorphism in anorexia nervosa (AN), bulimia nervosa (BN), and eating disorders not otherwise specified (EDNOS). Method: Search of databases: MEDLINE, ISI, and PubMed for *SLC6A4* and ED. Conclusions: From a review of 37 original articles, it was suggested that carriers of S allele is a risk factor for eating disorders, especially for AN. However, BN did not show any association. Also, BMI, impulsivity, anxiety, depression, and age of onset have been associated with S allele in ED patients.

1. Herpertz-Dahlmann, B. (1988). Affective disorders in the families of patients with anorexia nervosa. *Zeitschrift fur Kinder-und Jugendpsychiatrie, 16*(1), 14-19. Retrieved from <http://ovidsp.ovid.com/ovidweb.cgi?T=JS&PAGE=reference&D=psyc3&NEWS=N&AN=1990-71565-001>.

**Abstract**

Studied the prevalence of psychiatric disorders in families of patients with anorexia nervosa. Human subjects: 45 male and female West German children, adolescents, and adults (10-21 yrs) (anorexia nervosa). 38 male and female West German children, adolescents, and adults (neurotic and affective disorders) (control group). The parents of all Ss were interviewed with regard to the occurrence of affective disorders, eating disorders, and alcohol abuse among 1st-, 2nd-, and 3rd-degree relatives. Total family risk values were calculated for each S, and intergroup differences were analyzed.

1. Hers, D., Derely, M., Devos, M., Vandenbosch, M., & Roussaux, J. P. (1988). Anorexia nervosa and drug addiction vs. "toxicorexia." *Acta Psychiatrica Belgica, 88*(2), 93-104. Retrieved from <http://ovidsp.ovid.com/ovidweb.cgi?T=JS&PAGE=reference&D=psyc3&NEWS=N&AN=1990-75795-001>.

**Abstract**

Discusses the course and familial characteristics of anorexia nervosa and drug addiction. The term "toxicorexia" is proposed to describe anorexia or bulimia associated with drug or alcohol abuse. The approach to treatment is considered.

1. Hettinger, R., & Kachele, H. (2006). A retrospective account of a former bulimic patient. *International Journal of Psychotherapy, 10*(3), 62-71. Retrieved from <http://ovidsp.ovid.com/ovidweb.cgi?T=JS&PAGE=reference&D=psyc6&NEWS=N&AN=2007-02679-007>.

**Abstract**

Instrumentation in psychotherapy research requires psychometric instruments of increased refinement and audio-taped sessions or video-recordings; it may furthermore require the formalized use of the clinically trained perspective. Research interviewing, free interviewing by researchers recorded and evaluated by a group could serve as an intermediary between the clinicians and the structured techniques of the researchers. The basic idea consists of providing a therapy analogue situation to adequately evaluate the subjective experiences and recordable effects of psychotherapy. The following is a former patient's retro report on her life experiences that ultimately led her to a successful psychoanalytic treatment. The open, extended interview was conducted by a female research psychologist (RH) and two female psychology students3. Before the interview the therapist (HK) only provided basic information to the research team; for this report he made some comments that are handled by way of concurrent footnoting. The patient's motives for agreeing to a research team evaluation may be due to the fact that she had experienced a series of "unsuccessful" therapeutic endeavours ("looking back in anger") which was then followed by a satisfying therapeutic, psychoanalytic treatment with the senior author of this report4. Her description of her personal development points to a marked marital tension in the relationship of her parents with a strong, demanding and seducing father and a weak, caretaking mother that provided little help to the patient in developing a positive female identity. The narratives about her various treatments convey the impressions that she responded negatively to pronounced, demanding therapeutic attitudes which did not allow her to create her subjective space for correcting developmental fixations. A positive therapeutic experience was created by using a patient-oriented psychoanalytic technique (Thoma & Kachele 1987) that allowed for her deep-seated mistrust to be alleviated.

1. Hilbert, A., & Czaja, J. (2011). Behavioral indicators of loss of control over eating: Bite size and bite velocity. *Praxis der Kinderpsychologie und Kinderpsychiatrie, 60*(4), 270-284. <https://doi.org/10.13109/prkk.2011.60.4.270>

**Abstract**

Behavioral Indicators of Loss of Control over Eating: Bite Size and Bite Velocity Loss of control (LOG) over eating is a common and psychopathologically relevant experience in childhood. The current study aimed at evaluating bite size and bite velocity as behavioral indicators of LOG eating in an experimental test meal study with a variation of mood. Children with or without LOG eating (N = 120,8-13 years) consumed a parent-child test meal and a child-only meal consisting of snack food, following induction of negative mood. Bite size and bite velocity were determined through behavioral observation, food intake was measured, and sense of LOG and mood were rated. Children with LOC eating did not show greater bite size and bite velocity than children without LOC eating. Bite size of children with LOC eating was increased in negative mood and decreased in neutral mood. Greater bite size and bite velocity predicted greater food intake at test meal and snack eating and greater LOC over eating at snack eating, however, without an intervening influence of negative mood. Bite size was significantly associated with greater body weight of child and parent. Bite size and bite velocity were not significantly associated with eating disorder psychopathology and varying levels of LOC symptoms. The evidence as to whether bite size and bite velocity are suited as behavioral indicators of LOC eating is not clear-cut. Further research on behavioral indicators of childhood LOC eating is warranted.

1. Hirsch, M. (1989). Own body and food as representations of objects in anorexia and bulimia. *Praxis der Kinderpsychologie und Kinderpsychiatrie, 38*(3), 78-82. Retrieved from <http://ovidsp.ovid.com/ovidweb.cgi?T=JS&PAGE=reference&D=psyc3&NEWS=N&AN=1990-77847-001>.

**Abstract**

Discusses ways in which the body and food function as symbols for good and bad maternal objects in anorexia and bulimia. In anorexia, the underweight body is perceived as the good-mother object ("like mother"). In bulimia, food before binging becomes the good-mother object, to be incorporated, and food after eating becomes the bad-mother object, to be purged.

1. Hubin-Gayte, M. (2011). Eating disorders and motherhood: Analysis of forums on Internet. *Annales Medico-Psychologiques, 169*(10), 615-620. <https://doi.org/10.1016/j.amp.2010.06.001>

**Abstract**

Eating disorders affect about 5–7% of women of child-bearing age. Very little is known about the impact of pregnancy on women with anorexia or bulimia nervosa. Some research reported a generalized feeling of well-being during pregnancy once symptoms have passed. Other researches highlighted the fact that during pregnancy anxiety may worsen, they also found that women did not improve but their symptoms persisted or deteriorated. This lack of consensus is explained by the variety of methodologies used in these studies. Most studies in the literature are difficult to interpret as they rely on small samples, with heterogeneous, poorly described cases, and often use retrospective outcome measures or reports of ED symptoms. In our opinion, a very current tool appears to be missing from that plurality of approaches. Indeed, the use of Internet offers the possibility to women with eating disorders to share their experiences or their thoughts about pregnancy, and motherhood more generally. It is important to underline that not very many women inform their obstetrician or health care professional about their ED and a lot of them minimize or lie about their eating behaviours to family and friends. Aim: The aim of this study is to show that those testimonies may constitute a new corpus of knowledge of interest to the psychologist clinician, but also to every researcher preoccupied with this field of research. Method: In order to do so we searched websites, using a very often used search engine, by typing keywords such as “anorexia and pregnancy”. Then, we consulted the first five sites put on the front page in which took place this type of group discussions. So, without any choice a priori, we printed the exchanges between the women. Our testimonies correspond to the interventions of 60 Internet users. Sample: The sample presents the following characteristics: they are 26 and half years old on average. Among them, approximately 1/3 are already pregnant, 1/3 already have children and 1/3 wish to become pregnant. A lot of them experience fertility problems, and have a hormonal treatment. It is also necessary to note the number of women who say they benefit from a psychological care (16.6%). Methodology: We used the methodology of the analysis of contents. It consisted in reading all the corpus of testimonies to analyse what was written, to transcribe it, and to report the main ideas or developed subjects. For that purpose, we elaborated a “thematic scale” to establish the different contents, the facts, the ideas, which are present in the testimonies; literary and theoretical knowledge about the subject was also needed. What we exactly wanted to show was the relevance of the clinical approach of psychoanalytical orientation in the analysis of the forums on Internet. In this article, we wanted to develop the main themes, which appear on the most regular basis in the testimonies of these women. Results: In our results, we found and exposed six themes: “For the love of a man”, “The wish to become pregnant”, “The singular relation with my body”, “To contain a baby or kilos too many”, “Pregnancy outcome”, “The intergenerational transmission of the symptoms of the mother to the child”. It was important to connect these testimonies with what we know on the subject through literature and different theories. The results of our investigation show the necessity to consider the richness of its testimonies. It is thus important that the psychologists neither ignore nor disdain this mode of communication, but on the contrary, take it into account in their psychopathologic approach of TCA. We can discuss the benefit of prevention during pregnancy, which is a period of vulnerability but of change too.

1. Ilona, S., & Sandor, G. (2005). The role of mother-child relationship in development of self-image, dysfunctional attitudes and coping strategies. *Psychiatria Hungarica, 20*(2), 126-140. Retrieved from <http://ovidsp.ovid.com/ovidweb.cgi?T=JS&PAGE=reference&D=psyc5&NEWS=N&AN=2005-08153-005>.

**Abstract**

Introduction: Our study consisted of two interrelated work stages. In stage one, we set the goal to investigate coping styles and dysfunctional attitudes in patients with eating disorders. In stage two we studied the relationship between coping styles of the patients and mother-child interactional patterns, considering the role of communication in development of stress handling and problem management. Purposes: 1. Revealing characteristic coping patterns of the patients, comparing the coping styles to control subjects; 2. identifying dysfunctional attitudes in patients' flunking; 3. testing the hypothesis about existence of relationship between dysfunctional attitudes and coping; 4. finding out whether any dysfunctional attitudes or coping styles can be related to processes in mother-child communication. Subjects: There were 28 participants in the first stage (9 subjects with anorexia nervosa, 10 subjects with bulimia nervosa and 9 control subjects). In stage two, 20 subjects (5 patients with anorexia, 5 with bulimia together with their mothers) were studied. Methods: We used two questionnaires: Anxiety Handling Questionnaire and the Beck-Weissman Dysfunctional Attitudes Scale (DAS), shortened version. The Consensus Rorschach was administered in order to reveal Interactional dynamics. In summary, 1769 code units were analysed. 
Results: Our subjects, primarily subjects with bulimia nervosa, used more self-punishing coping compared to control subjects. Dysfunctional attitudes were found to have an effect on coping style. The present results indicate some possibilities for explanation how fragile self-image, dysfunctional attitudes and self-punishing coping patterns may develop in the patients as a possible effect of mother-child interaction dynamic.

1. Israel, L., & et al. (1968). Preliminary reflections concerning a psychopathological approach in mental anorexia. *Annales Medico-Psychologiques, 2*(2), 284. Retrieved from <http://ovidsp.ovid.com/ovidweb.cgi?T=JS&PAGE=reference&D=psyc2&NEWS=N&AN=1969-13120-001>.

**Abstract**

Presents clarifications regarding the symptomatology and the family structure of 20 clinic patients treated by psychotherapy: (1) anorexia was preceded by an anorectic and bulimic alternation; (2) amenorrhea, the major symptom, may have appeared long before the loss of weight; and (3) seduction of or by the father preceded the symptomatic explosion. It is believed that the anorexia breaks out when the relationship formed between the daughter and the father is threatened with dissolution, which "allows a regression to an infantile and asexual Eden."

1. Izydorczyk, B. (2010). Psychotherapy based on object relation with object and psychodynamic attitude to treatment of eating disorders. *Psychiatria Polska, 44*(5), 677-691. Retrieved from <http://ovidsp.ovid.com/ovidweb.cgi?T=JS&PAGE=reference&D=psyc9&NEWS=N&AN=2011-25023-005>.

**Abstract**

The article presents a proposal of integrating the basic concepts of object relations theory with the psychodramatic techniques by Moreno in treatment of eating disorders (anorexia and bulimia nervosa). The author pays attention to the common elements and also on the differences in psychopathology of eating disorder from a medical perspective, psychoanalytic and psychodynamic paradigm and moreover psychodrama by Moreno. Moreover, she points to common elements and the possibility of applying psychodramatic and psychodynamic therapeutic techniques in individual and group psychotherapy in persons with eating disorders. The presented attempt of integrating psychotherapy based on relation with object and psychodramatic techniques by Moreno can enlarge the repertoire of therapeutic techniques which intensify the recovery process in the group of persons with eating disorders (anorexia and bulimia nervosa).

1. Jaite, C., Pfeiffer, E., Lehmkuhl, U., & Salbach-Andrae, H. (2013). Childhood abuse in adolescents with anorexia nervosa compared to a psychiatric and a healthy control group. *Zeitschrift fur Kinder- und Jugendpsychiatrie und Psychotherapie, 41*(2) 99-108. <https://doi.org/10.1024/1422-4917/a000217>

**Abstract**

Objectives: Some studies suggest that both early sexual and early physical abuse are non-specific risk factors for the later development of eating disorders (EDs). However, only little is known about the role of emotional abuse in EDs. Methods: The sample included 77 inpatients with Anorexia nervosa (AN-R: n = 50; AN-BP: n = 27), 26 psychiatric control participants and 44 healthy control participants, all of whom were females. The diagnosis of AN and the diagnosis of psychiatric control participants were confirmed by structured interviews (SIAB-EX, [Fichter & Quadflieg, 1999](https://econtent.hogrefe.com/doi/10.1024/1422-4917/a000217#c16); CIDI-DIA-X, [Wittchen & Pfister, 1997](https://econtent.hogrefe.com/doi/10.1024/1422-4917/a000217#c48)). Childhood traumatization was assessed by a self-report questionnaire [Childhood Trauma Questionnaire (CTQ) German Version, Krischer & Sevecke, 2011]. Results: The results indicated there were higher rates of sexual and physical abuse as well as physical and emotional neglect in patients with AN-BP than in patients with AN-R and in healthy control participants, with no significant differences between patients with AN-R and control participants. Furthermore, patients with AN-BP had significantly higher rates on the CTQ subscale “emotional abuse” than patients with AN-R, psychiatric control participants and healthy control participants. Conclusions: Future studies should investigate whether emotional abuse is specific to adolescents with AN-BP compared to adolescents with other psychiatric disorders.

1. Janout, V., Kollarova, H., & Nemeckova, P. (2001). Risk factors in eating disorders: Crossectional epidemiological survey. *Ceska a Slovenska Psychiatrie, 97*(7), 350-355. Retrieved from <http://ovidsp.ovid.com/ovidweb.cgi?T=JS&PAGE=reference&D=psyc3&NEWS=N&AN=2001-09717-002>.

**Abstract**

Examined risk factors of anorexia nervosa and bulimia among 8 groups of girls (students of cosmetics school, school for health workers, high school and medical faculty, models, dancers, figure skaters and top gymnasts). The standardized EAT questionnaire and a special questionnaire for general attitudes were used. In 12% of the respondents, results representing a potential risk for development of these eating disorders were found. In spite of the fact that groups were not entirely homogeneous, top gymnasts and figure skaters were revealed as the most risky groups, followed by models, dancers and students of the cosmetics school. Students of high school, school for health workers and medical faculty had a lower risk.

1. Jeammet, P. (1997). Narcissistic and object-related dysregulations in bulimia. *Psyche: Zeitschrift fur Psychoanalyse und ihre Anwendungen, 51*(1), 1-32. Retrieved from <http://ovidsp.ovid.com/ovidweb.cgi?T=JS&PAGE=reference&D=psyc3&NEWS=N&AN=1997-90031-001>.

**Abstract**

Notes that bulimia represents a challenge for psychoanalysts, since it defies the application of standard procedures designed to illuminate psychic processes. Instead, the analyst is confronted with evident acting-out. The author argues that overeagerness to understand may actually blind the analyst to the nosological traits specific to bulimia. Therefore, Jeammet suggests that this acting-out be regarded not as a defense, but as an attempt at communication that cannot be articulated in any other way. From the acting-out perspective, it is possible to proceed in stages and explore the psychodynamics and the unconscious conflict constellation of the bulimic patient. This process must be pursued until it reveals the specifically-narcissistic and object-related dysregulation which the analyst is trying to confront.

1. Jozefik, B. (2008). Attachment theory and eating disorders-Theoretical and empirical issues. *Psychiatria Polska, 42*(2), 157-166. Retrieved from <http://ovidsp.ovid.com/ovidweb.cgi?T=JS&PAGE=reference&D=psyc7&NEWS=N&AN=2011-25228-001>.

**Abstract**

The paper presents the attachment theory in relation to eating disorders. In the first part, the classic concepts of anorexia and bulimia nervosa are discussed taking into account assumptions of Bowlby's and his followers' model. In the second part, empirical data on anorexia and bulimia nervosa and attachment patterns are presented. The importance of methodological issues is stressed regarding the attachment model particularly in eating disorders. In the conclusion significant findings correlation of attachment patterns and eating disorders are indicated.

1. Jozefik, B., Iniewicz, G., & Ulasinska, R. (2010). Attachment patterns, self-esteem, gender schema in anorexia and bulimia nervosa. *Psychiatria Polska, 44*(5)665-676. Retrieved from <http://ovidsp.ovid.com/ovidweb.cgi?T=JS&PAGE=reference&D=psyc9&NEWS=N&AN=2011-25023-004>.

**Abstract**

Aim: The aim of the study was comparison of perception of attachment patterns between adolescent girls suffering from anorexia nervosa (restrictive type), bulimia nervosa and healthy peers. Moreover, we tried to find the differences between the groups in self-esteem and identification with the culture-defined sex role. 
Method: We examined 40 patients with anorexia, 32 with bulimia and 63 girls from the comparison group. Three questionnaires were used: Parental Bonding Instrument, The Culture-Free Self-Esteem Inventory and Psychological Sex Inventory. Results: Results indicate that the parents' emotional commitment in relations with daughters from the clinical sample is weaker, and they are controlled by parents more than those from the control group. In all groups, the mothers' emotional commitment correlates positively with daughters' social self-esteem. In the anorectic group, fathers' control correlates negatively with daughters' identification with the culture-defined feminine role. In the bulimic group, fathers' emotional commitment correlates positively with self-esteem as well as daughters' identification with the culture-defined masculine role. Conclusions: The results concerning the bonds between the ED patients and their parents indicating dysfunctions of the bonds are generally consistent with the data from literature. Additionally, they show the relationship between quality of the bonds between the patients and parents and its influence on development of self-evaluation and a sense of femineity/masculinity in anorectic and bulimic girls.

1. Kaganski, I., & Remy, B. (1989). Family, clinical, and therapeutic aspects of eating disorders. *Confrontations Psychiatriques, 22*(31), 203-232. Retrieved from <http://ovidsp.ovid.com/ovidweb.cgi?T=JS&PAGE=reference&D=psyc3&NEWS=N&AN=1991-72491-001>.

**Abstract**

Discusses family characteristics and parent-child relations in anorexia nervosa and bulimia during adolescence, combining psychoanalytic and family-systems perspectives. Case materials are presented to illustrate the family dynamics of eating disorders, and 2 therapeutic modalities-parent groups and family systems therapy-are described. The importance of a therapeutic approach that is flexible, inventive, and sensitive to each family's unique situation is emphasized.

1. Kaneko, K. (2002). Dissociative Disorder with Psychotic Symptoms Caused by Physical Abuse. *Japanese Journal of Child and Adolescent Psychiatry, 43*(1), 57-63. Retrieved from <http://ovidsp.ovid.com/ovidweb.cgi?T=JS&PAGE=reference&D=psyc4&NEWS=N&AN=2004-12009-004>.

**Abstract**

The case of a 19-year-old girl with psychotic symptoms of bulimia, conversion, and depression, whose father had chronically physically abused her, is reported. The diagnosis was dissociative disorder. Supportive therapy is very useful for treating this type of patient. In traumatic disorders there is the risk of overemphasizing the trauma. As the patient's sense of responsibility is likely to be low, it may be difficult for them to overcome their disorders. The classification "traumatic disorders" provides a new diagnostic method for this type of disorder. In terms of treatment, however, it is most important that the strength of the ego rather than the trauma be addressed.

1. Karwautz, A., Haidvogl, M., Wagner, G., Nobis, G., Wöber-Bingöl, C., & Friedrich, M. H. (2002). Subjective family image in anorexia nervosa and bulimia nervosa in adolescence: A controlled study. *Z Kinder Jugendpsychiatr Psychother, 30*(4), 251-9.

**Abstract**

Objectives: The family environments of patients with eating disorders have been studied extensively in recent decades. The "Subjective Family Image Test" is an instrument developed especially to measure differential perceptions by family members. Assessments of subjective family image in families of adolescents by means of this test have been carried out in only a few samples. Methods: We aimed first to investigate subjective perceptions by adolescents of their family relations in a larger clinical sample of female adolescents (n = 118) suffering from anorexia nervosa of either subtype or from bulimia nervosa and to compare these perceptions with those of healthy controls (n = 96). Second, we investigated intra-familial differences in perception. Results: The main findings were that bulimia nervosa patients perceived lower individual autonomy and lower emotional connectedness than all other groups, the adolescents with bulimia perceived significantly lower autonomy and emotional connectedness within the family than their fathers, and the restrictive anorexia nervosa patients perceived higher connectedness than their fathers. The relevance of these findings for understanding family dynamics are discussed.

1. Karwautz, A., Haidvogl, M., Wagner, G., Nobis, G., Wober-Bingol, C., & Friedrich, M. H. (2002). Subjective family image in adolescents with anorexia nervosa and bulimia nervosa. *Zeitschrift fur Kinder- und Jugendpsychiatrie und Psychotherapie, 30*(4), 251-259. <https://doi.org/10.1024/1422-4917.30.4.251>

**Abstract**

Objectives: The family environments of patients with eating disorders have been studied extensively in recent decades. The «Subjective Family Image Test» is an instrument developed especially to measure differential perceptions by family members. Assessments of subjective family image in families of adolescents by means of this test have been carried out in only a few samples. Methods: We aimed first to investigate subjective perceptions by adolescents of their family relations in a larger clinical sampleof female adolescents (n = 118) suffering from anorexia nervosa of either subtype or from bulimia nervosa and to compare these perceptions with those of healthy controls (n = 96). Second, we investigated intra-familial differences in perception. Results:The main findings were that bulimia nervosa patients perceived lower individual autonomy and lower emotional connectedness than all other groups, the adolescents with bulimia perceived significantly lower autonomy and emotional connectedness within the family than their fathers, and the restrictive anorexia nervosa patients perceived higher connectedness than their fathers. The relevance of these findings for understanding family dynamics are discussed.

1. Kaszás, B., Oláh, B., & Kovács-Tóth, B. (2021). The relationship of muscle dysmorphia to the body image and early maladaptive schemata created by parental behaviours. *Psychiatr Hung, 36*(2), 113-123.

**Abstract**

Introduction: the purpose of our study was to investigate the link between specific traits that determine muscle dysmorphia in bodybuilders and the parental behaviours which play a role in developing these schemata, as well as the eating-disorder-specific traits which differentiate bodybuilders from the normal population. Aim: conceptualising the similarities of personality traits in case of muscle dysmorphia and eating disorders, as well as their aetiology based on the schema theory. Method: to measure muscle dysmorphia was used the Muscle Appearance Satisfaction Scale. We explored its connection with eating disorders applying the Eating Disorder Inventory and thereof with the parental behaviours was used the Young Parental Inventory. Results: a number of eating-disorder-specific traits differentiate persons with muscle dysmorphic traits from the normal population. These are Drive for thinness, Body dissatisfaction, Feeling of inadequacy, Interoceptive awareness and Perfectionism. Regarding the perceived parental behaviour, the father violated the child's need of autonomy, competence and self-identity and the mother violated the need of boundaries and self-control. Conclusions: Based on several personality characteristics, muscle dysmorphia may be related to anorexia nervosa and bulimia nervosa. In its aetiology, parental care can have a role, as the father is disposed to detain the autonomy, and the expression of personal needs, while the mother demonstrates insufficient control functions, and the detention of the autonomy and the need for competency.

1. Katena, S., Imai, A., & Shimazaki, H. (2004). Parent-child relationships of eating disorders prone female university students. *Japanese Journal of Health Psychology, 17*(2), 32-41. <https://doi.org/10.11560/jahp.17.2_32>

**Abstract**

The influence of parental child-rearing attitudes and children's attitudes regarding the way their parents rear them on proneness to eating disorders as measured by the Eating Disorders Inventory (EDI) was investigated. The parental child-rearing attitudes were measured using the Family Relationships Inventory (FRI). The attitudes of children regarding how their parents rear them were measured by Parental Bonding Instrument (PBI). The responses of 89 female university students and their parents indicated that “PBI-overprotection” score of the father and “PBI-care” score of the mother explained the “EDI-proneness to eating disorders” and “EDI-bulimia” scores. It was also suggested that PBI-care and overprotection scores of parents and “FRI-communications between parents” score of the father might predict “EDI-ineffectiveness” and “EDI-perfectionism”. It is concluded that the attitude of female students regarding how their fathers rear them had a significant influence on the “EDI-proneness to eating disorders”.

1. Kiriike, N., Matsunaga, H., Nagata, T., Tobitani, W., & et al. (1995). Eating disorders and marriage. II. Twenty-five patients with postmarital onset of eating disorders. *Seishin Igaku, 37*(11), 1147-1153. Retrieved from <http://ovidsp.ovid.com/ovidweb.cgi?T=JS&PAGE=reference&D=psyc3&NEWS=N&AN=1997-85327-001>.

**Abstract**

Conducted a retrospective study of the relationship between marriage and late-onset eating disorders. Human Ss: 25 female Japanese adults (aged 24.1-51.9 yrs) (anorexia nervosa, bulimia, or an unspecified eating disorder) (married). 21 female Japanese adults (mean age 30.2 yrs) (tardive anorexia nervosa or bulimia) (unmarried). Medical histories, marital histories, and demographic data were analysed for Ss with restrictive anorexia nervosa, binge-eating or purging anorexia nervosa, and purging or non-purging bulimia. The course and clinical features of the disorders were assessed. This article is Part 2 of another article.

1. Klingenspor, B., & Rastetter, D. (2004). Gender Identity Development and Bulimic Eating Behavior in Adolescence. *Zeitschrift fur Sozialpsychologie, 35*(2), 67-82. <https://doi.org/10.1024/0044-3514.35.2.67>

**Abstract**

Bulimic eating disorders are predominantly diagnosed in women. Social-psychological factors, in particular stereotypes that associate gender with specific personality traits, are thought to influence eating behavior and to cause eating disorders. Previous findings suggest that bulimic eating behavior is linked to the suppression of masculine traits. In order to test this hypothesis, discrepancies between the actual and the ideal self were examined as precursors of low self-esteem and bulimic eating behavior in adolescents between the ages of 13 and 20. Data were collected in an anonymous questionnaire study and analyzed with structural equation modeling. The discrepancy between how much they believed to possess masculine traits (actual self) and how much they would like to (ideal self) increased with age in girls in contrast to boys, while the discrepancy with regard to feminine traits decreased in both groups. For both sexes, bulimic eating behavior was related to masculine self-discrepancies indirectly, via self-esteem and dieting.

1. Kopp, W. (1994). Frequency of sexual abuse among women with eating disorders. *PPmP: Psychotherapie Psychosomatik Medizinische Psychologie, 44*(5), 159-162. Retrieved from <http://ovidsp.ovid.com/ovidweb.cgi?T=JS&PAGE=reference&D=psyc3&NEWS=N&AN=1994-88232-001>.

**Abstract**

Studied the incidence of sexual abuse in female inpatients with eating disorders. Ss were 14 obese females with psychogenic hyperphagia, 18 bulimic females, 22 females with anorexia nervosa, and 73 female psychotherapy or psychosomatic medicine inpatients without eating disorders. Ss' medical records were reviewed for data on incidences of sexual abuse and age at the time of abuse. Intergroup differences were analyzed.

1. Korkina, M. V., Tsivilko, M. A., Kareva, M. A., Zhigalova, N. D., & Kislova, E. K. (1990). The clinico-psychological correlations of mental rigidity in anorexia nervosa. *Zhurnal Nevropatologii i Psikhiatrii imeni S.S. Korsakova, 90*(10), 86-89. Retrieved from <http://ovidsp.ovid.com/ovidweb.cgi?T=JS&PAGE=reference&D=psyc3&NEWS=N&AN=1991-75565-001>.

**Abstract**

Conducted an experimental psychological study of mental rigidity in inpatients and outpatients. Human subjects: 40 male and female adolescents and adults (aged 15-30 yrs) (anorexia nervosa within the framework of borderline mental pathology or schizophrenia). 58 male and female children, adolescents, and adults (mothers, fathers, and sisters of the patients). Tests used: The Tomsk Rigidity Questionnaire by Korkina.

1. Kugu, N., Akyuz, G., Dogan, O., Ersan, E., & Izgic, F. (2002). Prevalence of eating disorders in a university student population and the investigation of its relation with self-esteem, family functions, childhood abuse and neglect. *Psikiyatri Psikoloji Psikofarmakoloji Dergisi, 10*(3), 255-266. Retrieved from <http://ovidsp.ovid.com/ovidweb.cgi?T=JS&PAGE=reference&D=psyc4&NEWS=N&AN=2002-11284-005>.

**Abstract**

Examined the prevalence of eating disorders among university students and studied sociodemographic data, history of childhood abuse and neglect, and family function of students with eating disorders compared with controls. Ss were 980 students (aged 18-24 yrs) who were given the authors' development and eating attitude test. Diagnoses were made with interviews using DSM-IV (SCID-I) with students who had points above the cut values in the eating attitude test. The Rosenberg Self Esteem Scale, the Family Functioning Assessment Device (FFAD), and the Childhood Abuse and Neglect Questionnaire Form were given to all Ss. Results show that of the 71 students at or above the cut point on the eating attitude test, 21 were identified as having an eating disorder with SCID-I, 18 females and 3 males. There were 15 cases of bulimia nervosa and 6 cases of binge eating disorder (BED). There was no case of anorexia nervosa. All 3 males were diagnosed with BED. In the case group, menstrual cycle irregularity was more prominent, self-esteem was much lower, and history of childhood sexual and emotional abuse was significantly more prominent. The points of FFAD of family communication, association, and emotional context was significantly lower in the case group.

1. Kuipers, G. S., van der Ark, L. A., & Bekker, M. H. J. (2020). Attachment, mentalization and autonomy in anorexia nervosa and bulimia nervosa. *Tijdschrift voor Psychiatrie, 62*(2), 148-156. Retrieved from <http://ovidsp.ovid.com/ovidweb.cgi?T=JS&PAGE=reference&D=psyc19&NEWS=N&AN=2020-12951-006>.

**Abstract**

Background: Attachment insecurity and difficulty with mentalization are common in patients with eating disorders and might be related to their symptoms and lack of autonomy.  Aim: To investigate the role of attachment and mentalization in the course of anorexia nervosa (AN) and bulimia nervosa (BN). Method: Patients with AN and BN were assessed at the start of treatment, and after 1 year and 1.5 years concerning attachment security and mentalization in relation to eating disorder and co-morbid symptoms and autonomy. The results at the start of treatment were compared to those of controls without an eating disorder.  Results: Attachment insecurity and low level of mentalization were more prevalent in patients than in controls, and associated with borderline personality disorder and/or self-injurious behaviour. Attachment security increased after 1 year of treatment. Recovery from eating disorder after 1 year was related to higher level of mentalization and improvement of autonomy. Low pre-treatment level of mentalization predicted persistence of eating disorder until 1.5 years of follow-up. 
Conclusion: Good mentalization is associated with recovery from eating disorder. More research on the effect of mentalization based treatment for patients with eating disorders, with or without borderline personality disorder, is recommended.

1. Kumnig, M., Hofer, S., Huber, A., Messner, C., Renn, D., Mestel, R., & Rumpold, G. (2013). Patterns of dysfunctional parenting styles and psychological disturbances in offspring. *Zeitschrift fur Psychosomatische Medizin und Psychotherapie, 59*(4), 356-368. <https://doi.org/10.13109/zptm.2013.59.4.356>

**Abstract**

Objectives: Dysfunctional parenting styles represent a risk factor for the development of psychological disturbances. The present study investigated the differential validity of the Germanlanguage Fragebogen zur Erfassung dysfunktionaler Erziehungsstile (FDEB; Measurement of Parental Styles, MOPS) and determined whether different forms of psychological disorders are associated with specific patterns of parenting styles. Methods: 145 inpatients, 108 outpatients and a control group of 633 representative individuals from the general population were investigated by adapting the FDEB. Results: A comparison of dysfunctional parenting styles showed different distress levels within the diagnostic groups: Patients suffering from depression reported high levels of maternal indifference and overprotectiveness together with an abusive rearing behavior on the part of both parents. Patients with anxiety disorders reported having overprotective mothers. Bulimic patients as well as those with personality disorders significantly exhibited stress in almost all areas. However, anorexic patients did not differ significantly from the control group, which appeared to be the least affected of all.  Conclusion: The FDEB showed a satisfactory differential validity. There was evidence that specific patterns of dysfunctional parenting styles were associated with different diagnostic groups.

1. Laporte, L., Marcoux, V., & Guttman, H. A. (2001). A comparison of the characteristics of families of women with restricting anorexia nervosa and a control group. *L'Encephale: Revue de psychiatrie clinique biologique et therapeutique, 27*(2), 109-119. Retrieved from <http://ovidsp.ovid.com/ovidweb.cgi?T=JS&PAGE=reference&D=psyc3&NEWS=N&AN=2001-07116-001>.

**Abstract**

Compared some psychopathological, psychological, and psychodynamic characteristics of families of women with vs without restricting anorexia nervosa. Ss were 28 anorexic young women (mean age 22 yrs), 27 normal young women (mean age 21 yrs), and their parents in Canada. Ss participated in a semi-structured individual interview and were administered the Eating Attitudes Test (D. M. Garner et al, 1982), the SCL-90 (Revised), the Parental Bonding Instrument, and/or the Self-Report Family Inventory (R. Beavers et al, 1985). Chi-square analysis, ANOVAs, and Pearson's correlation analysis were performed. The results show that (1) parents of anorexic Ss did not have more eating problems but had more problems with alcohol consumption than parents of normal Ss, (2) both sets of parents perceived family functioning similarly, and (3) anorexic Ss reported experiencing more maternal control, intrusiveness, and overprotection than normal Ss reported.

1. Leal, F. J. V., Macias, J. A. G., Garcia-Herraiz, M. A., Lopez Vinuesa, B., Bautista, M. M., & Peralvarez, M. B. (2005). History of sexual abuse in patients with bulimia nervosa: Its influence on clinical status. *Actas Espanolas de Psiquiatria, 33*(3), 135-140. Retrieved from <http://ovidsp.ovid.com/ovidweb.cgi?T=JS&PAGE=reference&D=psyc5&NEWS=N&AN=2005-08963-001>.

**Abstract**

Introduction: Studies analysing the relationship between sexual abuse and bulimia nervosa (BN) have reported discrepant results. This study aimed to assess the role of a history of sexual abuse in the clinical status of a group of patients diagnosed of BN using DSM-TV diagnostic criteria.  Methods: Seventy patients with BN were assessed using specific clinical tools: Eating Attitudes Test-40 items (EAT-40), Bulimia Investigation Test Edinburgh (BITE), Symptom Checklist (SCL-90), Structured Clinical Interview for DSM-IV Axis II Personality Disorders (SCID-II), Sixteen Personality Factors Test (16-PF) and a clinical interview for the assessment of past and current substance abuse. The data from the 15 patients with a history of sexual abuse (21.4% of the sample) were compared with those from the 55 patients without such a history.  Results: Both groups were very similar regarding symptom severity. Only the tendency to somatization and higher scores in the factor E of the 16-PF (dominance) were associated with antecedents of sexual abuse in the sample. Conclusions: The results support the idea that sexual abuse may be related to higher non-specific vulnerability to psychopathology, but do not increase symptom severity in BN patients.

1. Leger, J. M., Blanchinet, J., & Vallat, J. N. (1969). In the light of two cases of mental anorexia in boys, can an important role be attributed to the father's personality for the onset of this illness? *Annales Medico-Psychologiques, 2*(1), 101-108. Retrieved from <http://ovidsp.ovid.com/ovidweb.cgi?T=JS&PAGE=reference&D=psyc2&NEWS=N&AN=1970-15010-001>.

**Abstract**

Notes renewed interest in masculine mental anorexia and presents 2 cases that illustrate a recently advanced hypothesis. This diagnostic was confirmed by various examinations in 2 boys suffering from alimentary disorders with emaciation. Both developments were favorable, accompanied by a bulimia in the 14-yr-old and almost normal alimentary equilibrium in the 16-yr-old. The personality characteristics of the fathers and their roles during the treatment (comparable to that of mothers in young female anorexics) are stressed in a pathogenic interpretation that relies heavily on the correlation between a theft and the onset of anorexia in the younger boy.

1. Leonidas, C., & dos Santos, M. A. (2022). Unpaid balance of the transgenerational legacy: The separation-individuation process involved in the early genesis of eating disorders. *Agora: Estudos em Teoria Psicanalitica, 25*(2), 10-19. <https://doi.org/10.1590/1809-44142022-02-02>

**Abstract**

This study investigated the experience of mothering in women whose daughters were diagnosed with anorexia nervosa or bulimia nervosa, seeking to identify aspects of the early mother-daughter bond that are related to psychopathological symptoms. We started from the assumption that severe symptoms in the eating sphere express specific elements of the early relational dynamics with the mother figure. Considering the theory of transgenerational psychic transmission, we conjecture that the constitution of the symptom has as an articulating element a fusional bond and involves an unsuccessful attempt to place a trace of separation and protection of the daughter against maternal intrusions and excesses. Relationships with transgenerational contents are established.

1. Leonidas, C. & dos Santos, M. A. (2015). Affective and family relationships of women with anorexia and bulimia. *Psicologia: Teoria e Pesquisa, 31*(2) 181-191. <https://doi.org/10.1590/0102-37722015021711181191>

**Abstract**

This study aimed to investigate the social support network of women with eating disorders, emphasizing their affective family relations. The sample consisted of 12 participants assisted at a university hospital. The instruments used were a semi-structured interview, Network's Map and Genogram. The results indicated that participant's social networks are restricted, with prominence of family members in their composition. Family relations, however oscillate between affective turbulence and detachment. The affective relationships held with parents, husbands and boyfriends are marked by disagreements and dissatisfactions. The low density of the network of friends and the impoverishment of social life result in isolation and difficulties in initiating and/or maintaining lasting affective relationships.

1. Leopold, S., Sosnowsky-Waschek, N., Gie, K., Junne, F., Zipfel, S., & Resmark, G. (2022). Information about eating disorders: The perspective of relatives. *Zeitschrift fur Psychosomatische Medizin und Psychotherapie, 68*(3), 297-313. <https://doi.org/10.13109/zptm.2022.68.3.297>

**Abstract**

Objectives: This qualitative study aimed to assess the need for information in eating disorders from the perspective of relatives. Further, it examined the question how publicly available information about eating disorders may contribute to psychoeducation.  Methods: Thirteen relatives of patients with anorexia nervosa and bulimia nervosa were interviewed based on a semi-structured interview guide. The interview transcripts were interpreted by using a structuring qualitative content analysis. Results: Relatives showed a high need for information throughout the entire course of illness. More information about the disorder itself and its potential treatment were requested. The needs were addressed to public services and to family doctors as well as paediatricians. Conclusions: Relatives as well as family doctors and paediatricians play an important role in the detection and support of the treatment of eating disorders. In view of the high rate of cases diagnosed late or not at all, psychoeducational offers require further expansion.

1. Loewens, I., Reich, G., & Meyer, T. (2022). Do siblings have a different influence on self-assessed family functioning in patients with anorexia and bulimia nervosa? *Praxis der Kinderpsychologie und Kinderpsychiatrie, 71*(6), 528-542. <https://doi.org/10.13109/prkk.2022.71.6.528>

**Abstract**

The impact of family factors, particularly the sibling status, on the development and course of eating disorders has been rarely investigated. Therefore, the aim of the present study was to assess a putative association between sibling status and self-rated family dysfunctionality in a large sample of study participants with bulimia nervosa and anorexia nervosa. A total cohort of n = 568 outpatients aged twelve years and older, of whom half had the diagnosis of anorexia (n = 288, 50.7 %), was assessed for self-rated family dysfunctionality using the well-validated German General Family Questionnaire (FB-A), while the symptom level (Global Severity Index) was determined using the SCL-90-Rsymptomchecklist. Patients with anorexia were more frequently only children compared to the bulimia group who had generally more siblings (22.6%vs. 14.4%, p = 0.012). In patients with bulimia nervosa, bivariate analyses revealed a significant positive association between the presence of siblings and the degree of family dysfunctionality (only child: 33.6 ± 17.6, sibling child: 39.3 ± 15.5, p = 0.043). Linear regression models adjusted for age, body-mass index, parents living in separation, and the Global Severity Index (GSI) confirmed a significant association between higher family dysfunctionality as a dependent variable and sibling status (beta = 0.163, 95%confidence interval [CI] = 1.431; 12.774, p = 0.014).No such relationship was found in the anorexia group (p = 0.418).The differential associations between sibling status and self-rated family dysfunctionality found for the two eating-disorder entities may indicate differences in the pathogenesis of anorexia and bulimia nervosa.

1. Lopez, C. A., Munoz, A., & Ballesteros, B. P. (2005). Changing socio-verbal context in women at risk of developing alimentary problems: A relational frame approach. *Revista Latinoamericana de Psicologia, 37*(2), 359-378. Retrieved from <http://ovidsp.ovid.com/ovidweb.cgi?T=JS&PAGE=reference&D=psyc5&NEWS=N&AN=2005-11245-008>.

**Abstract**

The general aim of this study was to analyze the effects of changing social-verbal context in three female adolescents at risk of developing anorexia nervosa and/or bulimia. A single-case design with 3 participants was applied, with measurements pre, during and follow-up (immediately after, after 15 days, and after a month). Self-records, behavioral interview and the Questionnaire of Food-Intake Behavior (ECA) were used as instruments; these results aided to define the case formulation, and the intervention objectives. Case analysis and intervention were designed based on relational frame theory (RFT). The intervention consisted of five sessions, two of them in group considering the communalities related to the influence of mass media and the need for nutritional education. Analogies, metaphors and stories were used to facilitate objective attainment. Results showed that the effect of changing the social-verbal context generated concrete actions leading to prevent food-intake problems, through changes in the actual rules functions and in stimulus relations, especially in two participants.

1. López-Coutiño, B., & Nuño-Gutiérrez, B. L. (2009). Between clumping and stiffness; family structure of two generations of young people with anorexia or bulimia. *Rev Med Inst Mex Seguro Soc, 47*, 47-54.

**Abstract**

Objective: to characterize the structure of two generations with the narrative story of women with anorexia or bulimia. Methods: descriptive, qualitative-type retrospective study. Information was gathered from a genogram of two generations, and focused on tape-recorded interviews with eight young women being treated for anorexia or bulimia. Results: the family structure of both generations was characterized by rigid pattern expressed in a controlled and inflexibility behavior to assume changes displayed in overprotective manners. Contemporary family patterns showed closer bonds between fathers and daughters, and lesser bonds and more rigidity between mothers and daughters. The grandparents' generation did not use health services for attention of eating disorders. Conclusions: bonding and rigidity were the main characteristics of the family structure in both generations. It suggests that these structural patterns are transfered from one generation to the next.

1. Lucka, I., & Cebella, A. (2004). Characteristics of the forming personality in children suffering from anorexia nervosa. *Psychiatria Polska, 38*(6), 1011-1018. Retrieved from <http://ovidsp.ovid.com/ovidweb.cgi?T=JS&PAGE=reference&D=psyc5&NEWS=N&AN=2005-01347-002>.

**Abstract**

Aim: The evaluation of forming personality in 30 children (27 girls and 3 boys) suffering from anorexia nervosa (average age - 13.5). Method: Anamnesis from patients and their parents, clinical observation, psychiatric investigation, psychological investigation with use of following methods: The Thematic Apperception Test, Colourful Pyramids Test, The House-The Tree-The Man Test. Results: After the conducted investigation and observation, the characteristics of the forming incorrect personality were observed in 80% of examined children. According to DSM IV classification, in 50% - from the cluster C, and in 30% cases - the cluster B. Acordnig to ICD-10 classification: 26.6% attributes of dependent personality, 16.6% - histrionic personality, 13.3% avoiding personality, 10% - anankastic personality, 6.7% borderline personality and 6.7% dissocial personality. Conclusions: There is predominating opinion in literature, connecting restrictive type of psychical anorexia, with disorders of personality of anxiety type - the cluster C, and the figure bulimic - with personality the cluster B. Those results were confirmed with statistical significance in the conducted examinations.

1. Magalhaes Bosi, M. L., Luiz, R. R., Uchimura, K. Y., & de Oliveira, F. P. (2008). Eating disorders and body image among physical education students. *Jornal Brasileiro de Psiquiatria, 57*(1), 28-33. <https://doi.org/10.1590/S0047-20852008000100006>

**Abstract**

Objective: To characterize eating practices and possible risk factors associated with eating disorders among physical education students in a public university of the Municipality of Rio de Janeiro, Brazil. Methods: Sectional study, electing as target population a risk group for the emergence of eating disorders. The questionnaires Bulimic Investigatory Test Edinburgh (BITE), Eating Attitudes Test (EAT-26), Body Shape Questionnaire (BSQ) and a variety that considers related issues were applied. Results: A positive result was detected in 6.9% of cases (IC95%: 3.6-11.7%) on EAT-26. On BITE, for elevated symptoms and severe cases, a prevalence of 5% was found (IC95%: 2.4-9.5%) and 2.5% (IC95%: 0.7-6.3%), respectively. It was evidenced that 26.29% of students presented abnormal eating behavior. Conclusion: The results of this study demonstrate the need to pay attention to risk eating behaviors in this group, being justified to take special care with these future health care educators.

1. Manara, F., Caruso, R., Caprioli, C., & Consolati, M. (1996). Symptoms and personality outline in eating disorders: Pathogenetic role of sexual abuse. *Medicina Psicosomatica, 41*(3), 157-170. Retrieved from <http://ovidsp.ovid.com/ovidweb.cgi?T=JS&PAGE=reference&D=psyc3&NEWS=N&AN=1997-04643-002>.

**Abstract**

Studied the prevalence of sexual abuse among female patients with eating disorders. Diagnoses, symptom severity, and personality profiles also were assessed. Human Ss: 75 female Italian adolescents and adults (mean age 26.11 yrs) (eating disorders). Tests used: The Eating Attitudes Test (D. M. Garner and P. E. Garfinkel, 1979), BITE-a self-rating scale for bulimia (M. Henderson and C. P. Freedman, 1987), the SCL-90 and the MMPI-R.

1. Mangweth, B., Hudson, J. I., & Pope, H. G. (1995). The family background of women with bulimia: A cross-cultural comparison of Austrian and American students. *Zeitschrift fur Klinische Psychologie, Psychopathologie und Psychotherapie, 43*(1), 57-74. Retrieved from <http://ovidsp.ovid.com/ovidweb.cgi?T=JS&PAGE=reference&D=psyc3&NEWS=N&AN=1995-86912-001>.

**Abstract**

Studied family factors in Austrian and American women with bulimia. Human Ss: 33 female Austrian adults (mean age 22 yrs) (university students) (bulimia). 33 female American adults (mean age 20 yrs) university students) (bulimia). Ss completed interviews and questionnaires assessing their own symptoms of bulimia and their families' sociodemographic characteristics, interactional climates, child-rearing practices, eating behaviors, attitudes toward sexuality, physical and sexual abuse, and histories of psychiatric disorders. Results from the 2 samples were compared.

1. Marco, J. H., Perpina, C., & Botella, C. (2014). The treatment of the body image disturbances in eating disorders and clinically significant change. *Anales de Psicologia, 30*(2), 422-430. <https://doi.org/10.6018/analesps.30.2.151291>

**Abstract**

Body image disturbance is a significant maintenance and prognosis factor in eating disorders. Hence, existing eating disorder treatments can benefit from direct intervention m patients' body image. This paper includes a controlled study comparing cognitive behavioural treatment for eating disorders with and without a component for body image treatment using virtual reality techniques. The objective of this work was to check if both types of treatment produced a change clinically significant at the end of the treatment and the follow-up to the year, in body image, eating disorders and general psychopathology. Thirty-four participants diagnosed with eating disorders were treated and compared with the general population with low vulnerability to eating disorders. Results showed that only in the patients who received the body image treatment obtained a change clinically significant. The implications and limitations of these results are discussed below.

1. Marocco Muttini, C. (1993). The father's role and dysorexia. *Minerva Psichiatr, 34*(3), 159-62.

**Abstract**

Of all eating disorders it is widely acknowledged that dysrexia has increased; this syndrome appears most frequently to affect the more affluent socio-economic classes. This paper focuses on a single case from which more general conclusions are then drawn. After an earlier episode of bulimia, a girl suffering from anorexia was found to live in a family context in which the father provided a weak psychological reference point. He was often away on work during the girl's childhood and only takes part in the family life as the provider of material wellbeing without playing a direct affective or pedagogical role. A situation of this type represents a way of fulfilling family roles in today's society where ensuring the family's comfortable lifestyle acts as a substitute for the affective role which the parent is not able to sustain due to unresolved neurotic nuclei. Society can now augment certain forms of defence in view of efficiency and productivity which detract from a genuine personal evolution. This leads to the development of neuroses in the younger generation which the mental health services must identify and correct using new psychopedagogic proposals.

1. Martinez, M. A. G., Bernabe, J. R. Y., Ruiz, A. S., & Rodriguez, M. C. (2011). Assessment of emotional reactivity to food images in bulimia nervosa. *Psicothema, 23*(4), 580-586. Retrieved from <http://ovidsp.ovid.com/ovidweb.cgi?T=JS&PAGE=reference&D=psyc10&NEWS=N&AN=2011-24431-008>.

**Abstract**

The aim of the study was to assess the influence on reactivity to food images of the following variables: craving-trait, positive or negative mood state, and food restriction. Emotional modulation of the defense startle reflex (RMS) was assessed in 26 women at risk of suffering from bulimia nervosa; they were assigned one of to two groups: high craving-trait and low craving-trait. Before the test, positive or negative mood and restriction vs. non-restriction states were induced in each of the groups. Skin conductance response (SCR) and electromyogram activity from the orbiculari oculi region were recorded after the auditory stimuli; questionnaires such as Food Craving Trait Questionnaire (FCQ-T) and the Self-assessment Manikin (SAM) were used. Results showed that negative affect produced a negative valence of food images, more arousal, and more loss of control, as well as higher SCRs. Subjects with low FCQ-T levels reduced their RMS to food images as a consequence of experiencing positive emotions; when emotions were negative, their RMS increased.

1. Martins, C. R., & Petroski, E. L. (2015). Dissatisfaction with body image among female adolescents in a small Brazilian town: Prevalence and correlates. *Motricidade, 11*(2), 94-106. Retrieved from <http://ovidsp.ovid.com/ovidweb.cgi?T=JS&PAGE=reference&D=psyc14&NEWS=N&AN=2015-39584-009>.

**Abstract**

This study was designed to identify the prevalence of dissatisfaction with body image and factors associated with it among adolescents from a small town in Brazil. A total of 144 female students (nine to 20 years old) from the town of Sao Bonifacio, SC, took part. Data were collected on the variables age, location of residence, monthly family income and time spent watching TV on weekdays and at the weekend. The BSQ and EAT-26 were used to assess body image and risk of eating disorders. Body fat percentages were calculated from skin fold measurements. Sexual maturity stages were classified on the basis of pubic hair and breast development and menarche. Logistic regression analysis was used to treat data (p < 0.05). The prevalence of dissatisfaction with body image was 26.4%. Adolescent females at risk of eating disorders were 7.15 times more likely to be dissatisfied (RC = 7.15; 95%CI = 2.67-19.17; p = 0.0001). These results highlight the importance of planning and implementing strategies within schools designed to promote greater body satisfaction among female adolescents, in order to prevent eating disorders in this population.

1. Masjuan, M. G., Aranda, F. F., & Raich, R. M. (2003). Bulimia nervosa and personality disorders: A review of the literature. *International Journal of Clinical and Health Psychology, 3*(2), 335-349. Retrieved from <http://ovidsp.ovid.com/ovidweb.cgi?T=JS&PAGE=reference&D=psyc4&NEWS=N&AN=2003-00261-008>.

**Abstract**

Discusses the relationship between bulimia nervosa and other psychiatric disorders. In bulimia nervosa, as in the case of many other mental health disorders, a high comorbidity with other psychiatric disorders has been described, with special attention paid to personality disorders. Despite many studies devoted to the examination of personality disorders and its association with eating disorders, the importance of this topic is being considered contradictorily in the literature, ranging from 27%-84% of cases with personality disorders. Those of cluster B and especially Borderline Type are the most represented in the literature. In eating disorders literature, personality disorders have frequently been related to several factors, including: (1) diagnostic subtypes; (2) higher impulsiveness and self-injurious behaviors; (3) greater substance and alcohol abuse; (4) suicide attempts; (5) greater frequency of purging behaviors; (6) mood disorder; (7) sexual abuse; and (8) higher comorbidity. Furthermore, personality disorders are found to be a predictor of poorer outcome and were related to higher treatment dropout rates.

1. Mateos-Agut, M., Garcia-Alonso, I., de la Gandara-Martin, J. J., Vegas-Miguel, M. I., Sebastian-Vega, C., Sanz-Cid, B., & Martin-Martinez, E. (2014). Family structure and eating behavior disorders. *Actas Espanolas de Psiquiatria, 42*(6), 267-280. Retrieved from <http://ovidsp.ovid.com/ovidweb.cgi?T=JS&PAGE=reference&D=psyc13&NEWS=N&AN=2015-02406-001>.

**Abstract**

Introduction: The modern way of life, characterized by the cult of individualism, discredited authority, and a proliferation of points of view about reality, has modified family structure. This social structure imbues families and the way that its members become ill, in such a way that eating behaviour disorders (EDs) have become a typically postmodern way of becoming ill. Methodology: The aim is to understand the systemic structure and vulnerability of families by comparing 108 families with members who have ED to 108 families without pathology. A questionnaire administered by an interview with trained personnel was used. Results: Families with ED have a different structure from the families in the control group. They have more psychiatric history and poor coping skills. The family hierarchy is not clearly defined and the leadership is diffuse, with strict and unpredictable rules, more intergenerational coalitions, and fewer alliances. The relationship between the parents is distant or confrontational, and their attitudes towards their children are complacent and selfish, with ambivalent and unaffectionate bonds. In the case of mothers, this is manifested by separation anxiety and dyadic dependence. Their expectations concerning their offspring are either very demanding and unrealistic, or indifferent, and there is less control of their behaviour, in addition to poor organization of the family meals. Conclusions: The structural differences between the two groups of families seem to be important for the occurrence and maintenance of EDs, although they may not be the only cause. The results suggest strategies for clinical intervention in EDs.

1. Meilleur, D., Jamoulle, O., Taddeo, D., & Frappier, J. (2014). Behavioral and personal characteristics in children aged 8 to 12 years old (n = 215) with an eating disorder: A retrospective study. *Neuropsychiatrie de l'Enfance et de l'Adolescence, 62*(5), 278-286. Retrieved from <https://www.em-consulte.com/article/912617/resume/caracteristiques-comportementales-et-personnelles->

**Abstract**

Objective: This study aimed to describe psychological, sociodemographic and somatic characteristics of all children aged 8–12 years who received treatments for eating disorders on a pediatric unit of a University Health Centre during a period of 15 years. Methods: We reviewed the medical records of 215 children with a detailed grid. Descriptive statistics (*t*-test, Chi^2^) were performed. Results: The overall sample was comprised largely (92%) of girls (*n*=197); 4% of whom were twins. Out of the 215 children, 52% were hospitalized at least one time and 48% were seen and treated as outpatients. In the sample, 82% expressed concerns towards food/weight, 69.4% were afraid of gaining weight, 57.5% still want to lose weight and 46.6% had body image preoccupations. Most of the children, e.g. 95%, had restrictive eating behaviors and 13.5% had bulimia symptoms. Eating problems during infancy were present in 15.9% of the sample. More than half of the children (55.8%) had at least one comorbid condition and family history of psychiatric problems was observed for 36.3% of the children. Boys and girls obtained very similar results on all variables of the study. Only one significant difference had been observed between them: boys were more socially withdrawn. Some significant differences were found between younger children (8–10 years old) and the older one (10–12 years old) of the sample. Conclusions: These results bring out questioning on the etiology of ED in children and on their outcome. Follow-up studies of children presenting ED are essential to better understand these problems and their evolution in order to adapt our strategies for treating them.

1. Melosi, A. (2000). Bulimia: Psychopathology and psychotherapy in a systemic perspective. *Ecologia della Mente, 23*(1), 27-49. Retrieved from <http://ovidsp.ovid.com/ovidweb.cgi?T=JS&PAGE=reference&D=psyc3&NEWS=N&AN=2000-06000-002>.

**Abstract**

Studied family dynamics, treatment, and treatment outcome in the case of 20 female adolescents and adults (aged 16-42 yrs) with purging or nonpurging bulimia in Italy. Onset of symptoms, motivating factors, personality, family history, and family psychodynamics were assessed. 13 Ss had a history of previous treatment, while 7 Ss were undergoing treatment for the 1st time. Based on specific guidelines, Ss were subdivided into 3 treatment groups: couples therapy, family therapy, or individual therapy. The outcome for each group is examined. The findings suggest that bulimia can be viewed as a signal of a struggle against deep feelings of powerlessness and lack of self-esteem, especially in families characterized by a persistent attempt to define relationships. It is emphasized that therapy at any level should focus on the conflicts and psychological wounds connected with the definition of self and with interpersonal relationships.

1. Mena Matos, P., & Costa, M. E. (1990). Psychological intervention on bulimia. *Cadernos de Consulta Psicologica,* 667-75. Retrieved from <http://ovidsp.ovid.com/ovidweb.cgi?T=JS&PAGE=reference&D=psyc3&NEWS=N&AN=1992-85295-001>.

**Abstract**

Discusses the following topics concerning bulimia: evaluation criteria, clinical characteristics, family interaction patterns, and intervention perspectives (cognitive-behavioral intervention, anxiety reduction, and body-oriented therapy).

1. Mester, H. (1982). Women desiring a change in the size of their breasts: A contribution to the issue of dysmorphophobias. *Zeitschrift fur Psychosomatische Medizin und Psychoanalyse, 28*(1), 69-91. Retrieved from <http://ovidsp.ovid.com/ovidweb.cgi?T=JS&PAGE=reference&D=psyc2&NEWS=N&AN=1982-28311-001>.

**Abstract**

The desire to reduce or augument breast size is interpreted as a neurotic maneuver. Biographical data reveal how patients' choices of partners were influenced by internal principles. The pathological components of these females' relationships with males differed widely from the marital relationships of females with a different basic neurotic structure. Severe psychic disorders were significantly more frequent among females desiring surgery for the enlargement of their breasts. Among females wishing to reduce their breast size, more than half showed symptoms of anorexia or bulimia.

1. Miranda, M. R. (2004). The anorexic object world and the bulimic violence of teenage girls. *Revista Brasileira de Psicanalise, 38*(2), 309-334. Retrieved from <http://ovidsp.ovid.com/ovidweb.cgi?T=JS&PAGE=reference&D=psyc5&NEWS=N&AN=2004-20479-004>.

**Abstract**

The psychoanalytical encounter generates the perception that anorexia and bulimia are manifestations having origin in psychological suffering, oral symptoms which hind archaic anguish, linked to primitive stages of the mind development, especially as early ruptures with an internalized maternal image. A story of passion, mother and daughter bound together, dependent on each other, but at the same time feeling haunted by the dependence which involves their relationships, fused in a corporeal prison, in a desire perversion, in eternal search for filling an empty interior that comes from their violent object world, looking for meaning to some affects printed on their body and on their acting-out.

1. Monari, C. (2001). Body images in projective tests. *Interazioni*, 263-77. Retrieved from <http://ovidsp.ovid.com/ovidweb.cgi?T=JS&PAGE=reference&D=psyc3&NEWS=N&AN=2002-13522-006>.

**Abstract**

Describes a clinical approach to Rorschach testing that integrates Paul-Claude Racamier's anti-Oedipal theory. Brief clinical studies are presented to illustrate the relational experience of adolescent and adult patients with mysterious ailments, bulimia, the inability to separate, physical abuse, and hereditary disorders.

1. Moretti, P., Fontana, F., Eusebi, P., & La Ferla, T. (2010). Males eating disorders: Importance of body image distortion. *Giornale Italiano di Psicopatologia / Italian Journal of Psychopathology, 16*(2), 150-156. Retrieved from <http://ovidsp.ovid.com/ovidweb.cgi?T=JS&PAGE=reference&D=psyc9&NEWS=N&AN=2010-22450-004>.

**Abstract**

Objectives: To evaluate the prevalence of the risk condition for the eating disorders in males; to identify possible psychological factors related to such condition, among which, body image distortion; and to evaluate the relationship between risk conditions for eating disorders and some body image change strategies in teenager males. 
Methods: The selected subjects for this work were hosted at the Convitto Unificato INPDAP of Spoleto (PC, Italy), in May 2006 (n = 54). The sample was investigated using four self-reported questionnaires: a socio-demographic record purposely prepared by the authors, the EDI-2 (Eating Disorders Inventory; Garner et ai, 2000), the BUT (Body Uneasiness Test; Cuzzolaro et al., 2000), and the AHRB (Adolescent Health Risk Behaviours; McCabe and Ricciardelli, 2006). Positive results on the "Drive for Thinness" (IM) subscale and on the "Bulimia" (BU) subscales of the EDI-2 would indicate a risk condition for eating disorders. Accordingly, the sample was divided into four main groups: IM or BU positive subjects, IM positive subjects, BU positive subjects, IM and BU negative subjects. The subjects resulting negative at both of the subscales represented our control group, the other ones were compared to it individually. We used the Anova to detect differences between people at risk for ED and those not at risk. Results: Socio-demographical record outcomes show that the young males (n = 54) are between 11 and 20 years old, with an average age of 16.5 years (SD = 1.87). The 65% of them comes from the South, the 31 % from the Centre and the remaining 2% from the North of Italy. In our sample, two persons scored high on the IM subscale; eight persons scored high on the BU subscale; and one person scored high on both IM and BU scales. Therefore, nine persons can be considered at risk for eating disorders. The at-risk subjects for eating disorders obtained highly significant scores in some scales (Tables l-lll): * the Perfectionism", the "Interceptive Awareness" and the "Impulse Regulation" scales of EDI-2; * the "CSI ", the "Weight phobia " and the "PSDI" scales of the BUT; * the "Body Image Importance", the "Body Change Strategies to Decrease Weight", the "Food Supplements" and the "Body Change Strategies to Increase Muscles" scales of the AHRB. Conclusions: Of the 54 male subjects investigated, 16.6% (n = 9) are at risk for eating disorders. Study results support the importance of body image distortion for eating disorder onset in males. In fact, the following two features are found to be closely related to the risk for eating disorders: low enteroceptive awareness and distorted cognitive-emotional component of body image. From our data analysis, other psychological features seem to be important for the risk of eating disorders: perfectionism and impulsiveness. Our results confirm also the relationship between risk for eating disorders and some dramatic body change strategies that were previously assessed through the AHRB.

1. Munno, D., Kornmuller, A. M., Barbantini, M., & Zullo, G. (2001). Unwanted sexual experiences and eating disorders: Psychotherapeutic implications. *Minerva Psichiatrica, 42*(4), 245-256. Retrieved from <http://ovidsp.ovid.com/ovidweb.cgi?T=JS&PAGE=reference&D=psyc3&NEWS=N&AN=2002-10667-002>.

**Abstract**

Studied the prevalence of unwanted sexual experiences in 20 female adults (aged 18-35 yrs) with anorexia nervosa, 20 female adults (aged 18-35 yrs) with bulimia, and 20 female adults (aged 18-35 yrs) without eating or other psychiatric disorders (controls). Data on sociodemographic variables, clinical and psychological symptoms, eating behavior, and sexual experiences were obtained by questionnaire. The %DSM-IV% and the Sexual Event Questionnaire (P. Calam and P. Slade, 1989) were used. The results indicate that anorexia and bulimia Ss have been exposed to more unwanted sexual experiences involving physical contact and physical force than control Ss and that anorexia Ss have been exposed to a higher incidence of physical abuse than bulimia and control Ss. The results also show that anorexia and bulimia Ss who have experienced unwanted sexual advances report friends as their abusers while control Ss who report unwanted sexual advances report strangers as their abusers. Implications for using emotive-cognitive methods during therapy for eating disorders are discussed.

1. Munsch, S., & Herpertz, S. (2011). Eating disorders associated with obesity and diabetes. *Der Nervenarzt, 82*(9), 1125-1132. <https://doi.org/10.1007/s00115-010-3227-x>

**Abstract**

Binge eating disorder is one of the most frequent comorbid mental disorders associated with overweight and obesity. Binge eating disorder patients often suffer from other mental disorders and longitudinal studies indicate a continuous weight gain during the long-term course. As in other eating disorders gender is a risk factor, but the proportion of male binge eating disorder patients is surprisingly high. In young women with type 1 diabetes the prevalence of subclinical types of bulimia nervosa is increased. In addition, insulin purging as a characteristic compensatory behavior in young diabetic women poses a considerable problem. In patients with type 1 diabetes, disturbed eating and eating disorders are characterized by insufficient metabolic control and early development of late diabetic sequelae. Patients with type 2 diabetes are often overweight or obese. Binge eating disorder does not occur more frequently in patients with type 2 diabetes compared to healthy persons. However, the comorbidity of binge eating disorder and diabetes type 2 is associated with weight gain and insulin resistance. Especially in young diabetic patients a screening procedure for disturbed eating or eating disorders seems to be necessary. Comorbid patients should be offered psychotherapy.

1. Muttini, C. M. (1993). Psychogenic eating disorders and the family set-up. *Minerva Psichiatrica, 34*(4), 225-229. Retrieved from <http://ovidsp.ovid.com/ovidweb.cgi?T=JS&PAGE=reference&D=psyc3&NEWS=N&AN=1994-87012-001>.

**Abstract**

Discusses the relationship between eating disorders and the intrafamily relationships of the victims of these disorders. The significance attributed by different researchers to the number of siblings and birth order of anorexia and bulimia patients is described. The types of relationships with parents and siblings typical of victims of eating disorders are considered.

1. Nagata, T., Kiriike, N., Matsunaga, H., Iketani, T., Kawarada, Y., & Yamagami, S. (1999). A history of sexual or physical abuse in patients with eating disorders. *Seishin Igaku, 41*(2), 165-172. Retrieved from <http://ovidsp.ovid.com/ovidweb.cgi?T=JS&PAGE=reference&D=psyc3&NEWS=N&AN=1999-13376-005>.

**Abstract**

Studied the relationship between sexual or physical abuse and eating disorders. Human Ss: 25 Japanese adults with (anorexia nervosa restricting type) (mean age 22.7+/-7.0 yrs). 31 Japanese adults with (anorexia nervosa binge eating-purging type) (mean age 25.2+/-4.6 yrs). 41 Japanese adults (bulimia nervosa) (mean age 22.2+/-4.0 yrs). 33 normal Japanese adults (mean age 20.4+/-0.9 yrs) (college students) (controls). Ss were administered the Physical and Sexual Abuse Questionnaire (PSA). Results obtained by using the Bonferroni method and SPSS were compared with the North American results. Tests used: The PSA, Diagnostic and Statistical Manual of Mental Disorders-III-Revised (DSM-III-R), and Diagnostic and Statistical Manual of Mental Disorders-IV (DSM-IV).

1. Narvaz, M., & Oliveira, L. L. (2009). Sexual abuse and eating disorders: A review. *Revista Interamericana de Psicologia, 43*(1), 22-29. Retrieved from <http://ovidsp.ovid.com/ovidweb.cgi?T=JS&PAGE=reference&D=psyc8&NEWS=N&AN=2009-08214-003>.

**Abstract**

The relationship between sexual abuse and eating disorders has been widely researched. Sexual abuse as a risk factor for the development of eating disorders, especially bulimia nervosa is, however, heterogeneous. Studies investigating a possible relationship between sexual abuse and eating disorders have reported highly discrepant results. The aim of this paper is to present a theoretical review about this relationship. Despite controversial results, this paper intends to highlight the need for professionals of several areas, especially psychotherapists, to pay attention to this possible relationship in order to reinforce their interventions.

1. Nitkowski, D., Wunsch-Leiteritz, W., Braks, K., Hristova, S., & Petermann, F. (2019). Indirect and direct nonsuicidal self-injury in girls and young women with eating disorders: Do borderline-specific cognitions mediate the relationship? *Kindheit und Entwicklung: Zeitschrift fur Klinische Kinderpsychologie, 28*(4), 220-229. <https://doi.org/10.1026/0942-5403/a000293>

**Abstract**

Nonsuicidal self-injury (NSSI) is common in eating disorders. Nevertheless, findings on the relationship between restrictive-ascetic or bulimic eating behavior and NSSI are not consistent. The aim of our study was to clarify whether borderline-specific cognitions mediate the relationship between restrictive-ascetic or bulimic eating behavior and NSSI. A sample of 74 girls and young women aged 14 – 21 years (*M* = 17.49, *SD* = 1.85) who had anorexia (63.5 %) or bulimia (36.5 %) completed questionnaires on NSSI, borderline-specific cognitions, depressive symptoms, and ascetic and bulimic eating behavior. Results showed that borderline-specific cognitions mediate the relationship between ascetic behavior on one hand and the number of NSSI methods and NSSI for psychological regulation on the other. In the case of bulimic behavior, no such mediation was found. Ascetic behavior in girls and young women with an eating disorder was linked to the number of NSSI methods and to NSSI for psychological regulation via borderline cognitions. An eating behavior characterized primarily by binge eating was not related to NSSI methods or NSSI for psychological regulation. Depressive symptoms were associated with NSSI, but there was no such relationship when borderline cognitions were controlled for. This underlines that NSSI is more closely associated with emotional unstable thought patterns than with depression. In summary, ascetic but not bulimic behavior in girls and young women with anorexia or bulimia is linked via borderline cognitions to a high number of NSSI methods and a more frequent use of NSSI to regulate aversive mental states. Beyond emotion dysregulation and impulsivity, bulimia does not seem to account for NSSI. Further replication of these results is needed. A limitation of this study is the cross-sectional design. Accordingly, it should be clarified whether the conclusions of this study can withstand empirical testing with longitudinal data. In the future, types of dysfunctional eating behavior should be examined separately for a relationship with NSSI.

1. Nomoto, F. (1997). Mental Distance Test: Comparisons among 45 patients with eating disorder, 45 patients with other nonpsychotic disorders and 286 normal controls. *Seishin Igaku, 39*(4), 403-413. Retrieved from <http://ovidsp.ovid.com/ovidweb.cgi?T=JS&PAGE=reference&D=psyc3&NEWS=N&AN=1997-06364-007>.

**Abstract**

Compared nonpsychotic patients' and normal Ss' mental distance to their parents. Human Ss: 45 female Japanese school-age children, adolescents, and adults (aged 11-28 yrs) (anorexia and/or bulimia). 45 Japanese school-age children, adolescents, and adults (aged 11-27 yrs) (generalized anxiety, depression, obsessiveness, school phobia, and hysteria). 302 normal female Japanese school-age children, adolescents, and adults (aged 12-28 yrs) (junior high and high school students, junior college students, and employees) (control group). Ss were asked to indicate on a metric scale how close or distant they felt to their mother and their father. Test used: The Mental Distance Test.

1. Novelle, J. M., & Alvarenga, M. S. (2016). Bariatric surgery and eating disorders: Integrative review. *Jornal Brasileiro de Psiquiatria, 65*(3), 262-284. <https://doi.org/10.1590/0047-2085000000133>

**Abstract**

Objective: To conduct a revision about disordered eating and eating disorders related to bariatric surgery. Methods: Integrative literature’s review on databases PubMed, Lilacs, Bireme, portal SciELO using indexed keywords; inclusion criteria were provided data about the presence or frequency of eating disorders or disordered eating behaviors previously and/or after surgery. Results: One hundred and fifty studies were selected (14 in Brazil and 136 other countries): 80.6% were evaluation of patient’s pre- or post-surgery; 12% were case studies and 7.3% were revision studies. Diverse scales and questionnaires were used for evaluation, mostly the Questionnaire on Eating and Weight Patterns, the Binge Eating Scale and the Eating Disorders Examination Questionnaire. Binge eating was the most common behavior evaluated, with frequencies/prevalence from 2% to 94%; for Binge Eating Disorder frequencies/prevalence’s range from 3% to 61%. Studies also describe anorexia and bulimia nervosa, night eating syndrome and grazing. Some studies point improvement of symptoms while others call attention for the emergence or aggravation of problems. Conclusion: Despite the variability among methods and results, the presence of disordered eating behaviors is highly frequent among bariatric surgery candidates, and could emerge or get worse after surgery. Health care providers must consider carefully these problems due their impact on surgery results and quality of life.

1. Oberst, U., Balta, M., Sanchez Planell, L., & Rangil, T. (2001). Dissociative disorders in a group of eating disordered patients. *Revista de Psiquiatria de la Facultad de Medicina de Barcelona, 28*(5), 284-290. Retrieved from <http://ovidsp.ovid.com/ovidweb.cgi?T=JS&PAGE=reference&D=psyc3&NEWS=N&AN=2002-12491-001>.

**Abstract**

Studied the prevalence of dissociative disorders and history of prior sexual abuse in 34 female adults with eating disorders and 20 female recent medical school graduates (healthy controls) in Spain. Data on sociodemographic variables and clinical and psychological symptoms were obtained by questionnaire. The Eating Disorder Inventory-2 (D. M. Garner, 1998) and the Dissociation and Impulse Control Questionnaire (J. Vanderlinden et al, 1993) were used. The results indicate that 15 Ss with eating disorders and 1 control have dissociative symptoms, that Ss with eating disorders have higher scores on the identity confusion and absorption scales, that Ss with bulimia nervosa have higher scores on the loss of control and amnesia scales than controls. The results also show that 7 of 34 patients with eating disorders have a history of sexual abuse. The role of dissociative symptoms and sexual abuse in the etiology of eating disorders is discussed.

1. Oliveira, J., Oskinis, S., dos Santos, A. C., & Cordas, T. A. (2020). Is there a relationship between self-compassion and food addiction in women with dysfunctional eating behaviors? *Jornal Brasileiro de Psiquiatria, 69*(4), 211-219. <https://doi.org/10.1590/0047-2085000000286>

**Abstract**

Objective: To verify the relationship between self-compassion and food addiction in women with disordered eating behavior. Methods: Women from a support group completed the binge eating scale, cognitive restraint, self-compassion, modified Yale Food Addiction Scale 2.0 (YFAS 2.0), and Hay's questionnaire for compensatory practices. The prevalence of “food addiction” and symptoms, according to YFAS, was identified. To be included, participants should achieve binge eating scores, and for analysis, they were divided according to the presence of compensatory practices. The group was analyzed using Pearson's correlation tests between variables of interest, and the bulimic and compulsive groups were compared with Student's t-test (p < 0.05; JASP software). Results: 190 women participated in the study. According to YFAS, 95.3% (n = 181) had food addiction, and the scale scores showed a negative correlation with self-compassion and binge eating (p = 0.014 for both). The levels of self-compassion showed a negative correlation with questions #3, #5, #6, #8 and #9 of YFAS (p < 0.05). Conclusions: This study brings data to discuss the need to analyze how self-judgment linked to the suffering of the experience of those who show upset eating behavior, affects the scale filling, bringing identification with the notion of addiction since it is culturally accepted.

1. Oliveira-Cardoso, E. A., Coimbra, A. C., & Santos, M. A. (2018). Quality of life of patients with anorexia and bulimia nervosa. *Psicologia: Teoria e Pesquisa,* 34. <https://doi.org/10.1590/0102.3772e34411>

**Abstract**

This study aimed to evaluate the health-related quality of life (HRQoL) of patients with eating disorders (ED). The sample consisted of 40 predominantly young and adult female patients diagnosed with anorexia in outpatient treatment. A HRQoL instrument (SF-36) and the Eating Attitudes Test (EAT-26) were used. Results showed lowered HRQoL, especially in Mental Components. Impairments were observed in six of the eight domains evaluated regarding HRQoL of patients with higher scores on EAT-26, in five domains in patients with 22 years old or more, in General Health Perceptions (p = 0.02) and Mental Health domain (p = 0.03) in patients with less than five years of treatment and in Physical Functioning domain (p = 0.02) of patients with Bulimia Nervosa.

1. Onnis, L. (2015). Belonging and identity in the light of family myths. Therapeutic implications in adolescents' anorexia and bulimia. *Therapie Familiale: Revue Internationale en Approche Systemique, 36*(1), 13-28. <https://doi.org/10.3917/tf.151.0013>

**Abstract**

The author presents a conception of the dialectics between belonging and identity in the perspective of family mythical dimension. The concept of family myth has been revalued as well in the frame of epistemological renewal of systemic psychotherapy as. more recently, by the new interpretations proposed by neuroscientific discoveries. Myth is founder of family belonging as well as of individual identity, two polarities which are complementary, whilst autonomous. But in some situations, in which myths are crystallized and rigidly homeostatic, family belonging, through invisible loyalties, becomes constrictive for the individual identity development which risks to be blocked. This evidently implies the possible origin of psychic suffering particularly in a very evolutive age as adolescence. The use of an analogical language in therapy, particularly the Sculptures of Family Time (S.T.F.) method allows to explore this mythical dimension and to give suffering new meanings. A clinical case of an anorexic adolescent is presented.

1. Onnis, L., Antenucci, M., Benedetti, P., Bernardini, M., D'Amore, S., Dentale, R. C., …& Tarantino, F. (2000). When time is suspended: The individual and the family in anorexia nervosa. *Ecologia della Mente, 23*(1), 11-25. Retrieved from <http://ovidsp.ovid.com/ovidweb.cgi?T=JS&PAGE=reference&D=psyc3&NEWS=N&AN=2000-06000-001>.

**Abstract**

Discusses anorexia nervosa as a complex syndrome in which sociocultural, familial, and individual components are interconnected. Based on their own work with anorexic patients and their families, the present authors examine the familial aspects of anorexia at an interactional level and a mythical level. The importance of a 3-generational perspective on anorexia is emphasized. The case study of a 17-yr-old female student with bulimic anorexia is discussed with regard to family dynamics and myths and the sculpting of the present and the future.

1. Onnis, L., Barbara, E., Bernardini, M., Fusco, C., & Vietri, A. (2005). Family costellations in DCA: Usefulness of Family Life Space in systemic research. *Ecologia della Mente, 28*(2), 183-203. Retrieved from <http://ovidsp.ovid.com/ovidweb.cgi?T=JS&PAGE=reference&D=psyc5&NEWS=N&AN=2006-03367-002>.

**Abstract**

This study concerns eating disorders, with an emphasis on the Symbolic Drawing of Family Life Space. FLS allows an analysis of relationships and bonds of affection during the early meetings with the family, through a symbolic representation of the family itself. In FLS we ask every member of the family to place himself, other significant persons, groups and organizations, facts and important events, either inside or outside the family life space, and then we also ask to trace bonds of affection. We made a case-control study. The test has been administrated to families with anorexia and bulimia, looking for specific family constellations. Finally, we underline the significant modifications undergone by those constellations in our experimental group due to family therapy.

1. Onnis, L., Barbara, E., Di Giacomo, S., Giambartolomei, A., Mule, A. M., Nicoletti, P. G., & Gentilezza, S. (2009). Relational dynamics in families with adolescent anorexic or bulimic patient: Evidences of a clinical research. *Ecologia della Mente, 32*(2), 169-185. Retrieved from <http://ovidsp.ovid.com/ovidweb.cgi?T=JS&PAGE=reference&D=psyc8&NEWS=N&AN=2010-05693-003>.

**Abstract**

This study is part of a wider clinical research on an Integrated Treatment efficacy in adolescent Anorexia and Bulimia, conducted by the Psychotherapy Unit of the Psychiatric Sciences and Psychological Medicine Department in collaboration with the Neuropsychiatric Department both of "La Sapienza" University of Rome. The relational dimension has been explored with the Wyltwick Family Task method and shows different "Interactional Constellations" in Anorexia and Bulimia related to the Minuchin's Disfunctional Family Interaction Model. This allows to connect the different family disfunctional interaction patterns to the different symptomatic phenomenology of the two anorexic and bulimic diseases, so that it's possible to find out an interpretation key for the different symptomatic expression in the relational background.

1. Onnis, L., Giannuzzi, M., & Romano, C. (2007). A vacuum to fill: Anorexia and bulimia in a transgenerational perspective. *Cahiers Critiques de therapie familiale et de pratiques de reseaux, 38*, 135-157. <https://doi.org/10.3917/ctf.038.0135>

**Abstract**

In this article, the authors, following clinical research data, try to analyze the correlation between the manifestation of anorexic symptom and the existence of problems of non-elaborated losses. Moreover, they try to explain how these nonelaborated losses in the families of origin could influence the developments of family relationships and the constructions of shared values and myths in the following generations. Referring to a transgenerational perspective, the authors explore the meaning of the "myths of unity" so typical and frequent in such families.

1. Ortega, R. O., Chapelo, L. B., & Santoncini, C. U. (2012). Disordered eating behaviors and binge drinking in female high-school students: The role of impulsivity. *Salud Mental, 35*(2), 83-89. Retrieved from <http://ovidsp.ovid.com/ovidweb.cgi?T=JS&PAGE=reference&D=psyc11&NEWS=N&AN=2012-15013-001>.

**Abstract**

Introduction It is widely accepted that psychiatric comorbidity can increase the severity, chronicity, and treatment resistance of psychiatric disorders. In various studies worldwide, it has been estimated that the prevalence of alcohol use disorders in women with disordered eating behaviors (DEB) is situated at between 2.9 and 48.6%. It is worth noting that previous studies have not considered the analysis of the variables that could explain the comorbidity between DEB and alcohol use in adolescents, such as impulsivity, which is the key variable for explaining this comorbidity. On the other hand, most studies have addressed the adult population in psychiatric hospitals or people with eating disorders (ED) or alcohol use disorders. It is considered that those subjects have already developed psychiatric comorbid disorders. Impulsivity could be an unspecific trait that aggravates the psychiatric condition of a determined person and it is therefore more likely for that person to seek specialized care. According to the above, the role of impulsivity in the comorbidity of ED and alcohol use might not be similar to that of the general population, mostly among those who have not yet developed a whole clinical syndrome. Therefore, we consider that it is important to clarify the involvement of impulsivity in the comorbidity between disordered eating behaviors (DEB) and binge drinking (BD) in high school students. It is also crucial to analyze the association between impulsivity and the coexistence of DEB and binge drinking (BD) in female students aged between 15 and 19 years at public high schools in the State of Mexico. Methods Data for this study were drawn from the Project entitled "Prevalence and Factors Associated with Disordered Eating Behaviors in Ado, lescent Women with Different Levels of Urbanization and Migratior Intensity" (CONACyT-SEP-2004-46560). The design for this study is cross-sectional and analytical. A sample of 2357 female students at 11 public high schools in the State of Mexico was randomly selected during the 2006-2007 school year. For data collection for this project, a questionnaire was used that included socio-demographic variables, the Plutchik Impulsivity Scale (PIS), the Brief Questionnaire to Measure Risky Eating Behaviors (BQREB), and the questions on alcohol use included in the Questionnaire of Surveys on Substance Use in Students in Mexico (2003 version). Data were analyzed with the STATA version 10 survey function. Results Impulsivity was associated with the coexistence of DEB and BD (U=224427; p<0.01). The 3.5% of female students with impulsivity presented DEB and BD together vs. 0.6% who did not; 19.6% of female students with impulsivity presented one of the two behaviors vs. 7.8% without this trait. Impulsivity was associated positively and significantly with the coexistence of DEB and BD (t=3.8; p<0.01), regardless of socioeconomic variables, such as the father's educational attainment, the mother's educational attainment, and the number of services in the household. Conclusion The results of this paper indicate a statistically significant association between impulsivity and the coexistence of DEB and BD. This means that there is a greater percentage of coexistence of DEB and BD in female high school students considered to be impulsive in comparison with adolescents without this trait. This occurs regardless of socioeconomic variables, such as the father's educational attainment, the mother's educational attainment, and the number of services in the household. Future research should establish the role of other variables such as depression and examine the association of impulsivity with socioeconomic variables.

1. Ouellet, M., & Monthuy-Blanc, J. (2022). When movement is no longer a synonym of health: A review of treatments for pathological physical exercise in eating disorders. *Annales Medico-Psychologiques, 180*(9), 862-874. <https://doi.org/10.1016/j.amp.2022.01.007>

**Abstract**

Introduction: Pathological physical exercise is a central feature of eating disorder and is related to a poor prognosis. Only few evidence-based recommendations are currently available to guide caregivers in the management of “exerciser” patients. Objective. The purpose of this paper is to present the current state of knowledge regarding proposed treatments targeting specifically the management of pathological physical exercise in patients with any type of eating disorder. Method: The literature search took place in March 2020 and has been updated in February 2021 in databases (PsycINFO, PubMed, MEDLINE, SPORTDiscuss, etc.). English keywords encompassing concepts associated with pathological physical exercise, any type of eating disorder, and treatment were used in various combinations. To be included in the study, the publications identified must be written in English or French and be quantitative or qualitative empirical articles, systematic reviews of the literature or meta-analyses, case studies or syntheses of clinical guidelines. Results: The synthesis of the studies highlights four main classes of pathological physical exercise treatment: adapted physical exercise treatment (*n* = 6), cognitive-behavioral therapy (*n* = 4), thermoregulation (*n* = 1) and pharmacotherapy (*n* = 1). The summary of currently available treatments for pathological exercise indicates benefits of adapted physical exercise and cognitive-behavioral therapy treatments, primarily. These treatments demonstrate efficacy in improving principally the qualitative components of pathological exercise (decrease of compulsion towards exercise, dysfunctional attitude towards exercise, rigidity towards exercise, exercise to regulate emotions and lack of pleasure during exercise and rule-based exercise) in addition to a favorable effect on other symptoms associated with eating disorder (increase of the body mass index, quality of life, motivational change stage and emotional acceptance as well as decrease of the global eating disorder severity, drive for thinness, perfectionism, depression symptoms and psychological distress). Treatments aimed at reducing pathological exercise should include adapted physical exercise combined with cognitive-behavioral therapy principles, principally. Also, pathological physical exercise treatment should (1) promote the benefit of group and pleasure to avoid ascetic and compulsive behaviors as well as provide a variety of movement, (2) include relaxation/rest intervals to reconnect with bodily sensations, (3) include psychoeducational interventions to learn to identify pathological physical exercise and (4) address emotions and cognitions related to exercise to change the function of behavior. Pharmacological treatments should be used only in specific cases and with great caution. Conclusion: While this review points to a predominantly uni- or multi-disciplinary approach to the management of pathological physical exercise, the multidimensionality and complexity of this phenomenon dictates that it be managed holistically through a transdisciplinary team. In this regard, transdisciplinary programs that consider physical exercise as a complex phenomenon transcending sectors of activity must be seriously considered.

1. Pardos-Gascón, E. M., Calmaestra, N. G., & Vacas, M. V. R. (2020). Grid Technique as an Instrument for Evaluation of Eating Disorders: Exploration of Cognitive Structure and Interpersonal Relationships in a Sample of 20 Patients. *Revista De Psicoterapia, 31*(115), 129-146.

**Abstract**

It is intended to offer an evaluation through the Interpersonal Grid 'Technique (TRI) of the factors of structure and cognitive content (self-esteem and self-definition), as well as interpersonal relationships. As well as exploring the relationships between these concepts and traditional clinical factors such as BMI, the age of onset of symptoms, length of treatment, average duration of income hospitalization, number of admissions and time between the onset of symptoms and the first treatment. Likewise, these variables will also be related to the scores obtained in EDI-2 and EAT-26. The study was conducted on a sample of 20 patients with eating disorders from the hospitalization Unit for Eating Disorders of the University HospitalSanJuan de Alicante. The sample had high rigidity, poor flexibility and low cognitive undefined It presented average selfesteem and self-construction with multiple constructs. It is observed that the greater distance with the mother correlates with greater age of onset of symptoms, while the greater distance with the father is related to interpersonal inefficiency, tendency to thinness, body dissatisfaction and lower interpersonal confidence. Also, greater distance between the ideal self and the father correlates with high perfectionism.

1. Pawlowska, B., & Masiak, M. (2007). Analysis of demographic data and family relationships in women with bulimia. *Psychiatria Polska, 41*(3), 365-376. Retrieved from <http://ovidsp.ovid.com/ovidweb.cgi?T=JS&PAGE=reference&D=psyc6&NEWS=N&AN=2007-13583-007>.

**Abstract**

Aim: The aim of this study was an analysis of data collected from the case histories of female patients with a diagnosis of bulimia nervosa, who underwent medical treatment at the Psychiatry Department of the Medical University of Lublin in the years 1993-2003. Methods: 53 female patients with a mean age of 22 years, the age of disease onset was 17.5 years, and mean BMI = 22.3. In this group 58.5% of the patients had secondary level education, primary level--24.5%, vocational--3.8%, and higher-level education--7%. Most patients were city inhabitants (64.2%), and lived together with their parents (85%). Results: Half of the patients were brought up in a complete family, whilst the most frequent cause of being brought up by a single parent was the death of the other parent (26.4%). In this group 54.7% of the patients continued their studies, 13.2% worked and 32% were unemployed. More than half of the patients assessed their relationship both with their fathers (54.7%) and their mothers (58.5%) negatively. 20.8% of the patients were victims of physical violence and 18.8% of sexual abuse. Alcohol was abused by 56.6% of their fathers, 7.5% of their mothers and 18.6% of the patients themselves. A life history of suicide was present in 13.2% of the patients. More mothers (13.2%) compared to the patients' fathers (1.9%), suffered from chronic somatic diseases and mental disturbances; 3.8% of the patients' siblings developed eating disorders. In conclusions we compared data received from patients with anorexia nervosa with data from patients with bulimia. Conclusions: Female patients with a purging type of anorexia are similar to female patients with bulimia in many socio-demographic factors, and at the same time they differ from female patients with a restricting type of anorexia.

1. Pawlowska, B., & Masiak, M. (2007). Comparison of socio-demographic data of female patients with purging and restricting types of anorexia nervosa hospitalised at the Psychiatry Department of the Medical University of Lublin in the years 1993-2003. *Psychiatria Polska, 41*(3), 351-364. Retrieved from <http://ovidsp.ovid.com/ovidweb.cgi?T=JS&PAGE=reference&D=psyc6&NEWS=N&AN=2007-13583-006>.

**Abstract**

Aim: The aim of this study was a comparison of socio-demographic data of female patients with a diagnosis of purging and restricting type of anorexia nervosa. Methods: The method of the study was an analysis of the data obtained from 131 case histories of female patients with a diagnosis of anorexia nervosa (40 patients with restrictive type and 91 with a bulimic type of anorexia), treated at the Adolescent Department and the Neurosis Department of the Psychiatry Department of the Medical University of Lublin in the years 1993-2003. The mean age of the investigated patients with a restrictive type of anorexia was 20.8 years, the age of disease onset was 16.5 years, the mean time of disease duration was 3.7 years, mean BMI = 15.4. The mean age of the patients with a bulimic type of anorexia was 21.8 years, the age of disease onset was 17.9 years, the mean time of disease duration was 4.6 years, mean BMI=15.9. Half of the patients had a secondary level education. 87% were brought up in a full family and the most frequent reason of being brought up by a single parent was the death of another parent. 
Results: Results of our study revealed relevant differences between female patients with a restrictive type of anorexia and a purging type of anorexia in certain family factors. Conclusions: 1. Compared to the patients with the restrictive type of anorexia, much more patients with the bulimic type of anorexia assessed their relationships with their mothers negatively. 2. Much more patients with the bulimic type of anorexia compared to the patients with the restrictive type of anorexia, experienced sexual and physical abuse, in most cases caused by their fathers. 3. Alcohol abuse was present in 53% of the fathers of the patients with the bulimic type of anorexia and 30% of the fathers of patients with the restrictive type of anorexia. 4. Mental diseases occurred more often in mothers of women with a restrictive type of anorexia (10%) than the bulimic type (2.2%). 5. Chronic somatic diseases occurred more often in fathers of the patients with a restrictive type of anorexia (12%) than with the bulimic type (2.2%). 6. Compared to the patients with the restrictive type of anorexia, significantly more patients with the bulimic type of anorexia abused alcohol and had a lifetime history of suicide attempt.

1. Pedinielli, J. L., Bertagne, P., & Mille, C. (1987). "Addictive" pathologies and model of incorporation. *L'Information Psychiatrique, 63*(1), 25-32. Retrieved from <http://ovidsp.ovid.com/ovidweb.cgi?T=JS&PAGE=reference&D=psyc3&NEWS=N&AN=1989-71886-001>.

**Abstract**

Discusses the use of the concept of addiction to establish a relationship among drug addiction, gambling, bulimia, suicidal conduct, toxicophilia, and alcohol abuse. It is maintained that the analysis of these behaviors from the standpoint of incorporation and the primary experience of satisfaction permits addictive behavior to be defined as a rejection of the "lack" category. The relationship between addictive behavior and disregard of paternal problems is considered.

1. Peresmitre, G. G., Garcia, G. P., & Camacho, L. O. (2008). Structural models: Bulimic behavior in interrelation with its risk factors in samples of university students (men and women). *Psicologia y Salud, 18*(1), 45-55. Retrieved from <http://ovidsp.ovid.com/ovidweb.cgi?T=JS&PAGE=reference&D=psyc7&NEWS=N&AN=2012-09930-005>.

**Abstract**

In order to identify a theoretical model that could help to have a better comprehension of the bulimic behavior in interrelation with its main risk factors (thin ideal silhouette, body dissatisfaction, restrained dieting and negative affect), two structural models were built: the dual-pathway model and an alternative one. Both were compared in terms of explained variance, adjustment with the original dual-pathway model, and sex. A probabilistic sample of 196 university students of both sexes participated in the study. To collect data, measures with acceptable psychometric properties were used. The results indicated that the alternative model for women explained the largest amount of variance of bulimic behavior, had the best adjustment with the original dual pathway etiologic model of bulimic pathology, gave the strongest correlations between risk factors predicting bulimic behavior, and showed how the negative affect contributed more to the explained variance. The need of more studies with stronger methods to confirm the results found is discussed.

1. Petrone, P., Prunas, A., Dazzi, S., & Madeddu, F. (2013). ADHD symptoms as risk factors for dysfunctional eating habits in adolescents: Results from a longitudinal study. *Rivista di Psichiatria, 48*(6), 448-455. Retrieved from <http://ovidsp.ovid.com/ovidweb.cgi?T=JS&PAGE=reference&D=psyc12&NEWS=N&AN=2014-07072-005>.

**Abstract**

Aim: The aim of this study is to analyze the relationship between attention deficit hyperactivity disorder (ADHD) symptoms in childhood and early-adolescence and the development of dysfunctional eating habits later in life. The sample under investigation is composed of 217 adolescents (males: 30.9%; mean age: 17.1 +/- 0.88 ys; range: 16-19 ys) voluntarily recruited in the city of Parma (Northern Italy) in the context of a longitudinal research project focused on developmental factors of antisocial behaviour. All subjects were assessed at T1 (mean age: 12 ys) and atT2 (mean age: 14 ys) using a structured clinical interview to collect information on ADHD symptoms on a lifetime basis and, at T3 (mean age: 17 ys), they were administered an interview to assess pathological eating habits. Correlation and regression analyses were carried out between scores of the three symptom domains of ADHD and eating habits as assessed at T3. Results suggest that the association between ADHD symptoms and eating habits show differences according to gender, in that in females ADHD symptoms assessed at T2 are associated with compensatory behaviours, while in males they are predictive of overweight as assessed at T3. ADHD symptoms, although under threshold, may lead to chaotic and unorganized eating habits which might put female at risk for compensatory behaviours and males for overweight.

1. Poerio, V. (1999). Self-other representation in eating disorders: An attachment styles model. *Psicoterapia Cognitiva e Comportamentale, 5*(1), 3-13. Retrieved from <http://ovidsp.ovid.com/ovidweb.cgi?T=JS&PAGE=reference&D=psyc3&NEWS=N&AN=2000-02974-001>.

**Abstract**

Studied self-representation and attachment styles in 32 male and female adults (aged 19-25 yrs) with anorexia and/or bulimia and 20 male and female adults (controls, aged 27-43 yrs) in Italy. Data on sociodemographic variables, clinical and psychological symptoms, self-representation, and attachment style were obtained by semistructured interview and questionnaire. The Eating Disorder Inventory-2 (D. M. Garner, 1991), the Interview for Diagnosis of Eating Disorders (D. A. Williamson et al, 1990), the Millon Clinical Multiaxial Inventory-II (MCMI-II), and the Attachment Style Questionnaire (J. A. Feeney et al, 1994) were used. Discriminant analysis and other statistical tests were used. The results indicate that insecure, dependent, and avoidant attachment styles are associated with eating disorders.

1. Pokrajac-Bulian, A., Stubbs, L., & Ambrosi-Randic, N. (2004). Different aspects of body image and eating habits in adolescence. *Psihologijske Teme,* 1391-104. Retrieved from <http://ovidsp.ovid.com/ovidweb.cgi?T=JS&PAGE=reference&D=psyc5&NEWS=N&AN=2006-02710-006>.

**Abstract**

Body Image is a multidimensional construct which plays a very important role in the development of eating disorders. The purpose of this research was to examine the contribution of global dissatisfaction and affective and cognitive aspects of body image in the development of some symptoms of eating disorders. 270 secondary first and fourth grade schoolgirls participated in this research. The results obtained on girls with differing body mass index has shown that girls with normal weight are the most dissatisfied with their body appearance while seriously underweight girls are significantly more satisfied and less anxious about their appearance. It is established that global body dissatisfaction and body consciousness of social standards which refer to appearance are significant predictors of symptoms of eating disorders (dieting, bulimic behavior, fear of weight gain and food preoccupation). Results of this research are explained in the context of sociocultural theories about the etiology and maintenance of body dissatisfaction.

1. Polanco, J. S., & Gomez-Peresmitre, G. (2009). Structural models, sports of high performance and high-risk eating behaviors. *Psicologia y Salud, 19*(2), 271-280. Retrieved from <http://ovidsp.ovid.com/ovidweb.cgi?T=JS&PAGE=reference&D=psyc8&NEWS=N&AN=2012-13602-012>.

**Abstract**

The objectives of this work were to develop structural models to analyze the relationships, values, and tendencies among risk factors (body dissatisfaction, negative affect as depression, anxiety, hostility or anger and weight concern) and risk eating behaviors (restraint diet and compulsive overeating), and thus defining the role of the sport in such relations. One hundred and fifty adolescent women, elite athletes, and 150 non-athletes, participated in the study. The Questionnaire for Health and Feeding and EFRATA were applied to evaluate risk factors of eating disorders, as well as the Taylor Johnson Temperament Analysis to evaluate negative affect. Results show that body dissatisfaction and a restrained diet were important variables which anteceded and mediated risk eating behaviors and bulimics. Also, it was found that sport decreases the indirect and direct relationships of negative affect in compulsive eating behavior, and that in non-sport women negative affect is a double risk factor to compulsive eating behavior. References to findings that confirm previous results are mentioned and the limitations of this study are discussed.

1. Quadri, M. L., & Garbin, P. (2017). Fathers and daughters. Reflections about the role of the father in family therapy with anorexic and bulimic patients. *Terapia Familiare: Rivista Interdisciplinare di Ricerca e Intervento Relazionale, 114*(1), 37-58. Retrieved from <http://ovidsp.ovid.com/ovidweb.cgi?T=JS&PAGE=reference&D=psyc16&NEWS=N&AN=2017-34331-002>.

**Abstract**

The present article provides the outcomes of the research arisen within the collective considerations of the members of the Mara Selvini Centers with the scope to examine when and how the father is a therapeutic resource. The data come from the Mara Selvini centers' equips, regarding 75 treatments with anorexic and bulimic female patients ended between 01.10.2010 and 31.03.2012. The results confirm the crucial importance of the paternal involvement in the therapy. Some elements connected significantly to a positive conclusion of the treatment emerged: the father's consciousness of the psychological sufferance of the daughter and of the connection between the sufferance and the relationships/ problems system of the family; the presence of a father up rather than a weak father fearful of conflicts; to be a father with an organized attachment system. If positively involved in the therapy, these fathers may become capable of recognizing and valuing the daughter, thus allowing her a self-differentiation and a self-individuation. It is confirmed fundamental the capability of the therapist to modulate the intervention starting from the relational style and the cultural background of the father.

1. Ratte, C., Pomerleau, G., & Lapointe, C. (1989). Study of eating disorders among female college students: Correlation with two psychosocial characteristics. *The Canadian Journal of Psychiatry / La Revue canadienne de psychiatrie, 34*(9), 892-897. Retrieved from <http://ovidsp.ovid.com/ovidweb.cgi?T=JS&PAGE=reference&D=psyc3&NEWS=N&AN=1991-70673-001>.

**Abstract**

Studied the association among academic discipline, parental educational level, and the presence of anorexia nervosa (AN) and other eating disorders in 1,144 female college students. The validity of a screening instrument used to determine the presence of AN was also examined. The Eating Attitude Test-26 (EAT), an abridged version of the EAT-40, by D. M. Garner et al (1979, 1982) was used to screen for AN. Of 245 Ss with a score more than or equal to 20 on the EAT-26 and with weight less than or equal to 80% of ideal weight for 3 yrs previous to the study, 61 were interviewed using semi-structured techniques and clinically examined. The Diagnostic and Statistical Manual of Mental Disorders-III-Revised (DSM-III-R) criteria were used to diagnose AN, bulimia, and atypical eating behaviours. The results were evaluated statistically according to Ss' academic discipline, maternal and paternal educational level, body weight, and presence or absence of eating disorders.

1. Reich, G. (1992). Identity Conflicts in Bulimic Females - Clinical Observations Regarding Interpersonal and Intrapersonal Dynamics. *Forum Der Psychoanalyse, 8*(2), 121-133.

**Abstract**

The entanglement of interpersonal - family dynamic - and psychodynamic factors in the origin of bulimic diseases is described. These disorders are comprehended as impulse neuroses, based on a "two-folded sense of identity", one part of which is shown to the outside world and consciously perceived by the patient and the other part of which is warded off. There are intense open conflicts and impulse action sequences in the patients’ families of origin. Anaclitic needs and the boundaries of privacy are disregarded. Each form of intimacy is experienced by the patients as being overpowered and penetrated. An exceedingly rigid super-ego containing an ideal of strength, as a rule represented by an idealised father, placed into the absolute unrelentingly demands control of oneself. The mother is despised as "weak". On a deeper layer the patient is identified with her. The contradictory identifications cannot be transformed into a unified sense of identity. The distinct defence against affect, especially against feelings of shame, results in a displacement of all wishes of attachment and intimacy as well as all affects connected with them - the second, deeper layer of identity, which is warded off - onto food and eating. The bulimic impulsive action sequence is seen as an attempt to realize these wishes. The transactional and experimental patterns of the patients and their families do reflect changed social patterns of affect control, intimacy and shame.

1. Reich, G. (2005). Family relationships and family therapy of eating disorders. *Praxis der Kinderpsychologie und Kinderpsychiatrie, 54*(4), 318-336. Retrieved from <http://ovidsp.ovid.com/ovidweb.cgi?T=JS&PAGE=reference&D=psyc5&NEWS=N&AN=2005-06413-005>.

**Abstract**

A multitude of empirical studies clearly demonstrates that the origin and course of eating disorders is closely linked to family factors. The influence is exerted in a direct way by conveying attitudes towards food, eating, weight, shape and appearance within the family and in a more indirect way by the family relationships. Families of bulimics differ from those of anorexics by a higher degree of conflict, impulsivity, expressiveness and by lower affective resonance and cohesion. Family therapy has proven to be effective in the treatment of eating disorders. A sketch of a family therapy describes the conflict-oriented approach which includes behavioral elements in oder to stabilize the eating behavior and the weight.

1. Reich, G., Horn, H., Winkelmann, K., Kronmuller, K. T., & Stefini, A. (2014). Psychodynamik focal therapy of bulimia nervosa for female adolescents and young adults. *Praxis der Kinderpsychologie und Kinderpsychiatrie, 63*(1) 2-20. <https://doi.org/10.13109/prkk.2014.63.1.2>

**Abstract**

A manual for a disorder oriented psychodynamic treatment of bulimia nervosa and atypical bulimia nervosa of female adolescents and young adults is presented. This manual is applied in a therapy project, which started in 2007. The work on conflicts and structural dysfunctions is meant to lead to the removal or alleviation of the symptoms and an improvement of eating behavior and body image. The bulimic symptoms are contextualized and focussed according to the conflicts and ego-structural deficits of the patients. Typical patterns of interpersonal relationships, transference, conflict, defence and structural problems as well as therapeutic steps are described. The typical psychosocial situation of female adolescence and young adult age is taken into account. Special emphasis is laid on the limitedness of the therapy to 60 sessions and the active structuring of the final phase of the therapy by the therapist.

1. Reich, G., & von Boetticher, A. (2013). Adolescence and eating disorders-Psychodynamics and therapy of anorexia and bulimia. *PDP Psychodynamische Psychotherapie: Forum der tiefenpsychologisch fundierten Psychotherapie, 12*(4), 228-237. Retrieved from <http://ovidsp.ovid.com/ovidweb.cgi?T=JS&PAGE=reference&D=psyc12&NEWS=N&AN=2015-05248-004>.

**Abstract**

Epidemiology and course of eating disorders are outlined, also differences between anorexia and bulimia nervosa. Psychodynamic aspects focussed on adolescent development and basics of psychodynamic psychotherapy of both eating disorders are presented and exemplified by the presentation of a psychotherapy of an adolescent girl with anorexia nervosa.

1. Reynaert, C., Janne, P., Fontaine, P., Woerlinck, K., & et al. (1991). Towards a circular reunderstanding of Olson's circumplex model: Where the logic of contraries is also a logic of proximities. *Therapie Familiale: Revue Internationale en Approche Systemique, 12*(1), 55-63. Retrieved from <http://ovidsp.ovid.com/ovidweb.cgi?T=JS&PAGE=reference&D=psyc3&NEWS=N&AN=1993-85464-001>.

**Abstract**

Presents a critical review of the circumplex model of marital and family systems of D. H. Olson et al (1973) and its assessment instrument, the Family Adaptability and Cohesion Evaluation Scale. The use of questionnaires in the assessment of family functioning is discussed, with an emphasis on their bipolar, linear conceptions of family dynamics. The present authors' umbrella model of family functioning is introduced. This model is based on the hypothesis that extreme family patterns that appear to be opposite are, in fact, closely connected logically. Models of anorexia and bulimia, rigid and chaotic confusion, rigid and chaotic disengagement, and separation structures are also considered.

1. Rousset, I., Kipman, A., Ades, P., & Gorwood, P. (2004). Personality, temperament and Anorexia Nervosa. *Annales Medico-Psychologiques, 162*(3), 180-188. <https://doi.org/10.1016/j.amp.2003.01.001>

**Abstract**

The multidimensional approach could be the most accurate to analyze eating disorders. It, this perspective anorexia nervosa could be triggered by a non-specific dieting and become a full-blown eating disorder syndrome because of different risk factors. Biological, familial and social influences and premorbid personality disorder could constitute some of these core vulnerability traits. In this review, the relationship between personality features and anorexia nervosa (and its different subtypes) is described. Perfectionism and psychorigidity are major personality traits of patients with anorexia nervosa. The prevalence of personality disorders is higher among anorectic patients than in controls. The anxious-cluster personality disorder is detected in excess in anorexia nervosa, mainly of the restrictive type, including the obsessive-compulsive personality disorder and, to a less extent, the avoidant personality. This cluster was reported to be in excess in patients with anorexia nervosa according to retrospective assessment of premorbide personality disorder, in patient after recovery of comorbid mood or anxiety disorder, and even in weight-restored patients. Patients with anorexia nervosa and a personality disorder have a poorer prognosis and a higher frequency of comorbid psychiatric disorders than those without such comorbidity. The dimensional approach conceptualizes personality disorder as an extreme of normally distributed personality traits. Cloninger distinguished four core dimensions of temperament (novelty seeking, harm avoidance, reward dependence, persistence) and three dimensions of character (self-directedness, cooperativeness, self-transcendance). The use of such quantitative approach of personality profiles allowed to find personality specificities in 8 to 68% of patients with anorexia nervosa who do not have a syndromic personality disorder. High harm avoidance and low novelty seeking characterize patients with anorexia nervosa. Previous descriptions from the literature are in accordance with this description. The association of these two temperament dimensions, specifically distinguishes anorectic patients with the restrictive type. This profile is also stable, as being observed even after recovery. Personality and temperament of patients with anorexia nervosa and comorbid bulimia nervosa, entitled bulimarexia (“boulimarexie”) in french, is less clear-cut, sharing many traits of each personality profile. Detecting a personality disorder and assessing the temperament profile of eating disorder may help to predict outcome, treatment sensitivity, the potential onset of a different eating disorder or also another psychiatric disorder. Assessing personality disorder and temperament profile in patients with anorexia nervosa may thus be a way to further understand the risk factors involved in eating disorder, and could be taken into account when selecting the most appropriate therapeutic strategy.

1. Ruiz-Martinez, A. O., Vazquez-Arevalo, R., Mancilla-Diaz, J. M., Lopez-Aguilar, X., Alvarez-Rayon, G. L., & Tena-Suck, A. (2010). Family functioning in the risk and protection of eating disorders. *Universitas Psychologica, 9(2)*, 433-445. Retrieved from <http://ovidsp.ovid.com/ovidweb.cgi?T=JS&PAGE=reference&D=psyc9&NEWS=N&AN=2010-14439-012>.

**Abstract**

Objective: We analysed the elements of family functioning that explain the risk and protection of ED: Anorexia Nervosa (AN), Bulimia Nervosa (BN) and Eating Disorder Non-Specific (EDNOS). Method: Seventy patients with ED (16 AN, 24 BN and 30 EDNOS) and 30 female students were interviewed and completed three questionnaires: FES, EAT and BULIT. Results: The ED patients (AN, BN and EDNOS) perceived expression was a risk factor and intellectual-cultural activities were protection factors. In the group of ED Specific (AN and BN) organization was a risk factor, and actuation was a protector factor. Conclusion: The results confirmed differences in the family functioning between types of ED Specific and Non-Specific.

1. Sagardoy, R. C., Solorzano, G., Morales, C., Kassem, M. S., Codesal, R., Blanco, A., & Gallego Morales, L. T. (2014). Emotional processing in adult and adolescent ED patients. Emotional recognition and regulation. *Clinica y Salud, 25*(1), 19-37. <https://doi.org/10.1016/S1130-5274%2814%2970024-6>

**Abstract**

Objective: Though difficulties in emotional processing have been associated to eating disorders (EDs), the debate as to whether these difficulties are antecedents or consequence of EDs remains a contentious issue. Most studies have focused on long-term adult patients, but few studies have assessed adolescent patients. This study compared difficulties in emotional recognition and regulation in adult and adolescent patients to determine if they were predisposing factors and/or maintenance factors. Method: A total of 48 patients (27 adults, 21 adolescents) were compared with 41 healthy controls (13 adults, and 28 adolescents) using the Toronto Alexithymia Scale (TAS-20) and the Difficulties in Emotion Regulation Scale (DERS). Results: All of the patients exhibited more difficulties in emotional recognition (alexithymia) and regulation than controls. No differences were observed among patients in terms of body mass index (BMI). The intensity of these difficulties was modulated by age and the severity of the disorder. Alexithymia was more intense in adult patients than in adolescent patients, but both groups showed similar difficulties in emotional acceptance and regulation. Severely affected patients (adult and adolescents) exhibited higher levels of alexithymia, rejection, and emotional dysfunction. Conclusion: It is crucial that emotional therapeutic interventions are carried out from the commencement of treatment. Prevention programmes should enhance recognition, emotional acceptance, and regulation in individuals at risk.

1. Salbach, H., Bohnekamp, I., Lehmkuhl, U., Pfeiffer, E., & Korte, A. (2006). Family-oriented group therapy in the treatment of female patients with anorexia and bulimia nervosa-A pilot study. *Zeitschrift fur Kinder- und Jugendpsychiatrie und Psychotherapie, 34*(4), 267-274. <https://doi.org/10.1024/1422-4917.34.4.267>

**Abstract**

Objectives: Family therapy has proven effective in the treatment of anorexia nervosa (AN) and bulimia nervosa (BN) in adolescence. While cognitive-behavioural treatment has been shown to be effective in adult patients suffering from BN, there have been few studies on the effectiveness of psychotherapy in the treatment of adolescents. Since in the majority of AN patients their illness starts in mid-adolescence, and in late adolescence in BN patients, it is crucial to develop and evaluate treatment programmes for these disorders and age groups. In view of these arguments, a programme of group psychotherapy was set up for eating-disordered patients and their parents, combining disorder-specific psychoeducational components with a family group psychotherapy approach that is more open with regard to individual treatment goals. Patients participated together with their parents in the same group. Methods: The treatment programme was evaluated within the framework of a naturalistic single-group study design. Pre and post changes were assessed. Results: 32 female patients (29 with AN, 3 with BN) and their parents took part in the treatment programme. All of the families completed the programme, which was interpreted as a high rate of acceptance. Pre- and post-analyses revealed a decrease in the degree of eating-disorder symptoms. Conclusions: The advantages and disadvantages of this treatment programme, as well as the limitations of the pilot study are critically discussed.

1. Salbach-Andrae, H., Lenz, K., Klinkowski, N., Pfeiffer, E., & Lehmkuhl, U. (2007). Self-mutilation and psychiatric disorders in female adolescents. *Zeitschrift fur Psychiatrie, Psychologie und Psychotherapie, 55*(3), 185-193. <https://doi.org/10.1024/1661-4747.55.3.185>

**Abstract**

The present study intended to identify predicting factors for self-mutilation in female adolescent psychiatric patients. Retrospective data analysis of a population of 957 female patients aged 12 to 18 years assessed by the "Frankfurter Kinder- und Jugend psychiatrisches Dokumentationssystem". 18.8 % of the examined patients showed self-mutilation. A stepwise logistic regression identified "diagnosis" and "comorbidity" as good predictors for self-mutilation. However, ROC-analyses proved that only the variable "diagnosis" can be seen as a good predictive factor. Psychiatric disorders represent an important factor for self-mutilation in female adolescent psychiatric patients, whereas a strong association exists between personality disorders, affective disorder, expansive disorder, anorexia nervosa (purging type) or bulimia nervosa and self-mutilation.

1. Sanchez, N. A., & Gutierrez, C. S. (2014). Executive functions and symptoms associated with eating disorders. *Revista Latinoamericana de Psicologia, 46*(1-3), 189-197. <https://doi.org/10.1016/S0120-0534%2814%2970022-6>

**Abstract**

Recent studies agree in that patients with Eating Disorders (ED) suffer from inefficiencies in Executive Functions as a consequence of the disease. In order to determine whether or not such inefficiencies make part of the endophenotype of ED-not that they result as a mere consequence of ED-69 women were evaluated: 23 diagnosed with ED, 23 with symptoms associated to ED and 23 women without any ED or risks for developing them. All participants were administered the Eating Attitude Test (EAT -40), the Interview for Diagnosis of Eating Disorders-IV (IDED -IV), the Tower of London Test and the Wisconsin Card Sorting Test. The results showed inefficiencies in executive functions-specifically in planning and cognitive flexibility for both the analogous symptoms group and the diagnosed ED group. The findings suggest that these alterations could be present prior to the establishment of the ED, and that they can actually constitute a risk factor.

1. Santos, L. R., & Leal, F. J. V. (2005). Assessment of expressed emotion in families of patients with eating disorders: using the Camberwell Family Interview on a Spanish sample. *Actas Espanolas De Psiquiatria, 33*(6), 359-365.

**Abstract**

Introduction: The level of expressed emotion (EE) in the family members has been related to several clinical and outcome realted factors in patients with eating disorders. This study aimed to study the levels of EE in families of patients with eating disorders using the Camberwell Family Interview (CFI) in order to determine whether they were similar to those reported in other studies developed outside Spain. Methods: Seventy-one parents of 43 eating disorders patients were evaluated using a Spanish version of the CH. Results: In our sample, 46.5% of the families had EE high levels. There were no significant differences between mothers and fathers in global scores, but mothers tended to have higher emotional overinvolvement with the patient. Conclusions: The percentage of families that had high EE in our sample was lower than that reported for families with a member with schizophrenia and slightly higher than that detected in other studies on eating disorders. Mothers tended to have higher EE levels than fathers.

1. Sanz Lopez, Y., Guijarro Granados, T., & Sanchez Vazquez, V. (2005). Evaluation of the anxiety in the disorders of the food conduct by means of the STAIC. *Revista de Psiquiatria Infanto-Juvenil, 22*(3), 74-83. Retrieved from <http://ovidsp.ovid.com/ovidweb.cgi?T=JS&PAGE=reference&D=psyc5&NEWS=N&AN=2006-00615-002>.

**Abstract**

The purpose of this study was to assess the association between eating disorders and anxiety. A comparison has been conducted between level of anxiety in adolescent patients with eating disorders and a control group paired by age, sex and education level. Differences in the same variable between anoretic and bulimic patients have also been analyzed. The Stait-Trait Anxiety Inventory for Children (Spielberger, 1998) has been used to study this subject. The results of the investigation noted that patients with eating disorders showed higher levels of anxiety than the control group. Significant differences between anorexia and bulimia nervosa were not identified.

1. Sayin, A., & Kuruoglu, A. C. (2004). A male anorexia nervosa case and its discussion from dynamic point of view. *Turk Psikiyatri Dergisi, 15*(2), 155-160. Retrieved from <http://ovidsp.ovid.com/ovidweb.cgi?T=JS&PAGE=reference&D=psyc5&NEWS=N&AN=2004-15800-009>.

**Abstract**

In this case report, we represent a male anorexia nervosa case and discuss its psychodynamics in the frame of psychoanalytic theories. Males account for 5-15% of all eating disorders. History of a criticisim or a teasing for fatness by relatives or friends is very common in males before the onset of the illness. Males who are in occupations or athletic pursuits that emphasize body weight, or who work in potential high-risk jobs, and who are homosexual are at increased risk for eating disorders. Potential high-risk jobs include appearence-based jobs, jobs traditionally held by women and food related jobs. Comorbid psychiatric disorders, mainly depression and anxiety disorders, and especially for bulimic males, substance-related disorders and cluster B personality traits with marked impulsivity are common. When family dynamics are considered, a father who is psychologically distant from his son, and an "over-protective, over-involved and over-dependent" mother have been described. Separation difficulties and lack of boundaries within covertly conflictual relationships have been noted in a number of clinical reports. The only difference in clinical presentation between males and females is that males rarely use medicine in order to lose weight and weight changes throuhout the illness are more common in males. In this case report, a male anorexia nervosa case's psychodynamics are discussed under the influence of his psychiatric illness and his childhood history.

1. Schepank, H. (1991). Hereditary determinants of anorexia nervosa: Results of studies of twins. *Zeitschrift fur Psychosomatische Medizin und Psychoanalyse, 37*(3), 265-281. Retrieved from <http://ovidsp.ovid.com/ovidweb.cgi?T=JS&PAGE=reference&D=psyc3&NEWS=N&AN=1992-85186-001>.

**Abstract**

Suggests that, contrary to many widely-discussed theories of anorexia nervosa, the conclusive results of numerous studies of twins in favor of the genetic concordance hypothesis have been ignored, and individual case findings have been misinterpreted. Presented here are a 1985 twin study by A. H. Crisp et al and a 30-yr investigation by the present author. These 2 studies, totally independent from each other, show proof of genetic determinants of anorexia nervosa in twins sharing manifestations of the anorexia pathology. Some remaining diagnostic uncertainties are also discussed.

1. Schneider, N., Frieler, K., Ehrlich, S., Pfeiffer, E., Lehmkuhl, U., & Salbach-Andrae, H. (2008). Personality style and assessment of dimensions in adolescent patients with anorexia and bulimia nervosa. *Zeitschrift fur Klinische Psychologie und Psychotherapie: Forschung und Praxis, 37*(4), 236-244. <https://doi.org/10.1026/1616-3443.37.4.236>

**Abstract**

Background: Personality variables can have a bidirectional relationship with eating disorder diagnoses: as risk factors for its development and/or as consequences of the disorder. Personality attributes that have been substantiated to be “typical” of eating disorders are relevant for diagnostics and therapy. Objective: We addressed the question on how patients with anorexia (AN) and bulimia nervosa (BN) differ regarding their personality styles. Method: Personality styles and dimensions were assessed in 104 female adolescent patients (65 AN – restricting type, 19 AN – binge-purging type, 20 BN; *M*_age_ = 16.4 ± 1.7). Results: Explicit tendencies towards compulsive personality styles were found among restricting AN patients, while BN patients showed negativistic and borderline styles. Conclusion: Significant differences between eating disorder groups were found. This indicates a psychopathological association between the type of eating disorder and personality, which may be relevant in terms of pathogenesis, maintenance, and therapy of eating disorders.

1. Schneider, N., Korte, A., Lenz, K., Pfeiffer, E., Lehmkuhl, U., & Salbach-Andrae, H. (2010). Subjective evaluation of DBT treatment by adolescent patients with eating disorders and the correlation with evaluations by their parents and psychotherapists. *Zeitschrift fur Kinder- und Jugendpsychiatrie und Psychotherapie, 38*(1), 51-57. <https://doi.org/10.1024/1422-4917.a000006>

**Abstract**

Objective: The subjective evaluation of a psychotherapeutic treatment may be an essential aspect of the recovery from an eating disorder. Our study investigates the subjective treatment evaluation by patients with eating disorders and the accordance with their parents’ and psychotherapist’s evaluation. Method: In a sample of 41 eating-disorder patients (M_Age_ = 16.3; SD = 1.26), their parents and psychotherapists, we used a questionnaire (FBB) to assess satisfaction with Dialectical Behavioral Therapy treatment. Results: Our results show good ratings, as well as good correlations between patients and parents. Conclusions: The findings implicate the importance of assessing a subjective therapy rating in addition to objective parameters.

1. Seiffge-Krenke, I. (2019). Eating disorders: Developmental psychological and developmental psychopathological perspective. *Kindheit und Entwicklung: Zeitschrift fur Klinische Kinderpsychologie, 28*(4), 197-209. <https://doi.org/10.1026/0942-5403/a000291>

**Abstract**

The present article deals with various eating disorders such as anorexia, bulimia, and obesity with a focus on anorexia and bulimia, which have a clear onset in adolescence. Because disturbed eating and weight control are also phenomena in normal girls of this age, eating disorders have to be considered from the perspective of developmental psychology and developmental psychopathology. Body image disorders, disturbed eating habits, and the influence of the family are placed in the context of normal girl development. Since 40 years, a more negative body image of girls as compared with boys has been found in research. Further, the more negative effect of early maturation on body image and dieting in girls is a robust finding in many studies since decades. The role of fathers in establishing a more positive body image and as a stress buffer, especially in early maturing girls, has been frequently overlooked in the past, but was, however, established in more recent studies. These studies also show the long-term effects of a positive body concept for partnerships in the future, in young adulthood. Thus, long-term outcomes of a negative body concept are far more significant and affect not only nutrition and weight control. Family support and nonjudgmental, non-body-related commentary on weight and attractiveness given by parents are very important here. Even with non-eating-impaired adolescents, extreme dieting, fearful body control, as well as problematic parental communication styles have been found. Recent studies revealed a rather high prevalence of psychological disturbances of mothers of anorexic and bulimic patients. This underscores that the mothers are particularly challenged when dealing with their daughters’ extreme diets and weight control, which is certainly hard for the mother being diagnosed as depressed, anxious, or having a personality disorder. In this overview, the influence of friends and the new media is also assessed. This indicates often stressful interactions with peers, which are quite typical even in normal girls. Extreme social comparison and rumination as a negative form of communication between girls were described. Therefore, it is difficult to limit the issue to pathological phenomena. In this context, it is important to note that there are high comorbidities between different eating disorders and with internalizing and externalizing mental disorders. The high comorbidity makes it difficult to develop a treatment plan. Historical phenomena such as the “holy anorexia” of the past centuries, the currently observable increase in eating disorders in boys, and the desire to change gender (transgender) are considered, as well as cultural influences (“Is fat beautiful?”). In this context, the question is raised of whether eating disorders have increased and what contribution the new media may have.

1. Sellami, R., Bouattour, W., Baati, I., Feki, I., & Masmoudi, J. (2020). Relationship between alexithymia and impulsivity in a sample of in-school adolescents in Sfax, Tunisia. *Annales Medico-Psychologiques, 178*(9), 920-924. <https://doi.org/10.1016/j.amp.2019.10.005>

**Abstract**

Objectives: The study of alexithymia in adolescence seems particularly interesting for various reasons. First, physical, psychological and social changes create new experiences of emotional reactivation. Second, the adolescent period coincides with the maturation of the hormonal, neuronal, and cognitive systems that underlie the development of emotional regulation. Finally, evidence suggests that alexithymia may have detrimental consequences in adolescents, including substance use disorders, depression, anxiety, behavioral disorders and self-injury. Also, alexithymia is associated with impulsivity because of a deficit in the cognitive processing of emotions with tendencies to act rather than talking about feelings. The purpose of the current study is to estimate the prevalence of alexithymia in a sample of school-aged adolescents in the Sfax region (Tunisia) and to evaluate its links with impulsivity. Materials and methods: Cross-sectional study involving 474 students from public institutions in the Sfax region. Participants completed, after consent, an epidemiological fact sheet, the Toronto Alexithymia Scale (TAS-20) and the Barratt Impulsiveness Scale (BIS-11). Results: The average age of the students was 14.77 ± 1.75 years with extremes ranging from 13 to 18 years old. The sex ratio was 0.96. The prevalence of alexithymia was 35.86% and that of impulsivity 39%. The analytical study showed that alexithymic adolescents had a higher degree of impulsivity (27.4% vs 11.8%, *p* = 0.001) with a positive correlation between TAS20 and Barratt scores (*p* < 10^−3^ ; r = 0,33). Conclusion: Our study shows that alexithymia is common in our adolescents and is associated with impulsivity. The prevention of alexithymia seems essential to reduce the frequency of impulsive behavior.

1. Silva, S., & Maia, A. C. (2010). Adverse childhood experiences and suicide attempts in morbidly obese adults. *Revista de Psiquiatria do Rio Grande do Sul, 32*(3), 69-72. <https://doi.org/10.1590/S0101-81082010005000002>

**Abstract**

Introduction: Suicide attempts are often associated with eating disorders, both anorexia and bulimia. Likewise, a high incidence of suicide has been observed among obese patients. Previous studies have shown that adverse experiences in childhood may be a risk factor for suicide attempts. Objectives: To characterize and to understand the relationship between adverse experiences and suicide attempts in 100 morbidly obese patients referred for bariatric surgery. Methods: A total of 100 patients were selected from September 2007 to October 2007 and from January 2008 to January 2009. Of these, 20 patients were male. Mean age was 38.89±9.87 years, and mean maximum weight was 136.43±14 kg. The Portuguese version of the Family ACE (Adverse Childhood Experiences) Questionnaire was used to assess the occurrence of adverse events. Results: 88% of the patients reported the existence of at least one adverse experience in childhood, and 25% reported at least one previous suicide attempt. Adversity in childhood was associated with an increased risk for suicide attempts (odds ratio = 2.026). Conclusion: These data should be taken into account in the assessment and monitoring of these patients.

1. Silva-Gutierrez, C., & Sanchez-Sosa, J. J. (2006). Family environment, food intake and eating disorders. *Revista Mexicana de Psicologia, 23*(2), 173-183. Retrieved from <http://ovidsp.ovid.com/ovidweb.cgi?T=JS&PAGE=reference&D=psyc6&NEWS=N&AN=2006-22824-003>.

**Abstract**

We explored the relationship between eating disorders, family environment, food accessibility, and family beliefs regarding body shape and weight. 100 women participated. 50 had formal diagnosis of eating disorders and 50 did not. All participants were given a family environment inventory, a food accessibility questionnaire, and a checklist concerned with beliefs about food. Results revealed 5 relevant factors: physical appearance as an important value among family members, discipline and eating patterns, quality of interaction with the mother, quality of interaction with the father, and a supportive and secure family environment. The clinical group showed less variety in food selection associated with beliefs about weight gain. The control participants' diet was more varied, and they tended to base their food choices on direct preferences rather than on their beliefs. Findings suggest that eating disorders could be more related to family beliefs about weight, body shape and body appearance, than to a problematic family environment.

1. Silvestre, C. (1991). The rituals of orality: Unacknowledged banquets. T*opique: Revue Freudienne, 21*(48), 329-346. Retrieved from <http://ovidsp.ovid.com/ovidweb.cgi?T=JS&PAGE=reference&D=psyc3&NEWS=N&AN=1992-85799-001>.

**Abstract**

Suggests that all food is consumed in a triple register: as satisfaction of a vital need for nutrition; in the realm of imaginary and affective relationships of love and pleasure, or hate and rejection; and in the register of symbolic exchanges. Thus, the intensity of the 1st experiences of childhood satisfactions in contact with the mother's body can be retraced. Eating disorders, such as anorexia and bulimia, evoke a time when equivalences were established (e.g., between incorporation and destructivity). These 2 seemingly opposite phenomena are the 2 faces of a dysfunctional relation with the object.

1. Smith, G. T., Cyders, M., Fischer, S., & Simmons, J. (2004). Integrating dispositional and psychosocial learning risk factors for bulimia nervosa. *Psicologia Conductual: Revista Internacional de Psicologia Clinica de la Salud, 12*(3), 463-489. Retrieved from <http://ovidsp.ovid.com/ovidweb.cgi?T=JS&PAGE=reference&D=psyc5&NEWS=N&AN=2005-01194-006>.

**Abstract**

We propose a risk model for bulimia nervosa (BN) that has these characteristics. Trait urgency, the tendency to act rashly/maladaptively when distressed, is a dispositional risk factor that is general to many disorders, including BN. Expectancies for reinforcement from eating and from dieting/thinness are risk factors that result from psychosocial learning and are specific to eating disorders. Risk for BN is increased to the degree that one is high in negative affectivity, high in urgency, and simultaneously holds expectancies for reinforcement from eating and from dieting/thinness. We provide empirical support for both the dispositional and learned components of this model. We demonstrate how the model integrates and extends existing risk literature and we briefly consider the role of culture in this process.

1. Sorrentino, D., Piegari, G., Amati, G., Mucci, A., & Galderisi, S. (2002). Styles of attachment and cognitive functions in a group of patients with eating disorders. *Psichiatria e Psicoterapia Analitica, 21*(1), 15-26. Retrieved from <http://ovidsp.ovid.com/ovidweb.cgi?T=JS&PAGE=reference&D=psyc4&NEWS=N&AN=2002-17418-001>.

**Abstract**

Studied attachment styles, cognitive functioning, and their relationship in Ss with eating disorders and normal control Ss in Italy. 34 females with bulimia and 16 females with anorexia nervosa (aged 17-41 yrs) and 40 normal females (aged 16-42 yrs) were administered tests of executive functions, attention, short-term memory, and learning of supraspan recurrent sequences. Attachment styles were measured in 23 patients and 25 control Ss with the Bartholomew Scale, the Attachment Style Questionnaire, and the Parental Bonding Instrument. The results suggest that patients with eating disorders have an insecure attachment style associated with a dysfunction of incidental/automatic nonverbal learning.

1. Souza, L. V., & dos Santos, M. A. (2010). Family participation in the treatment of eating disorders. *Psicologia em Estudo, 15*(2), 285-294. <https://doi.org/10.1590/S1413-73722010000200007>

**Abstract**

This study aims to understand the meanings about the family participation in the treatment produced in a support group for the families of people diagnosed with anorexia and bulimia. The participants of this study were families attended at a specialized health care service. The data was constituted by the audio recorded registrations of the group session, which were analyzed through the delimitation of its different interactional sections according to this study purposes. The social constructionism approach was used as theoretical and methodological reference and was used to data analysis. The results permitted understanding that the different descriptions about the relation between the family and the treatment can restrict the different possibilities to give meanings to the families' participation in the treatment. We conclude that the group may function as a significant space to observe the meaning production in statu nascendi, making possible to explore the construction and reconstruction of alternative meanings, that may enrich the interpretative possibilities of each participant.

1. Souza, L. V., & dos Santos, M. A. (2012). Family members of persons diagnosed with eating disorders: Participation in a group setting. *Psicologia: Teoria e Pesquisa, 28*(3), 325-334. <https://doi.org/10.1590/S0102-37722012000300008>

**Abstract**

The biomedical discourse focused on diagnosis has often been used as an exclusive alternative to inform treatment modalities for families of patients with anorexia nervosa and bulimia nervosa. This study aims to increase the understanding of how these families build justifications for their participation in a family support group in the eating disorder treatment context. Social constructionist discourse was used to analyze a session of the group in which the topic of our interest was addressed. The analysis highlighted co-produced meanings about the absence of some families in the group, the decrease of frequency of parent participation, the function of the group, the ideal frequency of family members, and the possibility of family members and coordinators co-construct the group conversational setting.

1. Souza, T. R., Campos, P. F., Almeida, M., Faria, V. M., Chaves, B. S., Faria, W. M., & Valentim-Silva, J. R. (2019). Progressive exercise of very short duration has a potent effect on working memory, inhibitory control and fine motor skills of sedentary young adults. *Motricidade, 15*(Suppl 3), 154-163. Retrieved from <http://ovidsp.ovid.com/ovidweb.cgi?T=JS&PAGE=reference&D=psyc18&NEWS=N&AN=2019-78912-006>.

**Abstract**

Aerobic or resistive exercise has been tested on the performance of executive functions, human exercise, but exhaustive physical exercise still needs to be better investigated. It was our objective to determine the acute effects of a very short duration progressive exercise session on EF and motor skills of healthy young adults. Thirty-six subjects underwent EF tests, Digit Span for working memory, Stroop Test for Inhibitory Control and Digit Tracking for motor before and after performing an exercise session until maximal exhaustion. Statistical analysis was performed using the Shapiro-Wilks test to determine the normality of the data and Student's "t" test with a significance of 5%. The results were shown in mean and standard deviation. An exhaustive physical exercise session was able to improve working memory, inhibitory control and motor skills of young adults. Exhaustive exercise improved EF (working memory and inhibitory control) and motor skills of young adults.

1. Spanou, E., & Morogiannis, F. (2010). The role of family and socio-cultural factors in the development of eating disorders. *Psychiatriki, 21*(1), 41-53. Retrieved from <http://ovidsp.ovid.com/ovidweb.cgi?T=JS&PAGE=reference&D=psyc9&NEWS=N&AN=2010-17198-004>.

**Abstract**

A great number of publications in the international literature have revealed the possible part of biological factors in eating disorders and as well as characteristics of the patients' personality that favour or contribute in the development of these disorders. The research in etiology, however, includes the examination of family and socio-cultural factors. The aim of the present paper was to concentrate bibliographic data related to the family and socio-cultural factors that form the conditions under which anorexia nervosa and bulimia nervosa develop, excluding articles about binge-eating disorder. Articles from 1995 to 2005 were included through search in the files of the electronic database Medline (PubMed) on terms of inventory (MESH) for both disorders. About the role of the family environment, it was found that the factors studied more were family dysfunction, overprotection and sexual or physical abuse. As for the socio-cultural factors it is not perfectly clear whether the western standards of life are linked to the development of these disorders or if there is simply a lack of culturally sensitive instruments to detect these disorders in their different possible forms in the non-western world. An important finding is that there are not enough researches to show clearly any negative part played by the mass media.

1. Speranza, M., Corcos, M., Guilbaud, O., & Jeammet, P. (2000). The alexithymic dimension and psychosomatic risk in eating disorders. *Psichiatria e Psicoterapia Analitica, 19*(3)249-261. Retrieved from <http://ovidsp.ovid.com/ovidweb.cgi?T=JS&PAGE=reference&D=psyc3&NEWS=N&AN=2000-12596-002>.

**Abstract**

Discusses (1) the function of alexithymia in eating disorders and (2) the risk of development of psychosomatic symptoms. Epidemiological data on the prevalence of alexithymia in the general population and in anorexic and bulimic populations are presented. Clinical aspects of alexithymia, the psychopathology of early mother-infant bonds, the effects of chronicity, and the relationship among alexithymia, depression, and dependence behaviors are examined.

1. Stasch, M. & Reich, G. (2000). Interpersonal relationship patterns in families with a bulimic patient: An interaction analysis. *Praxis der Kinderpsychologie und Kinderpsychiatrie, 49*(3), 157-175. Retrieved from <http://ovidsp.ovid.com/ovidweb.cgi?T=JS&PAGE=reference&D=psyc3&NEWS=N&AN=2000-15563-001>.

**Abstract**

Tested basic hypotheses concerning the interaction in families of bulimic patients. Family therapy sessions of 20 female patients of an outpatient therapy unit for eating disorders in Germany were coded with the Gottinger Familieninteraktions-Skalen (G-FIS; microanalytic scales of family interaction) and analysed using the t-test for independent samplings based on testing theories by Neyman and Pearson. Other tests used were (1) the Konflikt-Index (KI; conflict index), (2) the Kohasions-Index (KO; cohesion index); (3) the "Expressivitats"-Index (EX; index of "expressivity"); and (4) "Interpersonelle Grenzen"-Index (IPG; index of interpersonal boundaries for the operationalization of conflictual family interactions. The sibling relationships were systematically included in the study. The parental dyad proved to be more conflictual than the other dyads. A relatively stable pattern of interactional behavior was found between the patient and the parents. The relationship between mother and bulimic daughter was more conflictual than that between father and daughter. The father-sibling dyad was not significantly more conflictual than the father-patient dyad. The need for further studies was discussed.

1. Steinfeld, B., Waldorf, M., Bauer, A., Huber, T. J., Braks, K., & Vocks, S. (2018). Assessment of body-related avoidance behaviour: Validation of the German version of the Body Image Avoidance Questionnaire (BIAQ) in adolescents with anorexia and bulimia nervosa and healthy controls. *PPmP: Psychotherapie Psychosomatik Medizinische Psychologie, 68*(3-4), 126-136. <https://doi.org/10.1055/s-0043-116848>

**Abstract**

Body image avoidance is conceptualised as a behavioural manifestation of body image disturbance, and describes efforts to avoid confrontation with one's own body. While studies have provided hints that body image avoidance in adulthood contributes to the development and maintenance of eating disorders, so far, there are no corresponding findings for adolescence. The Body Image Avoidance Questionnaire (BIAQ) is the most widely used international questionnaire for measuring body-related avoidance behaviour. As its German version has only been validated in an adult sample, the aim of the present study is to statistically test the questionnaire in adolescents with eating disorders. In total, N = 127 female adolescents, including n = 57 with Anorexia Nervosa, n = 24 with Bulimia Nervosa, and n = 46 healthy controls, answered the BIAQ as well as various other instruments for assessing body image disturbance and eating disorder symptoms. The factor structure assumed for the original English version, comprising the higher-order factor "body-related avoidance behaviour" and the 4 subfactors "clothing", "social activities", "eating restraint" and "grooming and weighing", was confirmed by a confirmatory factor analysis. With the exception of the scale "grooming and weighing", all scales showed mostly acceptable internal consistencies, test-retest reliability, differential validity and construct validity. Due to their satisfying psychometric properties, the use of the BIAQ scales "clothing", "social activities" and "eating restraint" can be recommended in research and practice for adolescence.

1. Steins, G., Albrecht, M., & Stolzenburg, H. (2002). Attachment and eating disorders: The importance of internal working models of attachment for the comprehension of anorexia and bulimia. *Zeitschrift fur Klinische Psychologie und Psychotherapie: Forschung und Praxis, 31*(4), 266-271. <https://doi.org/10.1026//1616-3443.31.4.266>

**Abstract**

The central assumption of the present investigation is that a specific internal working model of attachment favors the development of anorexia and bulimia. It is proposed that this internal working model is characterized by an insecure-ambivalent attachment style. This assumption was tested in a group of 51 women suffering from eating disorders and 34 women without eating disorder by measuring attachment style with a questionnaire. The assumption was supported by the results: women with the eating disorders bulimia or anorexia often were disproportionally attached in an ambivalent-insecure manner compared to women without an eating disorder. These results increase our understanding of women suffering from eating disorders.

1. Studt, H. H. (1986). Schizoid versus hysterical personality structure: I. Symptoms and diseases. *Zeitschrift fur Psychosomatische Medizin und Psychoanalyse, 32*(3), 283-295. Retrieved from <http://ovidsp.ovid.com/ovidweb.cgi?T=JS&PAGE=reference&D=psyc2&NEWS=N&AN=1987-07503-001>.

**Abstract**

Compared the symptoms and diseases of neurotics with either a schizoid (n = 38) or hysterical (n = 70) personality structure. Results show that compared to hysterics, schizoids were more frequently male, had a longer duration of illness but earlier referral for psychotherapy, and suffered mainly from psychic symptoms. Complaints of schizoids focused on defective concentration, feelings of apprehension, difficulty in working, bulimia, and the inability to breathe deeply. They were mostly born between 1940 and 1959, and their relatives suffered from psychiatric disorders. In contrast, hysterically structured Ss were almost exclusively women, their 2nd most important structural component being obsessive-compulsive. Their indicating signs manifested themselves more often somatically, and less frequently as anxiety states or phobias. The symptoms occurred simultaneously in several organ systems. Common symptoms were dyspnea and/or hyperventilation tetany, nausea, weight gain, frigidity, and crying fits. Hysterically structured Ss were mainly born between 1920 and 1939. Their relatives had cardiac disorders, and their symptoms were identical or similar to those of their fathers.

1. Szalai, T. D. (2017). The relationship of attachment features and multi-impulsive symptoms in eating disorders. *Orvosi Hetilap, 158*(27), 1058-1066.

**Abstract**

Introduction: Attachment dysfunctions determine borderline personality disorder, which is a frequent background factor of multi-impulsivity; however, the relationship between attachment and multi-impulsive eating disorders is almost unexplored. Aim: To compare attachment features of multi-impulsive and classical eating disorder patients with individuals without eating disorders, and to test attachment as a predictor of multi-impulsivity. Method: A cross-sectional survey (148 females, mean age: 30.9 years) investigated maternal, paternal and adult attachment, depression, anxiety, eating disorder and multi-impulsive symptoms in these groups. Results: Altogether 41.3% of the individuals without eating disorders, 17.6% of classical and 11.8% of multi-impulsive eating disorder patients had secure attachment. Multi-impulsive patients had the most severe eating disorder symptoms (F-(2) = 17.733) and the lowest paternal care (F-(2) = 3.443). Preoccupied and fearful attachment explained 14.5% of multi-impulsive symptoms; however, with adjustment for depression only latter one remained the predictor of multi-impulsivity (t = 5.166, p< 0.01). Conclusion: Multi-impulsives are a distinct subgroup of eating disorder patients from the aspects of both symptoms and attachment. Handling their negative moods may hold therapeutic potentials. Longitudinal studies are required to investigate the therapeutic value of paternal care, attachment preoccupation and fearfulness.

1. Tafa, M., Marzilli, E., Cimino, S., Cerniglia, L., & Bracaglia, F. (2017). Anorexic families and bulimic families: Psychopathological risk and family functioning. *Rassegna di Psicologia, 34*(3), 5-23. Retrieved from <http://ovidsp.ovid.com/ovidweb.cgi?T=JS&PAGE=reference&D=psyc16&NEWS=N&AN=2018-16406-001>.

**Abstract**

The adolescent phase represents a difficult transition of the life cycle for both teenage child and the whole family. The Relational Systemic Theory describes it as a co-evolutionary process in which parents and children are equally involved in this difficult evolutionary challenge. For this reason, the present study aims to investigate both the family functioning and the individual psychopathological features of family members. N = 90 clinic families turned to public and private facilities in Central Italy, seeking support for female adolescents' eating disorders, were divided in two groups according to the symptomatology manifested by adolescent and diagnosed by DSM 5. Two self-reports were given to parents and daughters separately: the SCL-90/R, that examines the psychopathological symptoms of the subjects, and the Faces IV, that measures family functioning. The results show that anorexic families have the worst functioning and adolescents of this group present the most intense psychopathological disturbances.

1. Tetzlaff, A., & Hilbert, A. (2014). The role of the family in childhood and adolescent binge eating-A systematic review. *Zeitschrift fur Kinder- und Jugendpsychiatrie und Psychotherapie, 42*(1), 61-70. <https://doi.org/10.1024/1422-4917/a000270>

**Abstract**

Objective: While family factors in childhood and adolescent anorexia nervosa and bulimia nervosa are well documented and have often been reviewed in the past, less is known about these influences on binge eating without compensatory behavior. This systematic review describes family factors in the development and maintenance of binge eating. Method: A systematic literature search was conducted for studies on associations between binge eating, loss of control eating, and family outcomes. Results: Consistent evidence was found for cross-sectional associations between binge eating and insecure attachment of the child, lower family functioning, and lower parental involvement; for parental unemployment and parental depression as retrospective correlates; and for fewer family meals and more critical comments about weight or shape by parents as variable risk factors. In contrast, rather inconsistent findings referred to the influence of family structures, parental eating disorders, dieting, and their knowledge about child’s eating behavior. Gender differences were identified in association with family relationships and weight stigmatization. Conclusions: As with other eating disorders in youth, the results suggest the importance of familial factors in binge eating. Consequently, family assessment and family-based interventions might be helpful in the treatment of childhood and adolescent binge eating. More research should clarify inconsistent findings using prospective designs.

1. Thiels, C. (2004). Childhood and adolescence of sufferers of eating disorders. *Psychotherapeut, 49*(1), 21-26. <https://doi.org/10.1007/s00278-003-0349-7>

**Abstract**

Depending on definition and sample, a minority of varying size of women with eating disorders report to be victims of sexual and/or physical abuse. These stressful experiences are risk factors for mental disorders in general and not specifically for eating disorders. Parental high expectations and pre-morbid negative self-evaluation seem to be specific risk factors for anorexia nervosa and bulimia nervosa. Probably, patients with bulimia nervosa suffered more childhood adversity than those with restrictive anorexia nervosa. Patients with a history of sexual and/or physical abuse may be more severely ill and more difficult to treat than other patients with eating disorders. Careful questioning about childhood adversity seems advisable. In therapy, maintaining factors - like problems of self-esteem - are more important than predisposing or precipitating factors. For prevention, it is recommended to provide for the safety of children when treating their parents with mental illness - particularly substance use disorders - and to avoid parental high expectations.

1. Thiels, C., & Schmitz, G. S. (2009). Parental assessment of children's eating behaviors with a short form of the Anorectic Behavior Observation Scale. *Zeitschrift fur Kinder- und Jugendpsychiatrie und Psychotherapie, 37*(6), 525-534. <https://doi.org/10.1024/1422-4917.37.6.525>

**Abstract**

Objective: To determine whether the Anorectic Behavior Observation Scale (ABOS) originally developed for clinical purposes can be used in epidemiological studies to assess parents’ rating of their child’s eating behavior. Method: In a study of 1,057 children aged 10–17 years, the children completed the Eating Disorder Inventory, Child Version (EDI-C), while the parents of 922 children completed the ABOS. The ABOS consists of 30 items, divided into three subscales – unusual eating behaviors, bulimia-like behaviors, and hyperactivity. Results: The data revealed a number of psychometric problems of the ABOS, including variance restriction and extreme distributions. This is due to the checklist design of the ABOS, which generates few «yes» responses in nonclinical populations. Using principal components analysis and confirmatory factor analysis, 10 items were selected that had better psychometric characteristics than the 30-item ABOS while maintaining the same level of validity. This resulted in three subscales similar to the original ones, with 4, 2, and 4 items – eating behavior, bulimia-like behavior, and level of activity/fitness. Conclusions: The short form of the ABOS, called Eating and Activity Questionnaire for Parents (EAQP), is a parsimonious instrument for screening parents’ assessments of their child’s eating behavior.

1. Uehara, T., Kawashima, Y., Goto, M., Takeuchi, K., Mikuni, M. & Vandereycken, W. (2001). The utility of the Anorectic Behavioral Observation Scale (ABOS): Its reliability and validity in Japan. *Seishin Igaku, 43*(5), 509-515. Retrieved from <http://ovidsp.ovid.com/ovidweb.cgi?T=JS&PAGE=reference&D=psyc3&NEWS=N&AN=2001-01372-002>.

**Abstract**

Examined the reliability and validity of the Japanese version of the Anorectic Behavioral Observation Scale (ABOS). Ss were 68 female and 1 male eating disorder patients (mean age 20.4 yrs), their 24 family members, 20 female controls (mean age 24.8 yrs), and their mothers in Niigata and Maebashi, Japan. Ss were administered the ABOS, the Eating Disorder Evaluation Scale (EDES), the Eating Disorder Questionnaire Revised (EDQ-R; N. Tohyama, 1992), and the Global Assessment of Functioning. The results show: (1) that the reliability of the ABOS was high; (2) that in the validity of the ABOS, the EDES and the EDQ-R were significantly correlated with the ABOS; (3) that between the Ss and the controls, the total score of the ABOS showed significant discriminate validity. These results suggest that the ABOS is a useful scale for obtaining various information about patients when they cannot visit a hospital or when family intervention is conducted.

1. Unikel, C., & Gómez-Peresmitré, G. (2004). Construct validity of an instrument to search for eating disorder risk factors in Mexican 38 women. *Salud Mental, 27*(1), 38-49

**Abstract**

The main purpose of this paper was to present the construct validity of an instrument for the detection of risk factors for eating disorders in Mexican adolescents and young women, such that professionals in this area have a reliable and valid instrument for the detection of risk factors with qualitative and quantitative specifications, for the discrimination of risk severity in conjunction with decision-making related to prevention and treatment. This has been a theoretical and practical issue lacking issue in our present socio-cultural context. As systematic research on eating disorders is recent in Mexico, and because the low prevalence of these diseases has limited the access to clinical populations, their cause may be explained based on other cultural context's research findings. Therefore it has become an important task to enrich such information with the Mexican eating disordered women's experience, related to cultural issues. It may appear reiterative to develop new assessment instruments for this purpose, considering that international workings are abundant and some of them have been already validated with Mexican populations, for instance the Eating Attitudes Test, a self-report screening test of the symptoms and concerns characteristic of eating disorders, and the Eating Disorders Inventory, that measures traits and symptom clusters presumed to have relevance for the understanding and treatment of eating disorders. However, it is important to highlight the cultural differences of a particular culture and their relationship to psychopathology. The results obtained in different cultures, question the universal meanings for psychological disorders challenging the validity of standardization for non-occidental cultures. These views support the development of appropriate assessment instruments in different cultures. The main contribution of this instrument is that it was developed based on the information provided by Mexican eating disordered patients in relation to the development of their illness. Risk factors associated with the development of eating disorders were assessed in a sample of 332 Mexican adolescents and young women (average age 19.1 years, SD=3.8), high school and college students at public and private schools in Mexico City. The study was carried out in two phases: in the first, the individual (eating behaviors, body weight history, curriculum, dating and violence), psychosocial (self-esteem, body image, depression, personality traits, identity and sexuality) and sociocultural (interpersonal relationships, career choice and values towards the body) factors associated with the development of eating disorders were assessed using a qualitative methodology (in-depth interviews) with 10 Mexican eating disordered patients in treatment at the Eating Disorders Unit of the National Institute of Psychiatry Ramon del Fuente in Mexico City. The data obtained from the first phase, was used to identify the most important issues reported by patients, and questions were developed to assess the different areas; this resulted in the development of a 273 question self-report instrument. The instrument included two types of measurements: questions specifically implemented for this study, and scales previously validated in Mexican populations, such as the CES-D, suicidal ideation scale, and it relationship with mother/father scale, the Pope self-esteem scale, and the Eating Disorders Inventory perfectionism, maturity fears and body dissatisfaction scales. The instrument was initially assessed with a pilot study to determine the correct comprehension of the items and its statistical discrimination, with 31 women diagnosed with eating disorders in treatment at the Eating Disorders Unit, and 30 normal controls with similar ages (X=22.5, SD=7.7), similar schooling curriculum in years (X=12.5, DE=3.3) and BMI (X=22.3, SD=3.6). To achieve these goals, independent samples t-tests were conducted between groups, considering as valid those items that were different at a .05 level. After the pilot study the instrument included 14 areas and 216 questions associated with the development of eating disorders; nevertheless, in this paper only the results from the validation analyses of the 8 Likert scales that comprise the instrument will be reported: depressive symptoms, suicidal ideation, self-esteem, psychological characteristics, relationships with brothers and sisters, with males, with mother and father. Afterwards, reliability analyses and factorial validation were conducted. The results showed a high total internal consistency for all of the scales (Cronbach alphas between .80 and .94), with factorial distributions similar to those obtained in the previous analyses done with other Mexican populations, as well as an internal consistency of the subscales from moderate to high (Cronbach alphas between .60 and .94). The depressive symptoms scale, showed a two-factor distribution that distinguished between depressive mood and somatic symptoms. The suicidal ideation scale obtained an appropriate internal consistency score and in accordance with previous research in Mexican populations, only one factor was obtained that explained a high percentage of the total variance. The self-esteem scale showed a factorial distribution very similar to the one previously obtained in Mexican adolescents, although not all of the statistical scores were adequate, for instance, in the family relationships and anger subscales (Cronbach alphas <.70). The psychological characteristics scale obtained a 4 factor distribution, eliminated the questions related to the measurement of perfectionism, kept the maturity fears questions, and, the cluster of questions that were originally directed to measure body image, yielded two subscales, one related to the actual aesthetical body model, and the other directly related to body dissatisfaction, all of which obtained good statistical scores of internal consistency and explained variance. The scale "Relationship with brothers and sisters", and "Relationship with males" obtained good statistical scores, in a one factor structure that clustered positive and negative questions. The relationship with father scale, showed a 5-factor structure with an appropriate total internal consistency score; nevertheless, 2 of the 5 subscales: father's criticism and father's maltreatment were not conceptually coherent. In the opposite, the other 3 subscales were conceptually good but one of them had a low internal consistency (Fathers support). On the other hand, the distribution of the same questions in the relationship with mother scale had more conceptual coherence and good statistical scores. The only subscale that showed some problem was the one denominated "Worth", it includes four questions: two refer to the daughter's perception of her mother's demands, and two refer to the daughters’ perception of the worth her mother recognizes in her. In both scales, the factor that obtained the highest internal consistency score was "positive affect", that combines questions about the positive issues of the relationship with parents in regard to the expression of affection and interest in daughter's activities. From these analyses a new instrument for non-clinicians was developed directly from the Mexican population, with adequate reliability and validity scores for the sample studied. That will be helpful for the detection of people at risk for eating disorders, and for sub clinical and clinical eating disorder cases. Nevertheless, given its recent development it will have to be tested in future studies to confirm its utility for eating disorders' research in Mexico.

1. Valbuena, A. (2003). Adolescent suicidal behaviour, psychiatric diagnosis and risk factors. *Revista de Psiquiatria Infanto-Juvenil, 20*(2), 59-65. Retrieved from <http://ovidsp.ovid.com/ovidweb.cgi?T=JS&PAGE=reference&D=psyc4&NEWS=N&AN=2004-11644-001>.

**Abstract**

Seventy-one adolescents admitted for suicidal attempts were submitted to statistical analysis. General descriptive data was object of a separate work published elsewhere. Risk factors of suicide were compared to international papers and several factors were founded: bad academic result, familial psychiatric background, familial antecedents of suicidal attempt and unstructured families. Statistical associations were made by chi square method and also logistic regression procedure. Two different groups were found. The first group showed greater severity of the attempt, male sex and disorder of mood as main diagnosis. The second was characterized by mild attempt, repetition of the attempt, female, sex and conduct disorder and bulimia as main diagnosis. An early recognition of both pathologies would permit a better treatment of suicidal behavior in adolescents.

1. Valls, M., & Chabrol, H. (2014). Disordered eating in men: A review of the current literature. *Journal de Therapie Comportementale et Cognitive, 24*(3), 92-97. <https://doi.org/10.1016/j.jtcc.2014.05.001>

**Abstract**

The aim of this article is to summarize current research in this field. A literature review was conducted on the databases of PsycINFO, MEDLINE and ScienceDirect. Articles on samples of males suffering from obesity were excluded due to the specific nature of eating disorders in this population. In the case of articles containing samples of men and women, only data linked to the male sample was analyzed wherever possible. The results of 34 studies published between 2009 and 2014 were analyzed. Finally, although there were studies focusing on possible treatments of male eating disorders, only one evaluated the efficiency of cognitive behavioral treatment of bulimic patients. The results of this study indicated a reduction in symptoms up to one year following treatment. This article also evokes the lack of sensitivity and of information in society and amongst health professionals, as well as the need to carry out studies on the different types of treatment for men suffering from eating disorders.

1. Van Vreckem, E., & Vandereycken, W. (1989). The sibling relationship in family therapy: Experiences with the treatment of patients with eating disorders. *Tijdschrift voor Psychotherapie, 15*(1), 11-18. Retrieved from <http://ovidsp.ovid.com/ovidweb.cgi?T=JS&PAGE=reference&D=psyc3&NEWS=N&AN=1990-76395-001>.

**Abstract**

Discusses the role of siblings in the treatment of youth with bulimia or anorexia nervosa, siblings' reactions to a brother or sister with serious emotional problems, and forms of therapy sessions with siblings. Brief case studies from the authors' practice are presented.

1. Vandereycken, W. (1994). Eating disorders and parental rearing behavior. *Kind en Adolescent, 15*(3), 119-132. <https://doi.org/10.1007/BF03060552>

**Abstract**

Studied the correlation between eating disorders and the characteristics of victims' experiences of upbringing by their parents. The effects of rejection, emotional warmth, and overprotectiveness were assessed. Ss were 59 adolescents and adults with bulimia nervosa or anorexia) and their parents. Ss with eating disorders completed the Parental Bonding Instrument (G. Parker, 1983) and the Own Memories of Upbringing questionnaire (C. Perris et al, 1980). Their parents were interviewed and also completed the Maudsley Marital Questionnaire (W. A. Arrindell et al, 1983) and the Symptom Checklist-90 (SCL-90).

1. Vardar, E., & Erzengin, M. (2011). The prevalence of eating disorders (EDs) and comorbid psychiatric disorders in adolescents: A two-stage community-based study. *Turk Psikiyatri Dergisi, 22*(4), 205-212. Retrieved from <http://ovidsp.ovid.com/ovidweb.cgi?T=JS&PAGE=reference&D=psyc10&NEWS=N&AN=2012-01905-001>.

**Abstract**

Objective: The aims of this study were to determine the prevalence of eating disorders (EDs) in adolescents and the prevalence of comorbid psychiatric disorders in adolescents with EDs. Method: During stage 1 of the study the Eating Attitude Test (EAT) was administered to 2907 randomly selected adolescent students. During stage 2 of the study students with an EAT score > 30 underwent a clinical interview and those diagnosed with an ED (based on DSM-IV criteria) were included in the ED group. The control group included students that were age- and sex-matched with the ED group, were not diagnosed with an ED, and had an EAT score < 30. Psychiatric comorbidity in the ED and control groups was evaluated using the Structured Clinical Interview for DSM-III-R (outpatient and non-patient forms). Additionally, a demographic data form, the Beck Depression Inventory (BDI), and the Beck Anxiety Inventory (BAI) were administered to all the participants. Results: In total, 68 (9 male and 59 female) of the 2907 students met the diagnostic criteria for an ED. Point-prevalence rates were as follows: anorexia nervosa: 0.034%; bulimia nervosa: 0.79%: eating disorder not otherwise specified: 1.51%; binge eating disorder: 0.99%; any ED: 2.33%. None of the male participants were diagnosed with anorexia nervosa or bulimia nervosa. In all, 8 male students were diagnosed with binge eating disorder. The prevalence of comorbid psychiatric disorders was higher in the ED group. Major depression was the most prevalent comorbid disorder in the ED group, followed by generalized anxiety disorder and social phobia. The body mass index, and BDI, BAI, and EAT scores were higher in the ED group than in the control group. Conclusion: The results of this study show that whereas the point prevalence rate for EDs among all the participants was 2.3%, it was 4.03% among the female participants. Moreover, ED not otherwise specified was the most prevalent ED, and binge eating disorder was the most common ED among the males. The prevalence rates in the present study are similar to those observed in Western countries, except for the prevalence rate for anorexia nervosa, which in the present study was lower. Major depression and generalized anxiety disorder were the most prevalent comorbid disorders in the ED group.

1. Vargioni, J. (2008). Father, can't you see I'm bleeding? *Revue Adolescence, 26*(4, 66), 977-989. <https://doi.org/10.3917/ado.066.0977>

**Abstract**

The analysis of the transference and counter-transference of an obese woman's therapy leads to an understanding of the role of a traumatic pubertaire event in the constitution of a bulimic symptom during the adolescence. Dead ends imposed by this experience, a deferred action effect of the infantile, block the pacifying possibility of repression and lead to a reversal/turning round of the drive. This drive fate organizes a melancholic form of the primal scene in which the subject is guilty. It is then the body, as support of the ego and of the incorporated object that is to be attacked and protected. Force-feeding, as a centripetal movement from outside towards inside, appears as a way of opposing a fantasy of emptying and potential seduction represented by menstrual bleeding.

1. Vasquez A. R., Lopez Aguilar, X., Alvarez Rayon, G. L., Ocampo Tellez-Giron, M. T., & Diaz, J. M. M. (2000). Eating disorders and associated factors in dance students. P*sicologia Contemporanea, 7*(1), 56-65. Retrieved from <http://ovidsp.ovid.com/ovidweb.cgi?T=JS&PAGE=reference&D=psyc3&NEWS=N&AN=2000-15044-006>.

**Abstract**

Studied the prevalence of and risk factors for eating disorders in 72 female dance students (aged 10-18 yrs) in Mexico. Data on sociodemographic variables, clinical and psychological symptoms, and eating behavior were obtained using the Eating Attitudes Test-40 (D. M. Garner and P. E. Garfinkel, 1979), the Bulimia Test (M. C. Smith and M. H. Thelen, 1984), the Questionnaire on Influence on the Body Beauty Model (J. Toro et al, 1994), the Body Shape Questionnaire (P. J. Cooper et al, 1987), the Eating Disorders Inventory, and the Interview for the Diagnosis of Eating Disorders-IV (V. Kutlesic et al, 1998). The results show a diagnosis of anorexia nervosa in 13 Ss, bulimia nervosa in 3 Ss, and partial syndromes in 16 Ss. Risk factors for eating disorders in dance students include fear of growing up, body dissatisfaction, and motivation to be thin. Etiological and sociocultural factors associated with eating disorders in dance students are discussed.

1. Vazquez Velazquez, V., & Reidl Martinez, L. M. (2013). The role of the mother in eating disorders: A psychosocial perspective. *Psicologia y Salud, 23*(1), 15-24. Retrieved from <http://ovidsp.ovid.com/ovidweb.cgi?T=JS&PAGE=reference&D=psyc12&NEWS=N&AN=2013-06377-002>.

**Abstract**

A review of published literature on the role of the mother in developing eating disorders is presented, from a psychosocial perspective. Exiting disorders, such as anorexia nervosa, bulimia nervosa and binge eating disorder, are psychiatric pathologies influenced by genetic, psychological, social and environmental factors. Within the family context, it is necessary to clarify the role of the mother in relation to the development and maintenance of maladaptive eating behaviors and body dissatisfaction, frequently present in these disorders. Findings analyses suggest that the main risk factors for the development of maladaptive eating behaviors include: presence of obesity in the mother, restrictive dieting, persuading their daughters to lose or control their weight, excessive importance by mothers to body appearance and shape determined by society, compulsive eating, restrictions by mothers on their daughters’ eating behavior, psychopathology and the presence of an eating disorder in the mother. These factors also acted as likely predictors of dissatisfaction and altered hunger-satiety sensations in their children.

1. Verbeek, D., & Petermann, F. (2019). Eating disorders. *Kindheit und Entwicklung: Zeitschrift fur Klinische Kinderpsychologie, 28*(4), 191-196.

**Abstract**

Eating disorders such as anorexia and bulimia nervosa as well as binge eating disorder are comparatively rare. They are, however, regarded as severe mental illnesses with an onset in adolescence. Anorexia nervosa in particular is associated with serious somatic and mental impairments in children and adolescents. The different forms of eating disorders as well as their prevalence and risk factors are briefly described. Evaluated school-oriented and Internet-based prevention programs are presented. Finally, current cognitive-behavioral and psychodynamic treatment programs are listed.

1. Vieira, L. S., Juzwiak, C. R., & de Oliveira, R. C. (2021). Bulimia, body, and culture: Systematic review from Brazilian periodicals. *PSICO, 52*(4) 1-13. <https://doi.org/10.15448/1980-8623.2021.4.35778>

**Abstract**

This study evaluated the scientific production in Brazilian Health Sciences periodicals regarding bulimia and the body from the Human Sciences perspective. For this, a systematic review of the literature was designed, in which the database Latin American and Caribbean Health Sciences Literature (Lilacs) was used to collect the data. The terms "eating disorders and body", "eating behavior disorders and body", "bulimia and body" were used in the search of the articles, which included in the sample studies published between 2013 and 2017, available in full and published in Brazilian periodicals. The sample consisted of 19 articles which were read in full. As a result, the scientific production on bulimia and body, although interdisciplinary, has a connection with the natural sciences. In this sense, we conclude that there is a limiting the understanding of the phenomenon under the Health Sciences field, which, if extended to the scope of the Human Sciences will have a potential to open new interpretative horizons.

1. Vila, G., Robert, J. J., Jos, J., & Mouren-Simeoni, M. C. (1997). Insulin-dependent diabetes mellitus in children and in adolescents: value of pedopsychiatric follow-up. *Arch Pediatr, 4*(7), 615-22.

**Abstract**

Introduction: To assess the type of mental disorders met in the medical follow-up of insulin-dependent diabetic children (IDDM) and adolescents and their relationships with metabolic control (HbA1C) in young IDDM patients who consult in a department of child psychiatry. Population and Methods: Twenty boys and 37 girls (mean age: 14.7 +/- 4.1 years and mean duration of IDDM: 5.6 +/- 4.3 years were followed during 1 year by the same child psychiatrist (mean duration of follow-up: 22 months). They were assessed with several clinical interviews (mean: three by subject); mental disorders were classified according to the criteria of the Diagnostic and Statistical Manual of Mental Disorders, third edition revised (DSM III-R). Results: The study showed the importance of emotional disorders, 30 patients presenting at least an anxiety disorder and 17 an affective disorder, 11 a major depressive disorder and eight a dysthymic disorder. The most frequent anxiety disorders were phobias and overanxious disorders. Two patients had an anorexia nervosa, one a bulimia nervosa and nine an eating disorder not otherwise specified. There were ten diagnoses of disruptive behaviour disorders, one toxic substance abuse and 11 adaptation disorders. Seven subjects had a reading and writing learning disorder, three a coordination disorder and three a borderline IQ. Familial factors seemed very important. Nine patients had a parent-child problem, four sibling rivalry disorder and two an attachment disorder. Family problems (conflicts, separations, economic difficulties...) were found in 63% of cases. The mother or the father had mental disorders in 24 cases (42%). The diabetic patients with mental disorders had poor metabolic control (HbA1C = 9.9 +/- 2.4%) and ten subjects (18%) had already somatic complications. Some mental disorders were significantly associated with high HbA1C. The poorer metabolic controls were observed for eating disorders. Somatic complications were associated only with IDDM duration. Conclusions: This study shows the presence of typical DSM III-R mental disorders in IDDM children and adolescents, principally emotional disorders, and their association with a higher somatic risk, maximum for eating disorders. It shows the interest of collaboration between diabetologist and child psychiatrist. The exact prevalence of these disorders should be assessed by epidemiological studies.

1. Visani, E., Loriedo, C., Di Nuovo, S., Menichincheri, R., Seravelli, F., Agostino, C., & Ferrara, C. (2020). Differences and similarities between clinical and non-clinical families. A research with Faces IV. *Terapia Familiare: Rivista Interdisciplinare di Ricerca e Intervento Relazionale, 122*, 93-110. <https://doi.org/10.3280/TF2020-122006>

**Abstract**

Purpose: The present study aims to evaluate the differences in family functioning between non-clinical and clinical family samples; in the clinical family sample, there is a member with a psychiatric disorder (anorexia nervosa, bulimia nervosa and a first episode at risk of psychosis). Methods: The non-clinical families sample consists of 50 families and the clinical sample of 60 families and for each family unit the data of both parents and a son were collected. The tools used were: a socio-demographic sheet, a clinical sheet for the clinical population and the FACES-IV questionnaire validated in the Italian version. The FACES-IV questionnaire evaluates family through 8 scales: Balanced cohesion, Balanced flexibility, Disengagement, Enmeshment, Rigidity, Disorganization, Communication and Satisfaction. Results: The comparison between non-clinical and clinical families in Global Family Functioning showed a smaller difference than expected; while values related to parental agreements, family perception by each member and single questionnaire scales values showed statistically significative differences. Conclusions: Clinical and non-clinical family evolutionary pathways follow different ways, even if the differences in global family functioning are less than expected.

1. Vittorio V. C., & Barbera, G. (2017). The new forms of symptom in adolescence. Role of the multifamily group. *Interazioni: Clinica e ricerca psicoanalitica su individuo-coppia-famiglia, 46*(2), 82-95. <https://doi.org/10.3280/INT2017-002008>

**Abstract**

In Mental Health Departments we need a map to organize our work, which should be that of early intervention in psychiatry, the identification of mental states at risk, reconstruction and understanding of pathology and identification of possible and effective interventions. We talk of "vacuum clinic", which includes all those symptomatic manifestations that characterize our age: anorexia, bulimia, obesity, drug addiction, panic attacks, depressions. More generally, in all new symptom configurations, the "removal/return of removal" binomial does not prevail, as is the case in classical neuroscience, but the "distress/defense" binomial. The theme of narcissism plays a main role to define one of the main contents of the vacuum clinic. We are dealing with subjects who have a weak narcissistic constitution, who live their lives as superfluous, senseless, vulnerable, uprooted, unwitting and meaningless. Well, with respect to this narcissistic fragility, the symptom (anorexia, addiction, bulimia, ecc.) works like a kind of narcissistic cement of identity. The new symptomatic and behavioral forms seem to be related to the conflict in which a central role plays an instance that can no longer be identified with the superego but rather with the ideal of the ego, and also with the role the body plays. The ideals of ego appear to be very cruel ideals that force you to despise your body, to punish it, kill it, or otherwise make it disappear to the eyes of others (we have suicide, selfishness, social retreat, eating disorders). The multifamily group becomes the place where it's possible to work over these problems; we can think at the group as a "primary social network" that provides support and availability of new learning when it has produced a sort of "break" in the natural social network.

1. Vu-Augier de Montgremier, M., Chen, J., Guo, K., & Moro, M. (2017). Cultural and transcultural aspects of eating disorders for Chinese female adolescents or young adults. *Neuropsychiatrie de l'Enfance et de l'Adolescence, 65*(3), 146-154.

**Abstract**

Background: Eating disorders were long considered as a culture-bound syndrome specific to western countries. However, studies reported results for anorexia in other cultural areas such as China, where its prevalence and incidence is rising. This suggests that anorexia is rather a culture-change syndrome arising during fast sociocultural changes. Adaptation to those changes may contribute to the increasing prevalence of eating disorders. Furthermore, transcultural psychiatrists consider that acculturation and culture clash (conflict between different cultural models) are risk factors for developing eating disorders; but they are less transcultural studies about bulimia. Clinical cases of eating disorders studied around the world showed a great variability in symptoms and psychopathology. Therefore, the role of cultural played and transcultural factors in the increase in eating disorders needs to be defined. Method and population: Based on a qualitative study of eight clinical cases of eating disorders in China, a country and a society undergoing and experiencing a lot of fast sociocultural changes, our work aims to show the impact of cultural and transcultural factors on eating disorders, and takes into account the psychopathology and the symptomatology. Patients were hospitalized in Shanghai Mental Health Center and observed during the internship of a French resident in psychiatry.

1. Vu-Augier de Montgremier, M., Moro, M. R., Chen, J., & Lachal, J. (2022). Female patients with eating disorders and their parents experience in China: A qualitative study. *L'Encephale: Revue de psychiatrie clinique biologique et therapeutique, 48*(1) 43-51. <https://doi.org/10.1016/j.encep.2021.01.006>

**Abstract**

In this study, we intend to explore the experience of Chinese female patients hospitalized for an eating disorder and the experience of their parents: their relationship with the symptoms, with their health and their understanding of the disease, in order to identify the impact of the Chinese cultural context upon the disorder. Thirteen female patients and 11 parents took part in the study. The patients were between 12 and 31 years of age. They showed a wide range of eating disorders: restrictive anorexia, anorexia nervosa, or bulimia nervosa. Their Body Mass Index at the time of the interview ranged from 11 to 22. Exploring the experience of young Chinese girls or women suffering from eating disorders highlights the complex interactions between the cultural and clinical context of eating disorders. Weight loss is experienced with great difficulty by the young women of our study, who report somatic symptoms and worry about their thinness. Our results show the central position occupied by maintaining oneself in good health in China, for parents and teenagers alike. The rapid sociocultural changes in China are causing importantintergenerational differences, notably between traditional educational modalities and the need for independence of young people who require specific familial therapy.

1. Wilkins, J. (1995). Anorexia nervosa and bulimia: An intervention model that addresses the issues of adolescence. *Revue Quebecoise de Psychologie, 16*(3), 133-158. Retrieved from <http://ovidsp.ovid.com/ovidweb.cgi?T=JS&PAGE=reference&D=psyc3&NEWS=N&AN=1996-02814-004>.

**Abstract**

Presents an overview of epidemiological, theoretical, and diagnostic aspects of anorexia nervosa and bulimia, and presents a model for outpatient and inpatient treatment of these eating disorders in adolescents. The multidisciplinary model is based on a developmental perspective that addresses the specific biological, psychological, and social issues and tasks of adolescence. The roles and contributions of various members of the therapy team are described, and the importance of adapting the model to the individual patient is emphasized.

1. Willenberg, H., Bassler, M., & Krauthauser, H. (1998). Dynamic family-staging models as an etiological factor in anorexia nervosa and bulimia nervosa. *System Familie, 11*(1), 3-9. <https://doi.org/10.1007/s004910050020>

**Abstract**

Neither sociocultural circumstances stressing live events nor circumscript unconscious conflict constellations alone can contribute to sufficient understanding of the etiology of psychogenic eating disorders. The authors discuss these disorders against the background of a complex and interdependent system of family development ("dynamic family-staging model"). This model was demonstrated and empirically proven in 89 patients (43 with anorexia nervosa and 30 with bulimia nervosa). The frequency of expected family constellations did not differ significantly between the 2 groups of patients, but had a more marked tendency than in a group of 16 patients with atypical anorexia or bulimia nervosa. The findings indicate that a greater patient sample is necessary for a more differentiated clinical investigation of the proposed developmental model.

1. Willenberg, H., Porsch, U., Krauthauser, H., & Hoffmann, S. O. (2000). Connections between violation of sexual limits and psychogenic eating disorders: Are sexual boundary violations an etiological factor in anorexia and bulimia nervosa? *System Familie, 13*(2), 51-58. <https://doi.org/10.1007/s004910000033>

**Abstract**

Studied the relationship between the violation of sexual limits and anorexia and bulimia nervosa. Examined were 89 patients (85 females, 4 males; mean age 25.3 yrs) prior to admission at a psychosomatic hospital in Germany. The Ss were evaluated in several sessions, including an initial open psychoanalytical session and follow-up meetings with a psychotherapist, and partner and family sessions. Also administered was the Giesen-Test (Beckmann et al, 1983). Ss were divided into 3 diagnostic subgroups: (1) with anorexia nervosa; (2) with bulimia nervosa; and (3) with an atypical eating disorder. The Ss were further divided into, and statistically compared with a group having experienced latent and actual incest and a group without incest experiences. The results indicate that in the pathogenesis of psychogenic eating disorders, sexual boundary violations, including latent and actual incest, are not to be viewed as isolated etiological factors. They need to be understood as symptoms of a long-standing imbalance in the dynamics of the family constellation, which, in turn, is affected by these symptoms.

1. Wohlfahrt, A. K., Strack, M., & Reich, G. (2022). Family functioning, interpersonal problems and symptom severity in anorexia and bulimia nervosa-Comparison of female patients and high-school students. *Praxis der Kinderpsychologie und Kinderpsychiatrie, 71*(6), 543-563. <https://doi.org/10.13109/prkk.2022.71.6.543>

**Abstract**

Interrelations between dysfunctional family interaction and interpersonal problems and eating behaviors as well as psychic impairment are investigated by comparing a non-clinical sample of N = 97 female highschool students to N = 115 patients with anorexia nervosa (AN) and N = 101 patients with bulimia nervosa (BN) of a specialised outpatient unit. 19,7 % of the highschool students showed clinically significant eating behaviors. 32,3 % of the highschool-students, 35,5 % of the patients with AN and 48,1 % of the patients with BN had committed non-suicidal self-injurious behaviour at least once. All diagnostic instruments applied were able to differentiate the patients with BN from the patients with AN and the highschool students. Patients with BN showed more impairment by their eating disorder, more psychic symptoms, more dysfunctional family relations and more interpersonal problems than patients with AN and controls. Dysfunctional family interaction, interpersonal problems, and their interaction predicted general psychological strain, eating problems and the relative desired weight in a synergetic way. Self-injurious behavior was best predicted by psychic strain, depending on dysfunctional family and interpersonal relations and their interaction. Patients with AN tend to present themselves and their interpersonal relations in a positive way. Future studies using more specific measurements should re-examine the prevalences of self-injurious behaviours in non-clinical adolescent samples, which is to be focussed more intensely in adolescents as well as eating disorders should.

1. Woldt, L., Schneider, N., Pfeiffer, E., Lehmkuhl, U., & Salbach-Andrae, H. (2010). Psychiatric disorders among parents of patients with anorexia nervosa and bulimia nervosa. *Praxis der Kinderpsychologie und Kinderpsychiatrie, 59*(4), 302-313. <https://doi.org/10.13109/prkk.2010.59.4.302>

**Abstract**

Family diseases represent a risk factor in the multifactorial etiology model regarding the genesis of eating disorders. In German-speaking countries only a few studies give attention to this topic. The aim of this study is to investigate the occurrence frequency of psychiatric disorders and personality styles among parents of juvenile patients with eating disorders. Furthermore, a comparison between parents of patients with restrictive anorexia nervosa (AN-R) and parents of patients with bulimia nervosa (BN) is carried out. Psychiatric disorders listed on Axis I and Axis II of DSM-IV (American Psychiatric Association (APA), 1994) and personality styles were assessed in 73 mothers and fathers of 27 patients with AN-R and 13 patients with BN. The results show a high psychiatric strain among parents of patients with AN-R and BN. However, the overall psychiatric strain does not differ among the parents of patients with AN-R and BN. Depressive disorders were more frequently observed among mothers of patients with AN-R. Parents of patients with BN showed higher occurrences of paranoid and schizotypal personality styles. A vulnerability of psychiatric disorders is indicated among parents of patients with eating disorders in general but nonspecific for AN-R or BN.

1. Zitarosa, D., de Zwaan, M., Pfeffer, M., & Graap, H. (2012). Supporting carers of persons suffering from an eating disorder. *PPmP: Psychotherapie Psychosomatik Medizinische Psychologie, 62*(9-10), 390-399. <https://doi.org/10.1055/s-0032-1316335>

**Abstract**

The purpose of this article is to describe the background and procedure of a skills training program provided for carers of patients suffering from anorexia or bulimia nervosa. Caring for someone suffering from an eating disorder is associated with psychological distress and may lead to unhelpful interactive behaviours that maintain the illness. Recent investigations in supporting carers, especially skills sharing workshops that target interpersonal maintaining factors are described. A 5-session training concept in teaching basic skills and information about eating disorders to carers in order to improve caregiving burden and reduce interpersonal maintaining factors like expressed emotions (EE) is currently examined in our department. Design and content will be described in detail. Carers' and sufferers' perceptions of the impact of the sessions and acceptance of the provided skills training are reported.
